# The impact of contextual information on aesthetic engagement of artworks

**DOI:** 10.1038/s41598-023-30768-9

**Published:** 2023-03-15

**Authors:** Kohinoor M. Darda, Anjan Chatterjee

**Affiliations:** Penn Center for Neuroaesthetics, University of Pennsylvania, Philadelphia, PA USA

**Keywords:** Human behaviour, Cognitive neuroscience, Social behaviour, Social neuroscience

## Abstract

Art is embedded in its historical, social, political, and cultural context, and rarely evaluated in isolation. The semantic context created by providing text-based information about an artwork influences how an artwork will be evaluated. In the current study, we investigated how contextual information influences the aesthetic appreciation of artworks. Experiment 1 explored whether contextual information such as artist or technique information influenced aesthetic judgments of abstract artworks by Jackson Pollock. The combination of artist and technique information increased liking and interest for the artworks. Experiment 2 investigated whether contextual information about the artist, technique, or content of representational artworks by Indian and European/American artists influenced aesthetic responses of Northern American participants. We found that artist, content, and technique information compared to no information influenced the aesthetic experience of representational artworks. For both experiments, the effect of contextual information was stronger in participants with little art experience, and those more open to experience, and for artworks from another culture compared to one’s own. In sum, along-with theories of empirical and neuro-aesthetics, the current findings also have implications for aesthetics education and museum curation. It seems crucial to consider the type of artwork, the type of contextual information, its potential to enhance aesthetic experience, and the curatorial background of the museum or exhibition, as well as individual differences of viewers. Artworks that are unfamiliar to its viewers might require more contextual information to have an impact on the viewers, and may lower viewers’ prejudices against artworks/artists originating from an out-group.

## Introduction

In her memorable essay ‘Context’, author and poet Sylvia Plath wrote that the issues of her time that preoccupied her had an influence on the kind of poetry she wrote: any work of art reflects, to some extent, the cultural or societal moment in which it appears^1^. Whether it is Pablo Picasso’s *Guernica* as a symbol of justice and accountability especially in the Basque region of Spain, or Amrita Sher-Gil’s realistic depiction of women in rural India in the 1930s, art is intimately embedded in its historical, social, political, and cultural context. It is not surprising then that a work of art is rarely evaluated in isolation.

Context shapes neural responses ranging from visual perception to social interactions^2,3^. In recent years, mounting evidence suggests that context shapes not just how we perceive the world but also how we evaluate and interpret it^4,5^. The aesthetic triad framework proposed by Chatterjee and Vartanian^6^ describe aesthetic experiences as emerging from a complex interplay between sensory-motor, emotion-valuation, and knowledge-meaning systems in the brain—sensory inputs and responses combine with our affective responses and emotions, and contextualise within our past experiences, surroundings, and culture. In line with this framework, the last two decades of research in empirical and neuro-aesthetics has established that contextual information plays an important role in top-down modulation of aesthetic experiences, shaping how we perceive and evaluate different artistic productions^7,8^. People’s aesthetic preferences and judgments of artistic productions across art forms are influenced by the perceived cultural context (e.g., whether a painting depicts content from one’s own culture; ^9,10^), the title associated with it (e.g., a positive or negative valanced title associated with a music piece, ^11^), and information about its source (e.g., whether a dance choreography is made by a human or an artificial intelligence algorithm; ^12^).

Museums are often conceived as information spaces where the museum building, its architecture, the location of objects in its space, guided tours, and use of text labels are essential in determining what information accompanies an object and is transmitted to the audience^13^. Along with the physical context in which an artwork is encountered (e.g., whether in a museum or on a computer screen), the semantic context created by providing text-based information is an important aspect of how an artwork will be viewed, judged, and evaluated by individuals entering the museum^7,14^. The current study aims to investigate different types of text-based contextual information, and their impact on aesthetic judgments of artworks, as well as the modulation of this effect by the extent of the individuals’ art experience and openness to experience.

Even a word or title that provides a semantic context in the minds of the viewer is sufficient to change their perception and evaluation of the artwork. For example, labelling an artwork as belonging to a ‘gallery’ compared to made by a computer program increased liking for artworks, and increased engagement of emotion-valuation systems in the brain^15^. Futurist artworks with titles with movement-related words were perceived as more dynamic and were liked more as compared to when no movement word was present in the title or when no title was present^16^. Millis^17^ showed that an elaborate title (e.g., “Sabotage”, “Ice Dancing”) which offered a contextualising explanation of the depiction in a representational artwork increased interest and liking for the artwork compared to a descriptive title (e.g., “Curved Lines”, “Dots of Colour”; ^18^) with little art-historical information about the artwork. For abstract artworks, Leder and colleagues^7^ found that under longer viewing times (starting from 10 s), an elaborative title increased understanding for an abstract artwork compared to a descriptive title. In contrast, under shorter viewing times, descriptive titles enhanced understanding of abstract artworks. Understanding was also enhanced by descriptive titles compared to elaborative titles for abstract modern artworks^18^, but no difference in liking for artworks was found. Although differences between descriptive and elaborative titles are not consistent, these findings suggest that titles are influential elements in the aesthetic appreciation of artworks.

Nevertheless, artworks are not just accompanied by titles but are created by artists and embedded in an art historical context^19^. In galleries and museums, as well as in art catalogues, and books, we often see that artworks are accompanied by text-based contextual information about not just the title, but the artist, the content of the artwork, as well as the year of creation, broad genre, style, and applied technique^20^. Swami^21^ investigated the influence of more detailed information regarding the genre, epoch, and specific interpretation of an artwork. He found that content specific information had the most impact compared to no title, and broad genre and titular information for abstract artworks made by Max Ernst and Pablo Picasso^21^. In contrast, no differences were found between detailed descriptive and elaborative information on aesthetic responses to six artworks of Flemish expressionism in a gallery setting^22^. Descriptive information was redundant with what the viewer could observe in the artwork and did not reveal any deeper meaning about the artwork, whereas elaborative information allowed the viewer to interpret the artwork informed by the literature about the artwork, and period-related information. The authors predicted that elaborative information in comparison to descriptive information would increase liking for the artworks as it would lead to a fuller understanding of the artwork. As a ‘no information’ condition was not present, and participants were either exposed to the descriptive information condition or the elaborative information condition (a between-groups design), it is difficult to ascertain the effects of different types of information within the same participants compared to when no information is present.

While these studies show how contextualising information can affect aesthetic responses, the difference between several types of information related to the content, artist, and technique, compared to no information for abstract and representational artworks is yet unclear. Researchers have not investigated the effects of detailed artist and technique-related information separately, and their impact on the perception and appreciation of artworks. While content-related information might increase processing fluency of the artwork^18,22,23^, artist-related information may contextualise the artwork in its art historical context or provide cues about the identity of the artist^10,21,24^. According to the embodiment simulation account of aesthetic experience, viewers simulate the actions of the subject in the artwork and/or the movements of the artist^25^. Thus, technique-related information about how an artist paints the artwork or the artist’s style may enhance knowledge about the movements of the artist, increasing perception of implied motion in and liking for the artworks.

In addition, contextual information may also vary in how it impacts those with more or less art knowledge and experience. Art experience of viewers has an impact on art perception and appreciation. For instance, art experts use more global visual scanning strategies, and rely more on formal analysis, composition, and style, whereas art-naïve participants often use local viewing strategies, and are more concerned with the content of the artwork (cf.^26^). Art naïve participants like representational artworks more than abstract ones, whereas art experts do not show this preference^9^. The impact of contextualising information, therefore, may also not be uniform across people with differing art experience or knowledge. Those with more art experience might be less susceptible to the impact of contextualising information by focusing on the formal composition of the paintings and may already possess some explicit knowledge about the artwork’s content. In line with this possibility, Cleeremans and colleagues^24^ found that the presence of an artist’s name increased aesthetic preferences in art naïve participants but not in art history students with higher art experience and knowledge. Similarly, individuals with more art experience were less likely to be impacted by the cultural context of the artwork and showed a lower ethnocentric bias compared to those with lower art experience^10^. In contrast, contextual information impacted liking ratings similarly for those with higher and lower art knowledge when artworks were presented with descriptive and elaborative titles^7^. Niestorowicz and Szubielska^27^ found that catalogue tests and a guided curatorial tour increased pleasure and understanding of artworks in fine arts students (but no control group was present for comparison). Thus, it is also possible that given their pre-existing experience and knowledge in art, those with higher art experience may be better able to contextualise the artwork and thus be more susceptible to contextual information.

Similarly, openness to experience might also influence how participants are impacted by the presence of contextual information. Openness is often thought to be the personality domain of the aesthetically sensitive, and a predictor of positive aesthetic attitudes as well as visits to museums, reading literature, and art creation and production^28^. Openness to experience is also considered an information-seeking trait, with those higher in openness found to be more sensitive to novelty in artworks^29^. Thus, it is plausible that those higher in openness to experience are more open to learning from contextual information and are therefore more impacted by the presence of contextual information compared to no information.

In the current study, therefore, across two experiments, we systematically tested the preregistered hypothesis that viewers’ aesthetic appreciation is influenced by contextual information. Experiment 1 investigated how contextual information about the artist and the technique modulates aesthetic appreciation of Jackson Pollock’s abstract artworks. Experiment 2 investigated how contextual information about the content, artist, and technique modulates aesthetic appreciation of representational artworks by Indian and European/American artists. Across both experiments, we also investigated the effect of contextual information on those with more and less art experience and openness to experience.

## Hypotheses and predictions

In Experiment 1, we test the hypothesis that aesthetic appreciation of abstract artworks by Jackson Pollock is modulated by contextual information about Pollock and his technique of painting. We chose Pollock’s paintings as they are abstract and highly dynamic^30^, and participants potentially embody the artist’s movements. Specifically, we predict:

- Information about the artist and his technique (separately and together), compared to no information, will increase aesthetic ratings (ratings of liking and interest) but not descriptive ratings (ratings of complexity),
- technique information would lead to higher liking and interest, especially in artworks with more implied motion as participants may embody the movements of the artist once they have knowledge about his style of painting,
- participants lower in art experience and higher in openness to experience will be more susceptible to contextual information than those higher in art experience and lower in openness to experience

In Experiment 2, we test the hypothesis that aesthetic appreciation of representational artworks by European/American and Indian artists is influenced by contextual information. As an exploratory (also pre-registered) analysis, we investigated the effect of artwork culture (i.e., whether the artworks are of Indian or European/American origin). We also included cognitive-affective ‘impact-on-viewer’﻿ ﻿terms (how does this artwork make you think/feel?) derived from a taxonomy describing qualities of artworks, and the cognitive and affective effects artworks can have on viewers^31^. As an exploratory analysis, we also investigated the effect of these eleven impact terms which include: *angry, calm, compassionate, challenged, edified, enlightened, enraptured, interested, inspired, pleasure,* and *upset*. Specifically, we predict:

- Information about the artist, technique, and content compared to no information will increase ratings of beauty and liking, and aesthetic impacts,
- elaborative information about content will be linked to higher ratings of liking, beauty, and higher ratings on aesthetic impact terms compared to descriptive information about the content of paintings,
- participants lower in art experience and higher in openness to experience will be more susceptible to contextual information than those higher in art experience and lower in openness to experience,
- North American participants will show higher ratings of liking and beauty for European/American artworks compared to Indian artworks. Contextual information will influence ratings more for Indian artworks than European/American artworks.

## Method and results

### Open science statement

Across both experiments, we report how the sample size was determined, all data exclusions, and all measures used in the study^32^. For both experiments, data pre-processing, statistical analyses, and data visualisations were performed using R^33^. Data analyses for both experiments were preregistered on the Open Science Framework (Experiment 1: https://osf.io/6s5eu; Experiment 2: https://osf.io/ktafx).

Mixed effects model analyses were executed using the *lme4* package (v.1.1–28) in R v.4.1.2.^33^. Post-hoc tests were executed using the emmeans package (v.1.7.2). We used an alpha of 0.05 to make inferences and controlled for multiple comparisons using Tukey-HSD in post-hoc tests.

## Experiment 1

### Stimuli generation

Stimuli for Experiment 1 included 31 images of abstract artworks by Jackson Pollock used by our laboratory in a previous study (Humphries et al., *in prep*). Images used in the current study are available on our OSF project page. The image set of 31 artworks was drawn from a larger set of 60 Pollock paintings, normed on ratings of liking, motion, interest, and complexity, among other features (on a scale of 1 (low) to 7 (high)).

The 31 Pollock paintings were then divided into three different balanced subsets for the current experiment. To do this sorting, normalized motion, liking, complexity, and interest ratings averaged across participants for each painting were extracted from the norming study. Subsets balanced across average motion, liking, complexity and interest ratings were randomly simulated (1000 simulations) and the best fit was obtained (the R code for this simulation is available on the OSF). Thus, the three balanced subsets of paintings finally used for the current experiment did not differ significantly in mean ratings of motion, liking, interest, and complexity (see Table 1).

**Table 1:** Mean ratings of motion, liking, interest, and complexity across the three subsets of paintings. *N* = number of paintings, *SD* = standard deviation.

Ratings	Subset 1 (N = 10) Mean (SD)	Subset 2 (N = 11) Mean (SD)	Subset 3 (N = 10) Mean (SD)
Motion	5.52 [0.27]	5.52 [0.43]	5.51 [0.57]
Liking	4.00 [0.20]	4.04 [0.30]	4.02 [0.60]
Interest	4.83 [0.30]	4.81 [0.29]	4.76 [0.37]
Complexity	5.40 [0.27]	5.41 [0.47]	5.35 [0.46]
Contextual information	No information	Information about the artist	Information about the technique

Paintings from subset 1 were presented to participants with no contextual information, paintings from subset 2 were preceded by information about the artist, and paintings from subset 3 were preceded by information about the artist's technique of painting taken from online sources (online sources include a combination of information from Wikipedia and Brittanica on Jackson Pollock and his technique; see Tasks and Procedure for more details).

### Sample size justification

We determined the sample size for Experiment 1 based on a simulation-based power analysis approach using the simR package^34^. Based on previous work^7^ that investigated the effect of contextual information on aesthetic appreciation of artworks, the fixed effect for the type of contextual information (four levels: no information, artist information only, technique information only, and both artist and technique information) was estimated at 0.4. We simulated data across a range of sample sizes for the following model (with contextual information as a fixed effect and participants and items as random effects):

Rating ~ 1 + contextual information + 1|sid + 1|itemno

Our power analyses showed that a sample size of 200 participants would have 80% power to detect a medium effect size of contextual information (based on our fixed effect estimate) with four levels of condition (no information, artist information only, technique information only, and both artist and technique information). We therefore aimed to collect 200 Northern American participants (usable datasets) for Experiment 1. Further details on the power analysis are available on OSF.

### Participants

Northern American participants were recruited on Amazon Mechanical Turk (mTurk). Three hundred and seventy-two participants started the experiment on mTurk, out of which 353 participants completed the experiment. To ensure data quality and bot detection in online surveys, we used extremely stringent attention check measures (Newman et al., 2021). As pre-registered, participants were excluded if they did not pass our attention checks (N = 132, see the Tasks and Procedure section below for details on the attention check questions), or were 2 standard deviations above or below the mean time taken to complete the experiment (N = 7). The final sample of participants included 214 American participants (111 men, 102 women, 1 unspecified; Mean_age_ = 36.94, SD_age_ = 10.23, Range = 22–63 years). A demographic distribution of the sample is provided in Table S1 in the supplementary material. Participants provided informed consent, and all study procedures were approved by the University of Pennsylvania IRB. All research was conducted in accordance with the Declaration of Helsinki.

### Tasks and procedure

Participants completed a rating task, followed by questionnaires that assessed their art experience and openness to experience, as well as demographic questions. In the rating task, participants viewed a total of 31 images of abstract artworks by Jackson Pollock, and rated each of them on the following variables:

*Liking*; how much do you like this painting? [1 = do not like at all, 5 = like it very much].

*Interest*; how interesting do you find the painting? [1 = not at all interesting, 5 = very interesting].

*Complexity*; how complex do you think the painting is? [1 = very simple, 5 = very complex].

The rating task was divided into three blocks. All participants started with Block 1 of rating artworks from subset 1 with no preceding information regarding the artist or the technique. In Block 2, half of the participants saw information about the artist and rated artworks from subset 2 whereas the remaining half saw information about the technique and rated artworks from subset 3. In Block 3, those who saw artist information first in Block 2 were presented with technique information and rated artworks from subset 3, whereas those who saw technique information first in Block 2 were presented with artist information and rated artworks from subset 2. Thus, four conditions were presented: in block 1, no contextual information; in block 2, artist information only OR technique information only, and in block 3, both types of information. Thus, all participants were presented with no information and both types of information, and half of the participants saw a subset of artworks with artist information first and the remaining half saw a subset of artworks with technique information first. The order of the artworks in each block, and the order in which ratings were presented after each artwork were randomized across participants. Information about the artist and technique were each followed by an attention check question (see Box 1). Participants who failed to answer correctly to one or both the attention check questions were excluded from the analyses.

Following the rating task, participants completed the art experience questionnaire (AEQ; ^35^), an openness to experience questionnaire, and a demographic questionnaire. To obtain a general measure of the Big Five personality trait of openness to experience, we leveraged recent work examining the content coverage of different openness to experience inventories^36^. We opted to use items from two commonly applied inventories: the 48-item openness to experience scale from the NEO-PI-3^37^ and the 20-item Openness/Intellect scale of the Big Five Aspects Scale (BFAS; ^38^). To avoid asking repetitive questions, unique variable analysis^39^, a network psychometrics approach to detect local dependence, was applied to a previous dataset of 802 participants^40^. The analysis reduced the 68 items to 38 items. These 38 items evenly covered the conceptual space of the openness to experience, representing a broad, general measurement of the construct. The openness to experience scale also included one attention check question “Please select Disagree for this statement.” Participants who did not select the correct option were also excluded from the analyses. For the demographic questionnaire, participants could select multiple options for the question “What is your race? Select one or more.” The question following this was “Did you select more than one choice for the question above?” Participants who chose only one option but then reported that they chose more than one choice were also excluded from the analyses. The entire experiment took less than 30 min for most participants (Mean_duration_ = 21.10, SD_duration_ = 13.13), and participants were paid $4 as compensation.

Box 1: Artist and technique information about Jackson Pollock used in the current experiment **Artist information:**

Paul Jackson Pollock (January 28, 1912–August 11, 1956) was an American painter and a major figure in the abstract expressionist movement. He was born in Cody, Wyoming, in 1912, the youngest of five brothers, and grew up in Arizona and Chico. His father LeRoy Pollock was a farmer and later a land surveyor for the government. During his early life, Pollock experienced Native American culture while on surveying trips with his father. In December 1956, four months after his death, Pollock was given a memorial retrospective exhibition at the Museum of Modern Art (MoMA) in New York City. A larger, more comprehensive exhibition of his work was held there in 1967. In 1998 and 1999, his work was honored with large-scale retrospective exhibitions at MoMA and at The Tate in London.

*Attention check question: Where was Jackson Pollock born? (Wyoming, California, Pennsylvania)*

**Technique information:**

Paul Jackson Pollock (January 28, 1912–August 11, 1956) was widely noticed for developing one of the most radical abstract styles in the history of modern art, detaching line from color, redefining the categories of drawing and painting, and finding new means to describe pictorial space. By the mid-1940s, Pollock introduced his famous 'drip paintings' which involved pouring or splashing liquid household paint onto a horizontal surface, enabling him to view and paint his canvases from all angles. It was also called all-over painting and action painting, since he covered the entire canvas and used the force of his whole body to paint, often in a frenetic dancing style. To produce in Jackson Pollock's 'action painting', most of his canvases were either set on the floor, or laid out against a wall, rather than being fixed to an easel. From there, he used a style where he would allow the paint to drip from the paint can.

*Attention check question: Pollock’s drip painting style is also known as…? (Action painting, Can painting, Dance painting)*

### Data analysis

Experiment 1 set out to answer whether aesthetic experience of Jackson Pollock’s abstract artworks is influenced by contextual information about the artist and his technique. For each of our dependent variables (ratings of liking, interest, complexity), we ran linear mixed effects model with contextual information as the fixed effect, and by-subject and by-item random effects. The categorical variable of contextual information was coded using a simple coding style where every other level is compared to the reference level. For the first model, no contextual information was used as the reference level, and each of the other levels (artist information only, technique information only, both types of information) were compared to the reference level separately. To control for effects of demographic variables, art experience, and openness to experience (OE), we further added age, education, total AEQ score, and total OE score as fixed effects to the model. All continuous variables were centered to the mean by subtracting the mean from every value of the variable. The final model used was:

**Model info_type** <- Rating ~ 1 + contextual information + age + education + AEQ score + OE score + 1|sid + 1|item

As preregistered, we also tested a simpler model with only two levels of contextual information (artist information, technique information) to further probe differences between different types of information. The other fixed effects and random effects’ structure was the same as the previous model. Both the simple model and full model yielded comparable results. For completeness, we report the full models as well as the results from the simple model in the supplementary material.

To address whether art experience as measured by the AEQ modulated the effect of contextual information, we added the interaction between art experience and contextual information (both as categorical variables) as a fixed effect to the model. To do this, we centered AEQ scores. Participants with centered AEQ scores above 0 were categorized as ‘high art experience’ and those with centered AEQ scores below 0 were categorized as ‘low art experience’ participants. Results were similar both when art experience was used as categorical variable (high experience, low experience) or when added to the model as a continuous variable (centered AEQ scores; see supplementary material). For posthoc tests, we evaluated the difference between no information and artist/technique/both types of information separately for high art experience and low art experience participants.

**Model_art_experience** <- Rating ~ 1 + art experience*contextual information + age + education + OE score + 1|sid + 1|item

To address the effects of openness to experience (not preregistered), we further ran an exploratory model with the interaction between contextual information and OE score (included as a categorical variable) as a fixed effect. Participants with centered OE scores below 0 were categorized as ‘low openness to experience’ and those with OE scores above 0 were categorized as ‘high openness to experience.’ Results were similar both when openness to experience was added as a categorical or continuous variable (see supplementary material). For posthoc tests, we evaluated the difference between no information and artist/technique/both types of information separately for high openness to experience and low openness to experience participants.

**Model_openness_experience** <- Rating ~ 1 + openness to experience experience*contextual information + age + education + AEQ score + 1|sid + 1|item

Finally, to test whether contextual information interacts with perceived motion in the artworks, we added the interaction between motion and contextual information as a fixed effect to the model. The motion ratings for each artwork in the current study were taken from the norming study (see section on stimuli generation above) and were averaged across participants (from the norming study) for each artwork. Results were similar both when motion ratings were added to the model as a continuous predictor or as a categorical predictor (high motion, low motion).

**Model_motion** <- Rating ~ 1 + motion*contextual information + age + education + AEQ score + OE score + 1|sid + 1|item

### Results

Table S2-S5 show beta estimates, confidence intervals, and p-values for all predictors for all models for all ratings. Table 2 provides information for liking ratings for the *art_experience* and *openness_experience* models in the main text. As the *art_experience* model includes fixed effects from the *info_type* model, we include details only on the *art_experience, openness_experience,* and *motion* models below. The simpler *info_type* model is reported in the supplementary material.

**Table 2:** Beta values, confidence intervals, p-values for *art_experience* and *openness_experience* model for liking ratings.

Art experience	Liking			
Predictors	Estimates	CI	Statistic	*p*
Intercept	3.53	3.42 to 3.64	61.90	**< 0.001**
Artist Information Only	0.00	−0.10 to 0.10	0.03	0.974
Technique Information Only	0.05	−0.05 to 0.16	0.95	0.342
Both Types of Information	0.09	0.01 to 0.17	2.15	**0.032**
Art Experience	0.83	0.62 to 1.03	7.91	**< 0.001**
Age	−0.04	−0.13 to 0.05	−0.89	0.371
Education	0.04	−0.05 to 0.13	0.85	0.394
Openness to Experience	−0.09	−0.19 to 0.01	−1.84	0.066
Artist Information × Art Experience	−0.10	−0.22 to 0.02	−1.65	0.100
Technique Information × Art Experience	−0.04	−0.16 to 0.09	−0.53	0.595
Both Types of Information × Artist Experience	−0.16	−0.25 to −0.07	−3.38	**0.001**
Marginal R^**2**^	0.147			

Openness to experience				
Predictors	Estimates	CI	Statistic	*p*
Intercept	3.59	3.49 to 3.70	67.23	**< 0.001**
Artist Information Only	0.01	−0.09 to 0.11	0.24	0.807
Technique Information Only	0.05	−0.06 to 0.15	0.89	0.372
Both Types of Information	0.11	0.03 to 0.19	2.54	**0.011**
Openness to Experience	0.02	−0.17 to 0.22	0.23	0.815
Age	−0.00	−0.08 to 0.08	−0.07	0.944
Education	−0.00	−0.08 to 0.08	−0.04	0.968
Art Experience	0.58	0.49 to 0.67	12.79	**< 0.001**
Artist Information × Openness to Experience	0.04	−0.08 to 0.17	0.66	0.508
Technique Information × Openness to Experience	0.03	−0.10 to 0.17	0.47	0.636
Both Types of Information × Openness to Experience	0.18	0.08 to 0.28	3.64	**< 0.001**
Marginal R^**2**^	0.257			

Significant values are in bold.

**Figure 1:**
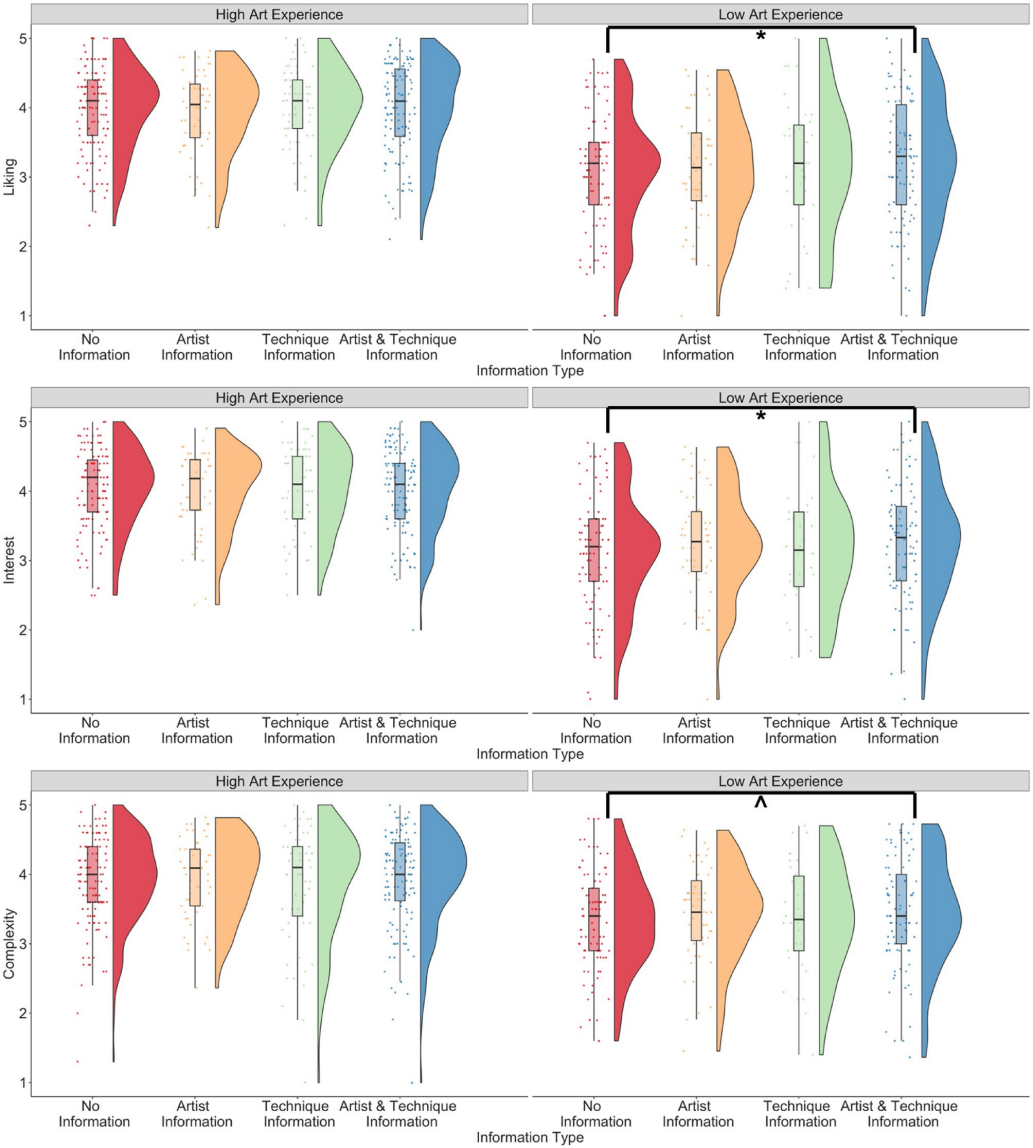
Liking, interest, and complexity ratings when no contextual information was provided to participants, and when information about the artist, information about the technique, and both information about the artist and his technique was provided to participants, split by participants with high and low art experience. *p < .05; ^p = 0.05–0.09.

For the *art_experience* model, results showed that the interaction between art experience and both types of information predicted liking, interest, and complexity ratings (liking: *β* = −0.16, p = 0.001; interest: *β* = −0.20, p < 0.001; complexity: *β* = −0.11, p = 0.015; see Fig. 1). Posthoc tests suggested that compared to no information, both types of information together led to higher liking, interest, and complexity (marginal) ratings but only for participants with lower art experience (liking: estimate = −0.17, SE = 0.05, 95% CI [−0.30, −0.04], p = 0.004; interest: estimate = −0.18, SE = 0.05, 95% CI [−0.31, −0.05], p = 0.003; complexity: estimate = −0.12, SE = 0.05, 95% CI [−0.25, 0.01], p = 0.086) and not for participants with higher art experience (liking: estimate = −0.01, SE = 0.05, 95% CI [−0.13, −0.11], p = 0.99; interest: estimate = 0.02, SE = 0.05, 95% CI [−0.10, 0.15], p = 0.952; complexity: estimate = −0.004, SE = 0.05, 95% CI [−0.12, 0.11], p = 0.999). An interaction between artist information only and art experience predicted interest ratings but not liking and complexity ratings (liking: *β* = −0.10, p = 0.100; interest: *β* = −0.17, p = 0.005; complexity: *β* = −0.08, p = 0.170), although this difference did not hold in posthoc tests when correcting for multiple comparisons. Art experience predicted ratings of liking, interest, and complexity with higher ratings for participants with higher art experience (liking: *β* = 0.83, p < 0.001; interest: *β* = 0.90, p < 0.001; complexity: *β* = 0.55, p < 0.001), and both types of information compared to no information positively predicted ratings of liking and marginally positively predicted ratings of interest but not complexity (liking: *β* = 0.09, p = 0.032; interest: *β* = 0.08, p = 0.08; complexity: *β* = 0.06, p = 0.148). Openness to experience marginally negatively predicted ratings of liking (liking: *β* = −0.09, p = 0.066), no other main effects or interactions were significant. The model explained 14.7% of the variance for liking, 15.4% of the variance for interest, and 6.6% of the variance for complexity ratings.

**Figure 2:**
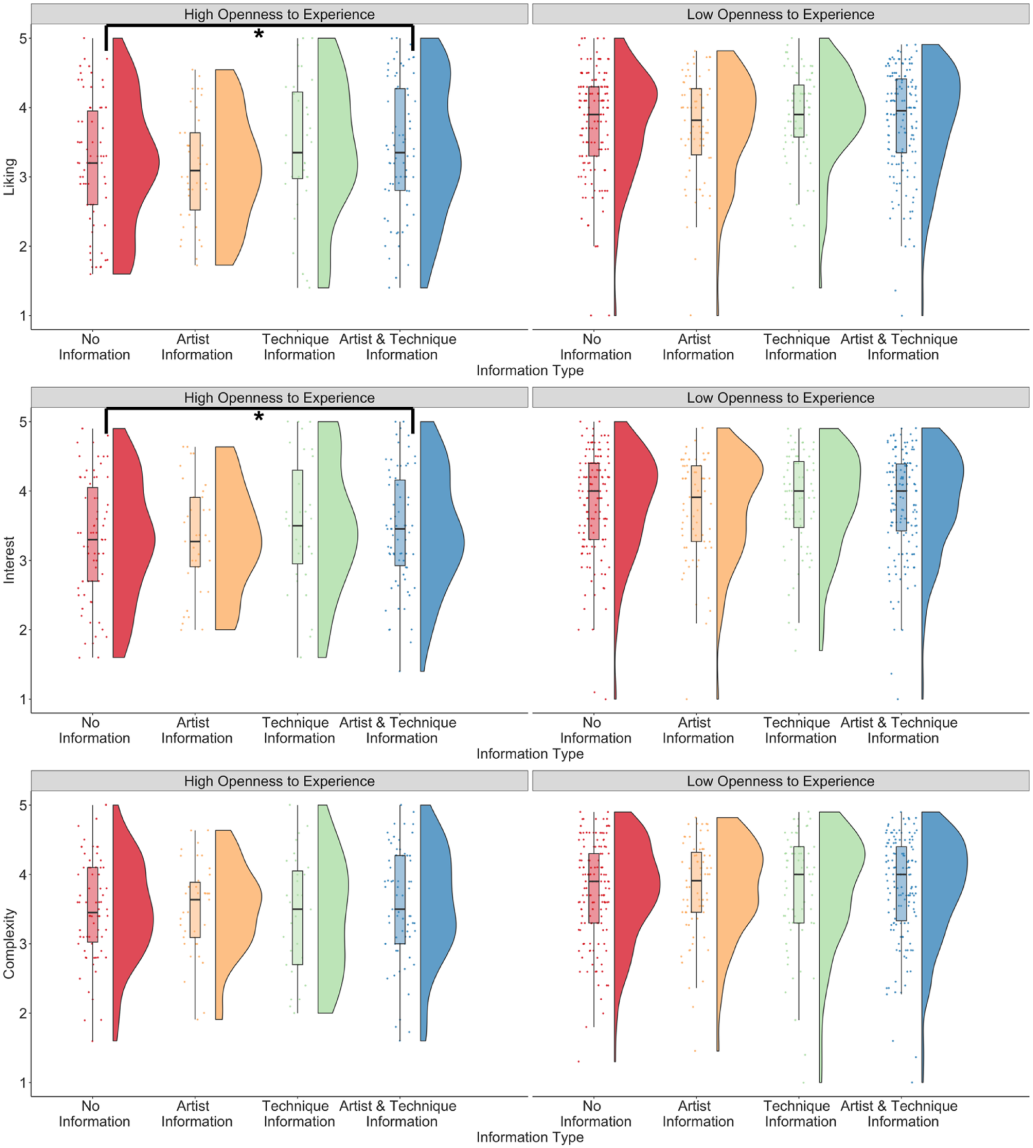
Liking, interest, and complexity ratings when no contextual information was provided to participants, and when information about the artist, information about the technique, and information about the artist and his technique was provided to participants, split by participants who had high and low openness to experience. *p < .05.

For the *openness_experience* model, the interaction between openness to experience and both types of information predicted ratings of liking and interest, but not complexity (liking: *β* = 0.18, p < 0.001; interest: *β* = 0.13, p = 0.007; complexity: *β* = 0.07, p = 0.157; see Fig. 2). Posthoc tests revealed that liking and interest ratings were higher when both types of information were presented compared to no information only for participants with higher openness to experience (liking: estimate = −0.20, SE = 0.05, 95% CI [−0.34, −0.06], p = 0.001; interest: estimate = −0.15, SE = 0.05, 95% CI [−0.29, −0.01], p = 0.027) and not for participants with lower openness to experience (liking: estimate = −0.01, SE = 0.04, 95% CI [−0.13, 0.10], p = 0.976, interest: estimate = −0.017, SE = 0.05, 95% CI [0.14, −0.37], p = 0.982). The interaction between technique information and openness to experience predicted ratings of complexity but not liking and interest (liking: *β* = 0.03, p = 0. 636; interest: *β* = 0.10, p = 0.128; complexity: *β* = −0.13, p = 0.050) but the difference did not hold in posthoc tests when controlling for multiple comparisons. The main effects of age, education, art experience, and information type were similar to the *art_experience* model and are reported in Table S3. This model predicted 25.7% of the variance for liking, 25% for interest, and 11.5% for complexity ratings.

For the *motion_model,* both types of information compared to no information predicted liking but not interest and complexity ratings (liking: *β* = 0.09, p < 0.018; interest: *β* = 0.06, p = 0.165; complexity: *β* = 0.05, p = 0.249). Art experience continued to predict liking, interest, and complexity ratings positively (liking: *β* = 0.58, p < 0.001; interest: *β* = 0.57, p < 0.001; complexity: *β* = 0.39, p < 0.001), and openness to experience positively predicted complexity and interest (marginal) but not liking ratings (liking: *β* = 0.05, p = 0.287; interest: *β* = 0.08, p = 0.062; complexity: *β* = 0.12, p = 0.011). The interaction between artist information and motion marginally predicted liking ratings but not interest and complexity ratings (liking: *β* = −0.15, p = 0.085; interest: *β* = −0.02, p = 0.848; complexity: *β* = −0.02, p = 0.782). Posthoc tests suggested that providing both types of information led to marginally higher liking ratings compared to no information, but only for paintings with low motion (estimate = −0.14, SE = 0.06, 95% CI [−0.29, 0.01], p = 0.072), and not for those with higher motion (estimate = −0.04, SE = 0.05, 95% CI [−0.18, 0.09], p = 0.833). No other main effects or interactions were significant. The model predicted 25.8% of the variance for liking, 25% for interest, and 12.5% for complexity ratings.

### Discussion

In Experiment 1, we set out to investigate whether contextual information such as artist or technique information influenced aesthetic judgments of abstract artworks by Jackson Pollock. We found a small effect of contextual information on aesthetic ratings. Specifically, we found that the combination of artist and technique information (but not artist or technique information in isolation) increased liking and interest ratings, and mostly for participants with lower art experience, and those with higher openness to experience. The effect sizes, however, were small. We also did not find any effect of technique information on artworks with low or high motion. Contrary to our predictions, we did not find an overall effect of artist or technique information only compared to no information.

One explanation for these limited effects could be that participants saw the information about the artist or the technique before rating a set of 10 or 11 artworks. It is possible that participants forgot the information provided to them at the beginning by the end of the rating block. The artworks were also all abstract artworks by a single artist – Jackson Pollock. Thus, even though artist or technique information was provided to participants, they already had rated 10 artworks with no information and continued to evaluate the next set of artworks (whether presented with artist or technique information) in a comparable manner to the first set of artworks with no information and were not as influenced by contextual information. The block order was fixed in Experiment 1 (no information followed by one type of information and then both types of information). Thus, it is difficult to disentangle the effect of mere exposure to stimuli across the blocks from the effect of contextual information. Finally, it is also possible that the information provided about the artist and technique was not as influential as information that might be provided about the content of the artwork.

Therefore, in Experiment 2, we used a wider set of representational artworks made by various artists. We further provided participants with artist, technique, or content information about each artwork before participants rated each artwork to ensure that contextual information was read before each single artwork as opposed to a set of artworks. We also ensured that blocks were randomized to ensure our results did not reflect exposure effects. That is, the order in which no information, and different types of contextual information were presented was randomized across participants. Finally, since broader valuations of liking, interest, and complexity may not fully capture the aesthetic evaluations of participants, we also included 11 aesthetic impact terms that participants rated on (what the artwork made them think or feel) including: angry, calm, compassionate, challenged, edified, enlightened, enraptured, inspired, interested, pleasure, and upset. Participants also continued to rate on liking and complexity, and additionally rated each artwork on beauty.

## Experiment 2

### Stimuli generation

Stimuli for Experiment 2 included 16 images of representational artworks by various Indian and European/American artists (see supplementary material for details about each artwork). Images not protected by copyright restrictions are available on our OSF project page. The image set of 16 artworks was drawn from a larger set of 36 artworks used by our lab in a previous study^10^. The set of 36 artworks were normed on ratings of motion, balance, saturation, warmth, depth, and complexity on a Likert scale from 1 (low) to 7 (high). We divided these 36 artworks into four balanced groups (used a random simulation method similar to Experiment 1). The four subsets thus included 9 artworks balanced on the ratings of motion, balance, saturation, warmth, depth, and complexity. We further chose 4 artworks from each subset that included two artworks by Indian and two artworks by European/American artists across a variety of artistic styles and content. Thus, the four balanced subsets of artworks (4 artworks per subset, 2 Indian, 2 European/American) finally used for the current experiment did not differ significantly in mean ratings of motion, balance, saturation, warmth, depth, and complexity (see Table 3). Artworks from subset 1 were not preceded by any contextual information. Each artwork from subset 2, 3, and 4 was preceded by information about the content, artist, and technique respectively (see Tasks and procedure below for more details).

**Table 3:** Mean ratings of motion, liking, interest, and complexity across the three subsets of paintings. *N* = number of paintings, *SD* = standard deviation.

Ratings	Subset 1 (N = 4) Mean (SD)	Subset 2 (N = 4) Mean (SD)	Subset 3 (N = 4) Mean (SD)	Subset 4 (N = 4) Mean (SD)
Motion	5.11 [0.26]	5.00 [0.39]	5.06 [0.42]	4.98 [0.52]
Depth	5.27 [0.18]	5.26 [0.09]	5.32 [0.11]	5.21 [0.13]
Saturation	5.09 [0.35]	5.01 [0.23]	5.27 [0.27]	5.11 [0.22]
Complexity	5.33 [0.14]	5.07 [0.24]	5.30 [0.14]	5.15 [0.08]
Warmth	5.20 [0.27]	5.11 [0.17]	5.26 [0.23]	5.17 [0.28]
Balance	5.29 [0.20]	5.33 [0.16]	5.33 [0.12]	5.27 [0.26]
Contextual information	No information	Information about the content	Information about the artist	Information about the technique

### Sample size justification

Sample size was the same as Experiment 1 since we expected a medium effect with four conditions of contextual information (no contextual information, content information, artist information, technique information). We therefore aimed to collect 200 Northern American participants (usable datasets) for Experiment 2.

### Participants

Participants of Northern American origin were recruited on Amazon Mechanical Turk (mTurk). Four hundred and thirty-six Northern American participants started the experiment on mTurk, and 380 completed it. As pre-registered, participants were excluded if they did not pass our attention checks (N = 182, see the Tasks and Procedure section below for details on the attention check questions), or were 2 standard deviations above or below the mean time taken to complete the experiment (N = 12). The final sample of participants included 198 American participants (96 men, 98 women, 1 non-binary; Mean_age_ = 39.41, SD_age_ = 11.28). Table S6 reports all participant demographics for Experiment 2. Participants provided informed consent, and all study procedures were approved by the University of Pennsylvania IRB. All research was conducted in accordance with the Declaration of Helsinki.

### Tasks and procedure

Participants completed a rating task, followed by questionnaires that assessed their art experience and openness to experience, as well as demographic questions. In the rating task, participants viewed a total of 16 images of representational artworks by Indian and European/American artists, and rated each of them on the following variables:

- *Liking*; how much do you like this painting? [1 = do not like at all, 5 = like it very much].
- *Beauty*; how beautiful do you find the painting? [1 = not at all beautiful, 5 = very beautiful].
- *Complexity*; how complex do you think the painting is? [1 = very simple, 5 = very complex].

Participants also rated each artwork on 11 ‘impact on viewer’ dimensions on a Likert scale from 1 (not at all) to 5 (a great deal) derived from a taxonomy describing qualities of artworks, and the cognitive and affective effects artworks can have on viewers (Christensen et al., 2022). These eleven impact terms were preceded by the statement ‘this artwork made me think or feel…’ and included: *angry, calm, compassionate, challenged, edified, enlightened, enraptured, interested, inspired, pleasure,* and *upset*. The order in which ratings were presented was randomized across participants.

The rating task was divided into four blocks. No contextual information was presented before any artworks from subset 1. Each artwork from subset 2 was preceded by information about the content of the artwork, each artwork from subset 3 was preceded by information about the artist, and each artwork from subset 4 was preceded by information about the technique of the artist. The order in which these blocks were presented was randomized across participants. Content information was either descriptive or elaborative (one Indian and one European/American artwork was preceded by descriptive content information, and the other Indian and European/American artwork was preceded by elaborative content information). Descriptive information included describing objects or colours or low-level features in the artwork, whereas elaborative information expanded more on what the artwork depicted. Each piece of information was followed by an attention check question to ensure participants were paying attention to and reading the information presented to them before rating the artwork. Participants who had less than 90% accuracy on the attention check questions were excluded from the analyses. Box 2 shows examples of the artworks and the diverse types of information that were presented to the participants, along with the attention check question that followed the information. The entire experiment took around 30 min for most participants (Mean_duration_ = 31.50, SD_duration_ = 18.56), and participants were paid $4 as compensation.

Box 2: Example artwork and types of contextual information associated with it. All images used in this box are free from copyright restrictions

**Table:** 

Example artwork	Type of contextual information
***An artwork from subset 1***	*No contextual information provided*
***An artwork from subset 2 with ‘descriptive’ content information***	*This painting by John Sloan shows the interiors of McSorley’s bar, one of New York’s oldest bars, with its clientele standing at the bar. It depicts several working-class customers drinking around a wooden bar along with the bartender. The background is lined with paintings and other relics and objects. Dark and muted tones dominate the painting, brightened only by flesh tones, highlights of white and yellow, and dabs of orange and red* *Attention check: McSorley’s bar is in which city? (Options: New York, Philadelphia, Boston, Washington DC)*
***An artwork from subset 2 with ‘elaborative’ content information***	*This painting portrays an allegorical meeting between the artist with his patron. It is an interpretation of two mutually interested and interdependent characters who represent two different social classes and two different but interrelated roles in society. The patron’s status is portrayed by his demeanour and manservant. The artist on the right, is powerfully erect, with his head held high. The artist, whose role it is to wander and have no settled place in society is presented as equal to the man of wealth and social position* *Attention check: Who is the meeting with? (Options: the artist and the patron, two random strangers, the dog and its manservant, the artist and his muse)*
***An artwork from subset 3 with artist information***	*This painting is by artist S. Elayaraja, whose paintings became world renowned for his realistic depiction of Tamilian women, their culture, tradition, and lifestyle. Born in 1979 in a small village in the South of India, Elayaraja was the youngest of eleven children. He drew inspiration from his experiences in a large family and made it part of his identity. He obtained Bachelor and Master of Fine Arts degrees from Kumbakonam and Chennai respectively, specializing in oil paintings, water colours, knife painting, and print making and photography* *Attention check: What is the name of the artist you just read about? (Options: S Elayaraja, SS Rajamouli, Raja Ravi Verma, Swathi Thirunal)*
***An artwork from subset 4 with technique information***	*Louis Janmot’s paintings are a transition between romanticism and symbolism artistic styles, and the flawless finish is combined with a sense of mysticism. He had a preference for symmetry and repetition in his paintings and had a lot in common with pre-Raphaelite paintings in terms of content, colour, design, and emphasis on flowers and nature. He applied a design of well-defined contours, simple and dry colours, and a realism in presentation to his paintings* *Attention check: Louis Janmot’s paintings have a sense of ––– in them. (Options: mysticism, abstract expressionism, cubism, nihilism)*

### Data analysis

Experiment 2 set out to answer whether aesthetic experience of representational artworks is influenced by contextual information about the content of the artwork, the artist, and their technique. For each of our dependent variables (ratings of liking, beauty, complexity, and the 11 impact terms), we ran linear mixed effects model with contextual information as the fixed effect, and subject and item as random effects. The categorical variable of contextual information was coded using a simple coding style where every other level is compared to the reference level. No contextual information was used as the reference level, and each of the other levels (content information, artist information, technique information) were compared to the reference level separately. To control for effects of demographic variables, art experience, or openness to experience (OE), we further added age, education, total AEQ score, and total OE score as fixed effects to the model. All continuous variables were centered to the mean by subtracting the mean from every value of the variable. The final model used was:

**Model info_type** <- Rating ~ 1 + contextual information + age + education + AEQ score + OE score + 1|sid + 1|item

As preregistered, we also tested a content-information-only model with only two levels of content information (descriptive, elaborative) to further probe differences between different types of content information. For the content model, the categorical variable of content information was coded as 0.5 for elaborative content and −0.5 for descriptive content. The other fixed effects and random effects’ structure was the same as the previous model.

**Model content_type** <- Rating ~ 1 + content information + age + education + AEQ score + OE score + 1|sid + 1|item

To address whether art experience as measured by the AEQ modulated the effect of contextual information, we added the interaction between art experience and contextual information (both as categorical variables, similar to Experiment 1) as a fixed effect to the model.

**Model art_experience** <- Rating ~ 1 + art experience*contextual information + age + education + OE score + 1|sid + 1|item

**Model art_experience_content** <- Rating ~ 1 + art experience*content information + age + education + OE score + 1|sid

To address whether openness to experience modulated the effect of contextual information, we further ran an analysis with the interaction between contextual information and openness to experience (included as a categorical variable, similar to Experiment 1) as a fixed effect.

**Model openness_experience** <- Rating ~ 1 + openness to experience experience*contextual information + age + education + AEQ score + 1|sid + 1|item

**Model openness_experience_content** <- Rating ~ 1 + openness to experience experience*content information + age + education + AEQ score + 1|sid + 1|item

As a preregistered exploratory analyses, we tested the effect of the cultural source of the artwork (whether by an Indian artist or European/American artist, henceforth referred to as ‘artwork culture’). To probe how artwork culture interacts with contextual information and art experience to influence aesthetic experience of representational artworks, we added the two-way interaction between artwork culture (Indian, European/American) and contextual information (no information, content information, artist information, technique information) as a fixed effect in the model.

**Model culture** <- Rating ~ 1 + artwork culture*contextual information + age + education + openness to experience + 1|sid + 1|item

Finally, to test whether contextual information interacts with perceived motion in the artworks, we added the interaction between motion (as a categorical variable) and contextual information as a fixed effect to the model. The motion ratings for each artwork in the current study were taken from the norming study (see section on stimuli generation above) and were averaged across participants for each artwork. High motion was coded 0.5, and low motion was coded as −0.5.

**Model motion** <- Rating ~ 1 + motion*contextual information + age + education + AEQ score + OE score + 1|sid + 1|item

### Results

#### Liking, beauty, and complexity

In the main manuscript, we report information in Table 4 for the *art_experience* and *openness_experience* models for liking ratings only. Beta estimates, p-values, and 95% confidence intervals are reported for all models in Tables S7-S14. As the *art_experience* model includes fixed effects from the *info_type* model, we include details only on the *art_experience, openness_experience,* and *motion* models below. Findings for the fixed effects are similar across models.

**Table 4:** Beta values, confidence intervals, p-values for *art_experience* and *openness_experience* model for liking ratings for Experiment 2.

Art experience	Liking			
Predictors	Estimates	CI	Statistic	*p*
Intercept	3.36	3.18 to 3.53	37.11	**< 0.001**
Content Information	0.20	−0.02 to 0.41	1.78	0.075
Artist Information	0.37	0.15 to 0.59	3.36	**0.001**
Technique Information	0.18	−0.03 to 0.40	1.66	0.098
Art Experience	1.65	1.42 to 1.89	13.67	**< 0.001**
Age	0.03	−0.06 to 0.13	0.70	0.486
Education	−0.01	−0.10 to 0.09	−0.12	0.906
Openness to Experience	0.15	0.04 to 0.25	2.68	**0.007**
Content Information × Art Experience	−0.28	−0.44 to −0.12	−3.43	**0.001**
Artist Information × Art Experience	−0.54	−0.70 to −0.38	−6.58	**< 0.001**
Technique Information × Art Experience	−0.29	−0.45 to −0.13	−3.49	**< 0.001**
Marginal R^**2**^	0.282			

Openness to experience	Liking			
Intercept	3.46	3.28 to 3.63	38.08	**< 0.001**
Content Information	0.19	−0.03 to 0.40	1.68	0.093
Artist Information	0.39	0.17 to 0.60	3.51	**< 0.001**
Technique Information	0.18	−0.04 to 0.40	1.63	0.104
Openness to Experience	0.21	−0.04 to 0.45	1.65	0.098
Age	0.03	−0.06 to 0.12	0.59	0.554
Education	−0.03	−0.12 to 0.07	−0.56	0.577
Art Experience	0.71	0.60 to 0.81	12.88	**< 0.001**
Content Information × Openness to Experience	−0.00	−0.17 to 0.16	−0.01	0.990
Artist Information × Openness to Experience	0.28	0.11 to 0.45	3.30	**0.001**
Technique Information × Openness to Experience	0.07	−0.09 to 0.24	0.85	0.397
Marginal R^**2**^	0.290			

Significant values are in bold.

**Figure 3:**
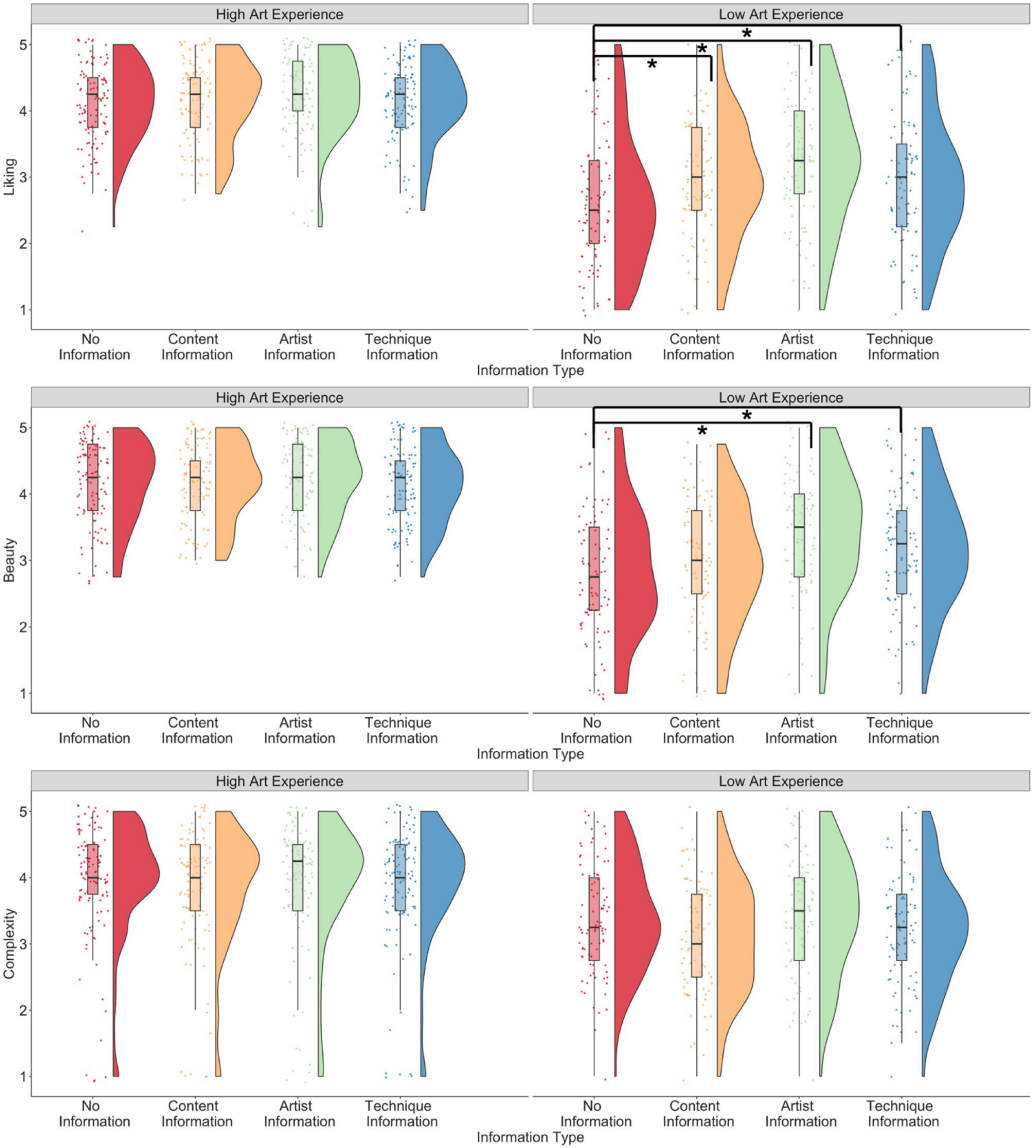
Liking, beauty, and complexity ratings when no contextual information was provided to participants, and when information about the artist, information about the content, and information about the technique was provided to participants, split by participants with high and low art experience. *p < .05.

For the *art_experience* model, results showed that the interaction between art experience and content information predicted ratings of liking, beauty (marginal), and complexity (liking: *β* = −0.28, p = 0.001; beauty: *β* = −0.15, p = 0.065; complexity: *β* = 0.17, p = 0.025; Fig. 3). The interaction between artist information and art experience predicted ratings of liking and beauty but not complexity (liking: *β* = −0.54, p < 0.001; beauty: *β* = −0.58, p < 0.001; complexity: *β* = −0.10, p = 0.177). The interaction between technique information and art experience predicted ratings of liking and beauty, and not complexity (liking: *β* = −0.29, p < 0.001; beauty: *β* = −0.43, p < 0.001; complexity: *β* = 0.11, p = 0.151). Posthoc tests suggested that for liking ratings, information about the content, artist, and technique led to higher ratings compared to no information but only in participants with lower art experience (content: estimate = −0.34, SE = 0.12, 95% CI (−0.64, −0.03), p = 0.02; artist: estimate = −0.64, SE = 0.12, 95% CI [−0.94, −0.33], p < 0.001; technique: estimate = −0.32, SE = 0.12, 95% CI [−0.63, −0.02], p = 0.03), and not in those with higher art experience (all ps > 0.8). For beauty ratings, information about the artist and technique but not content led to higher ratings compared to no information but only in participants with lower art experience (content: estimate = −0.15, SE = 0.12, 95% CI (−0.45, 0.15), p = 0.578; artist: estimate = −0.64, SE = 0.12, 95% CI [−0.94, −0.34], p < 0.001; technique: estimate = −0.37, SE = 0.12, 95% CI [−0.67, −0.07], p = 0.009), and not in those with higher art experience (all ps > 0.9). For complexity ratings, information about the content (and not artist or technique information) led to lower complexity ratings compared to no information only in participants with lower art experience (content: estimate = 0.29, SE = 0.10, 95% CI 0.26, 0.53), p = 0.023; artist: estimate = −0.06, SE = 0.10, 95% CI [−0.31, 0.19], p = 0.940; technique: estimate = 0.16, SE = 0.10, 95% CI [−0.08, 0.42], p = 0.328) and not those with higher art experience (all ps > 0.6).

Art experience positively predicted liking, beauty, and complexity ratings (liking: *β* = 1.65, p < 0.001; beauty: *β* = 1.49, p < 0.001; complexity: *β* = 0.65, p < 0.001), and openness to experience positively predicted liking, beauty, but not complexity ratings (liking: *β* = 0.15, p = 0.007; beauty: *β* = 0.15, p = 0.006; complexity: *β* = 0.08, p = 0.227). The main effect of artist information compared to no contextual information continued to predict ratings of liking, beauty, and complexity (liking: *β* = 0.37, p = 0.001; beauty: *β* = 0.35, p = 0.001; complexity: *β* = 0.00, p = 0.958), content information predicted ratings of liking (marginal) and complexity (liking: *β* = 0.20, p = 0.075; beauty: *β* = 0.08, p = 0.485; complexity: *β* = −0.19, p = 0.030), and technique information marginally predicted ratings of liking but not beauty or complexity (liking: *β* = 0.18, p = 0.098; beauty: *β* = 0.15, p = 0.153; complexity: *β* = −0.11, p = 0.211). No other main effects or interactions were significant. The model explained 28.2% of the variance for liking, 24.8% for beauty, and 8.4% for complexity ratings. 

**Figure 4:**
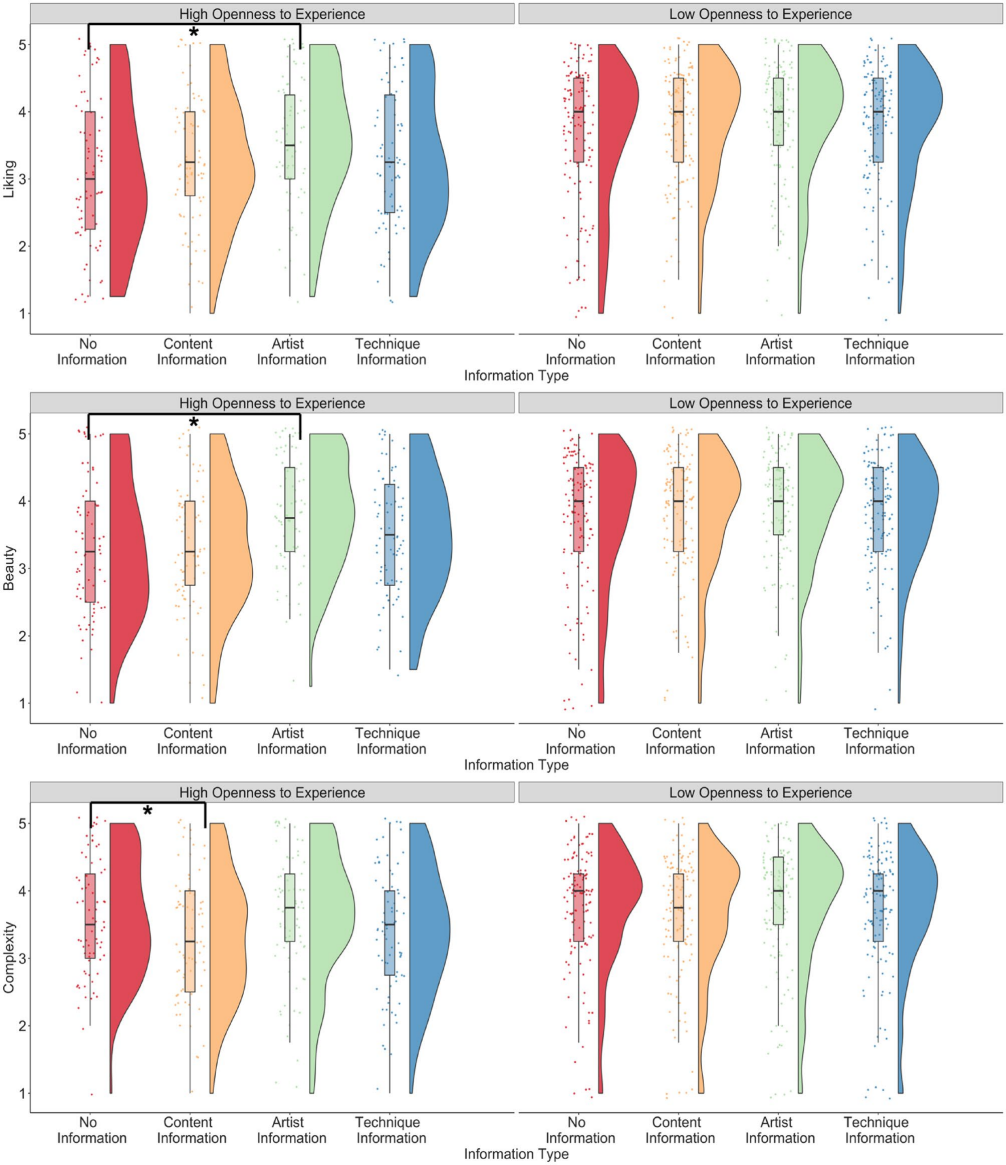
Liking, beauty, and complexity ratings when no contextual information was provided to participants, and when information about the artist, information about the content, and information about the technique was provided to participants, split by participants with high and low openness to experience. *p < .05.

For the *openness_experience* model, the interaction between openness to experience and content information significantly predicted complexity ratings (complexity: *β* = −0.20, p = 0.011), the interaction between artist information and openness to experience was a significant predictor of liking and beauty ratings (liking: *β* = 0.28, p = 0.001; interest: *β* = 0.37, p < 0.001), and the interaction between technique information and openness to experience was a significant predictor of complexity ratings (complexity: *β* = −0.18, p = 0.021; Fig. 4). Posthoc tests suggested that only artist information compared to no information showed higher ratings of liking and beauty but only for participants with higher openness to experience (liking: estimate = −0.53, SE = 0.12, 95% CI [−0.84, −0.21], p = 0.001; beauty: estimate = −0.55, SE = 0.12, 95% CI [−0.86, −0.25], p < 0.001), and not for participants with lower openness to experience (liking: estimate = −0.25, SE = 0.11, 95% CI [−0.54, 0.04], p = 0.132; beauty: estimate = −0.18, SE = 0.11, 95% CI [−0.47, 0.10], p = 0.358). Content information led to lower complexity ratings compared to no information but only in participants with higher openness to experience (estimate = 0.311, SE = 0.10, 95% CI [0.05, 0.57], p = 0.012), and not in those with lower openness to experience (estimate = 0.11, SE – 0.09, 95% CI [−0.13, 0.35], p = 0.626). The main effect of artist information compared to no information predicted liking and beauty ratings (ps < 0.001). Art experience continued to positively predict all ratings (all ps < 0.001) and openness to experience positively predicted beauty (p = 0.045) and complexity ratings (p = 0.025). No other main effects of interactions were significant.

For the *art_experience_content* model, the interaction between content type and art experience was a significant predictor only of complexity ratings and not beauty or liking (complexity: *β* = 0.28, p < 0.001). Posthoc tests revealed that descriptive content compared to elaborative content led to higher complexity ratings only in participants with lower art experience (estimate = 0.27, SE = 0.08, 95% CI [0.11, 0.43], p = 0.001) and not those with higher art experience (estimate = −0.01, SE = 0.07, 95% CI [−0.16, 0.14], p = 0.900). Art experience continued to positively predict beauty, liking, and complexity ratings (all ps < 0.001).

**Figure 5:**
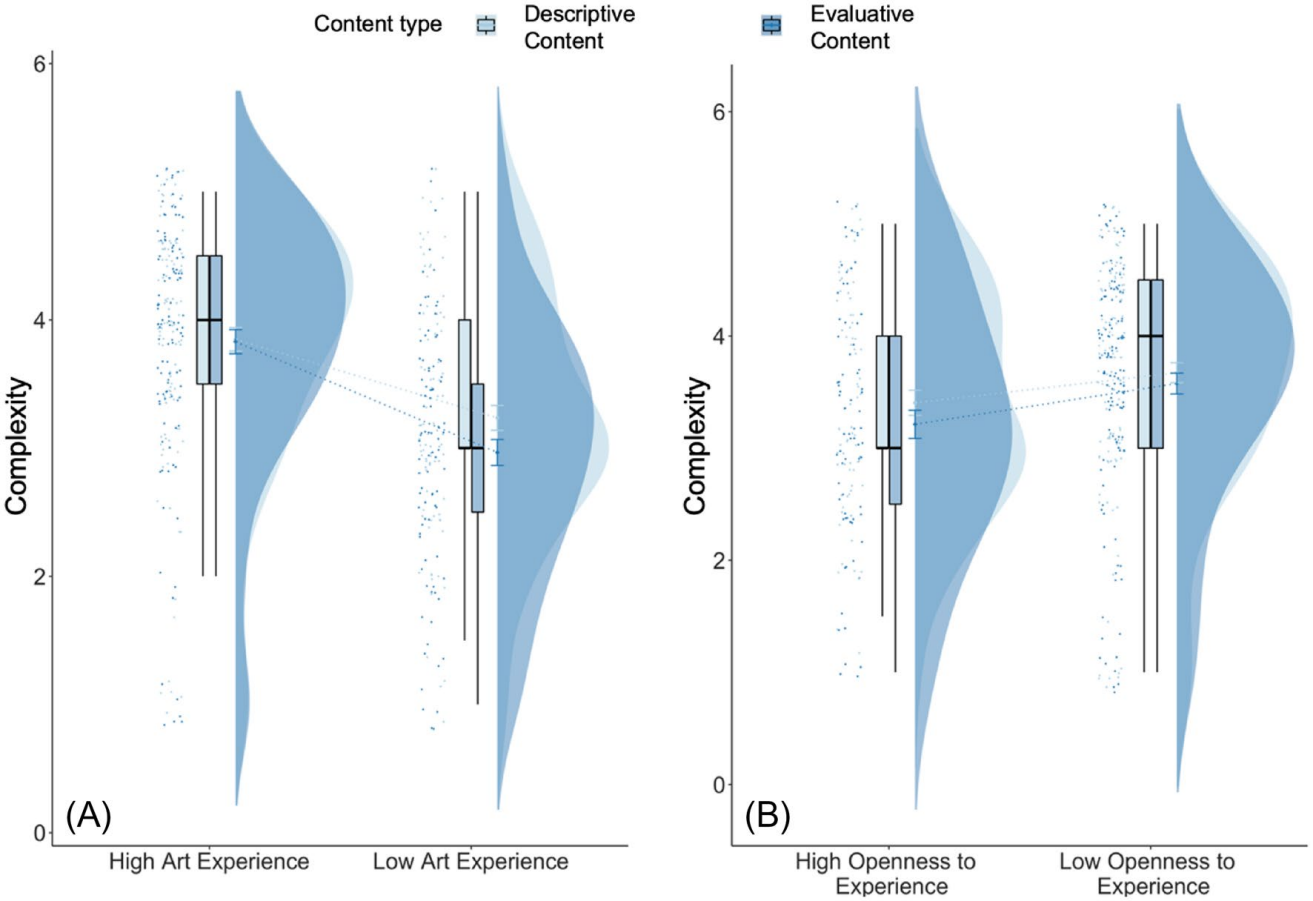
Complexity ratings when participants read either descriptive (in light blue) or elaborative (in dark blue) information prior to rating the artworks for participants with high and low art experience (**A**) and participants with high and low openness to experience (**B**).

For the *openness_experience_content* model, content type predicted ratings of complexity but not beauty or liking (*β* = −0.14, p = 0.012), with lower complexity ratings when elaborative information was presented compared to when descriptive information was presented (estimate = 0.14, SE = 0.06, 95% CI [0.03, 0.26], p = 0.012; Fig. 5). Art experience continued to predict all ratings (ps < 0.001). No other main effects or interactions were significant.

**Figure 6:**
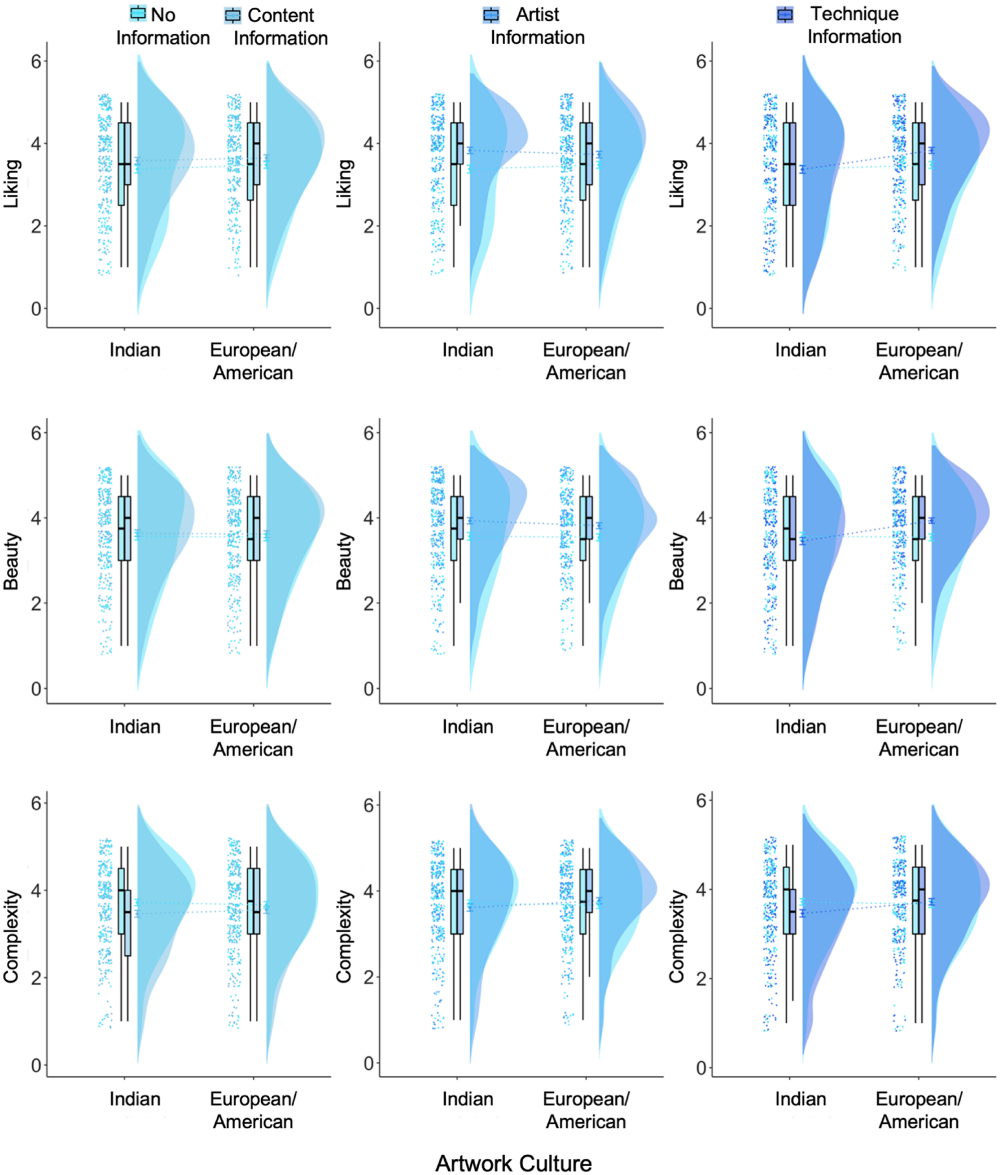
Liking, beauty, and complexity ratings for Indian and European/American artworks when no information is presented to participants compared to when content information (left), artist information (middle), and technique (right) information is presented to participants.

For the *culture* model, the two-way interaction between technique information and artwork culture predicted ratings of liking, beauty, and complexity (liking: *β* = 0.37, p = 0.005; beauty: *β* = 0.51, p < 0.001; complexity: *β* = 0.31, p = 0.018; Fig. 6). We predicted that compared to no information, contextual information would reduce the ingroup bias by increasing liking and beauty ratings (and decreasing complexity ratings) for Indian artworks. Although artist and content information did not show an interaction with artwork culture, we continued to evaluate post hoc tests as per our predictions. We interpret these results with caution. Post hoc tests revealed that artist information led to higher liking and beauty ratings for both European/American (p = 0.042) and Indian artworks (p < 0.001), although the difference was higher for Indian artworks, and technique information led to higher liking ratings compared to no information only for European/American artworks (ps < 0.001) but not Indian artworks (ps > 0.40). Content and technique information compared to no information decreased complexity ratings for Indian artworks (content: p = 0.036, technique: p = 0.026) but not European/American artworks (content: p = 0.595, technique: p = 0.948). Effects of age, education, art experience, openness to experience are similar to the models reported above. The *culture* model predicted 29.9% of the variance for liking, 26.7% for beauty, and 10.6% for complexity ratings.

For the *motion_model,* the interaction between motion and artist information marginally predicted liking ratings (*β* = −0.35, p = 0.052). All other main effects and interactions are reported in Tables S14. Post hoc tests revealed that artist information showed higher liking ratings compared to no information but only for artworks with high motion (estimate = −0.52, SE = 0.13, 95% CI [−0.85, −0.19], p < 0.001) but not for artworks with low motion (estimate = −0.17, SE = 0.13, 95% CI [−0.50, 0.16], p = 0.558). The *motion* model predicted 29.4% of the variance for liking, 25.7% for beauty, and 10.6% for complexity ratings.

#### Aesthetic impacts

Along with ratings of liking, complexity, and beauty, participants rated all artworks on 11 impact terms. Statistics for all models for each impact term are provided in supplementary Tables S15–S21. Findings were in similar directions for all “positive” impact terms (*calm, compassionate, edified, enraptured, enlightened, inspired, interested, pleasure*) and all “negative” impact terms (*angry, challenged, upset*).

**Figure 7:**
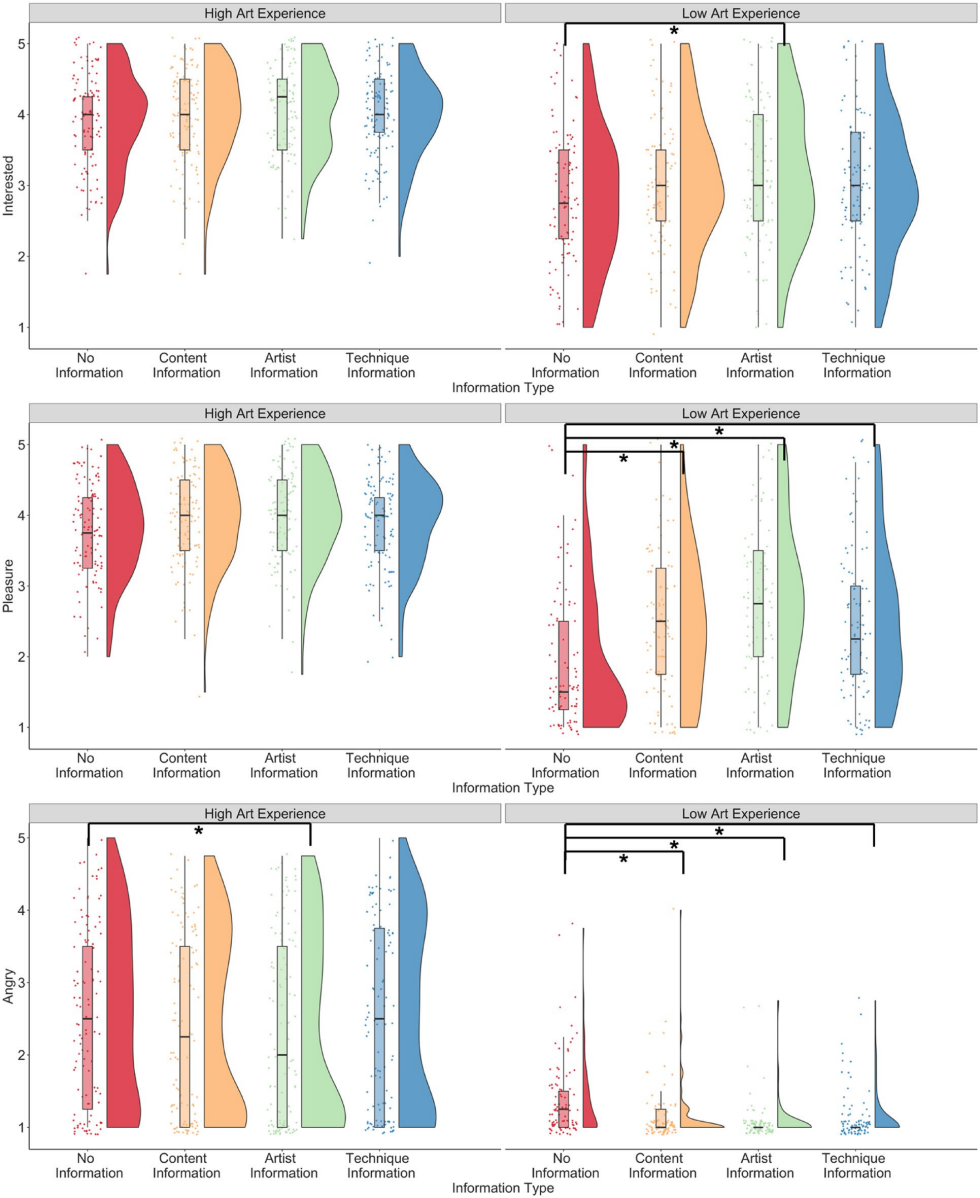
Interest, pleasure, and angry ratings when no information, content information, artist information, and technique information was presented to participants, split by participants with high and low art experience. *p < .05.

For the *art_experience* model, the interaction between content information and art experience predicted ratings of how calm, challenged, inspired, pleasure, and upset the artwork made them feel (all ps < 0.02; Fig. 7). The interaction between artist information and art experience predicted all impact ratings (all ps < 0.05, p = 0.070 for edified ratings), except for how compassionate participants felt on viewing the artwork (p = 0.807). The interaction between technique information and art experience predicted all impact ratings (all ps < 0.007). Art experience predicted all impact ratings positively (p < 0.001), and openness to experience negatively predicted ratings of how angry (p < 0.001) and upset (p < 0.001) participants felt, and positively predicted ratings of interest (p = 0.008). Age positively predicted how edified the artwork made participants feel (p < 0.001). The main effects of content information, artist information, and technique information (compared to no information) predicted all impacts except how compassionate, edified, and enraptured artworks made participants feel (all ps < 0.05; content and technique information did not predict ratings of interest). No other main effects or interactions were significant across models. The model predicted between 19 and 42% of the variance across impact terms.

Posthoc tests revealed that artist information compared to no information showed higher ratings of interest but only for participants with lower art experience (p = 0.013), and not in participants with higher art experience (p = 0.616). Content, artist, and technique information compared to no information showed higher ratings of how much pleasure, and how inspired, enlightened, and calm participants felt, and lower ratings of how angry, upset, and challenged participants felt in participants with lower art experience (all ps < 0.05), whereas only artist information (ps < 0.05) and not content or technique information reduced ratings of how angry and upset participants felt in those with higher art experience (all ps > 0.40).

**Figure 8:**
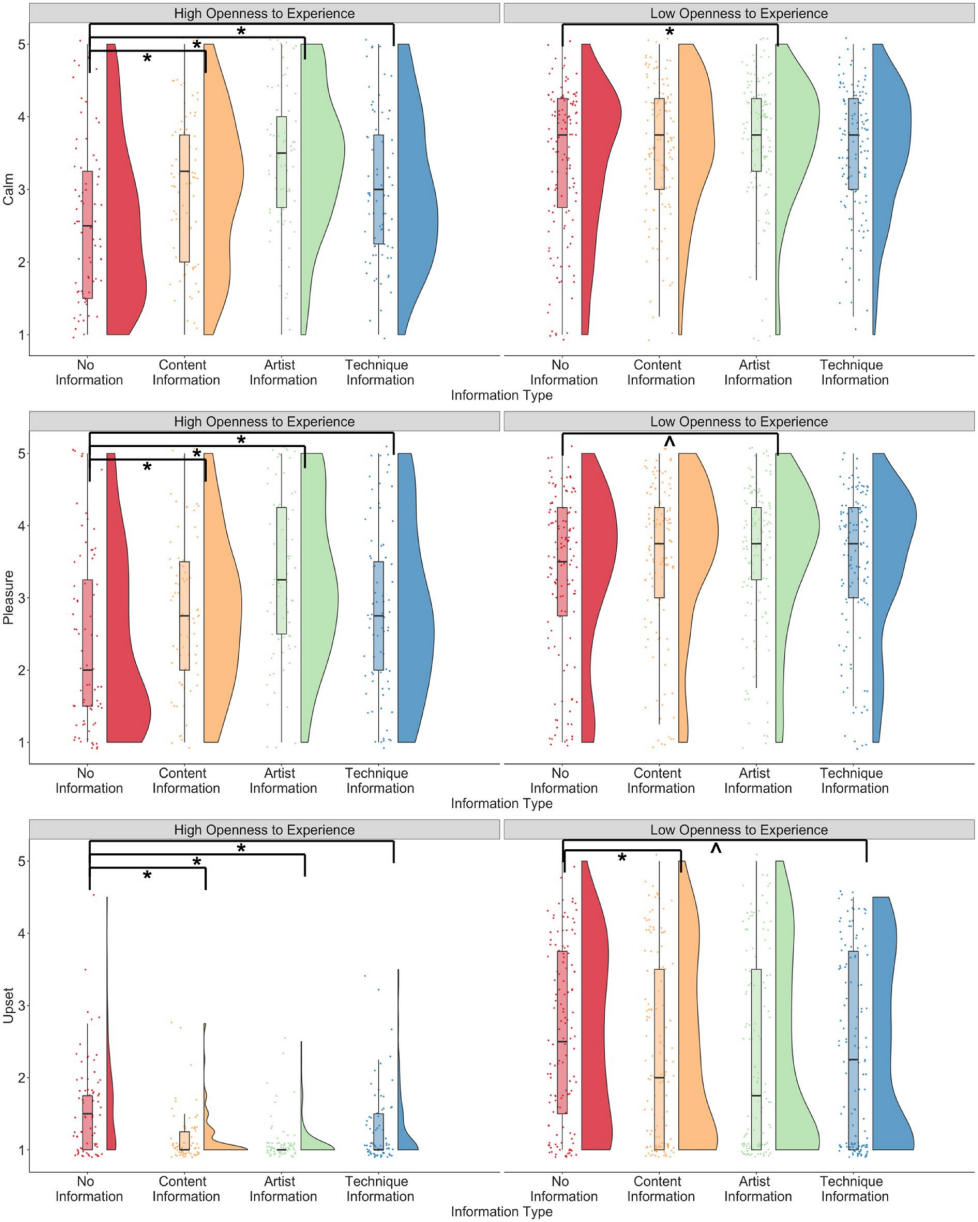
Ratings of pleasure, and how calm and upset participants felt by the artworks when no information, and content, artist, and technique information was presented, split by participants with low and high openness to experience (*p < .05; ^p > .05 and  < .09).

For the *openness_experience* model, the interaction between openness to experience and content information, artist information, and technique information significantly predicted ratings of how much pleasure, how calm and challenged participants felt by the artworks (ps < 0.05; Fig. 8). The interaction between content information and openness to experience also predicted ratings of how edified (p = 0.051) and upset (p = 0.007) participants felt, the interaction between artist information and openness to experience also predicted ratings of how inspired (p < 0.001) and upset (p = 0.029) participants felt, and the interaction between technique information and openness to experience predicted ratings of how inspired participants felt (p < 0.001).

Post hoc tests suggested that participants with higher openness to experience showed higher ratings of calm and pleasure when content, artist, or technique information was presented (all ps < 0.002), whereas participants with lower openness to experience showed higher ratings only when artist information was presented (calm: p = 0.03, pleasure: p = 0.071) and not when content or technique information was presented (all ps > 0.20). Participants with higher openness to experience also showed higher ratings of how inspired they felt when artist or technique information was presented (ps < 0.001), participants with lower openness to experience did not (ps > 0.1).

Participants with higher openness to experience showed lower ratings of how challenged artworks made them feel when artist, content, and technique information was presented (all ps < 0.03) whereas participants with lower openness to experience did not show a difference (all ps > 0.80). Similarly, participants were more upset when no information was presented compared to when artist, technique, or content information was presented in participants with higher openness to experience (all ps < 0.03), whereas participants with lower openness to experience showed lower upset ratings only when artist (p = 0.007) and technique information (p = 0.058) was presented but not when content information was presented (p = 0.129). Main effects of age, art experience, and openness to experience were similar to the *art_experience* model. The model predicted between 20 and 40% of the variance for aesthetic impacts.

For the *art_experience_content* model, the interaction between art experience and content type (whether descriptive or elaborative) predicted ratings of how angry (p = 0.015), upset (p = 0.014), and compassionate (p = 0.002) artworks made participants feel. The main effect of content type was a significant predictor of how compassionate (p < 0.001), edified (p = 0.025), pleasure (marginal, p = 0.08), and upset (p = 0.014) participants felt, with higher ratings of pleasure and how edified the artwork made participants feel for artworks presented with elaborative content compared to descriptive content. Posthoc tests revealed that ratings of how angry, compassionate, and upset participants felt were higher when elaborative content was presented compared to when descriptive content was presented but only for low art experience participants (angry: p = 0.006, upset: p = 0.001, compassionate: p < 0.001) and not high art experience participants (angry: p = 0.570, upset: p = 1.00, compassionate: p = 0.762). Effects of age, art experience, and openness to experience were similar to the *art_experience* model. The model predicted between 18 and 46% of the variance for aesthetic impact terms.

**Figure 9:**
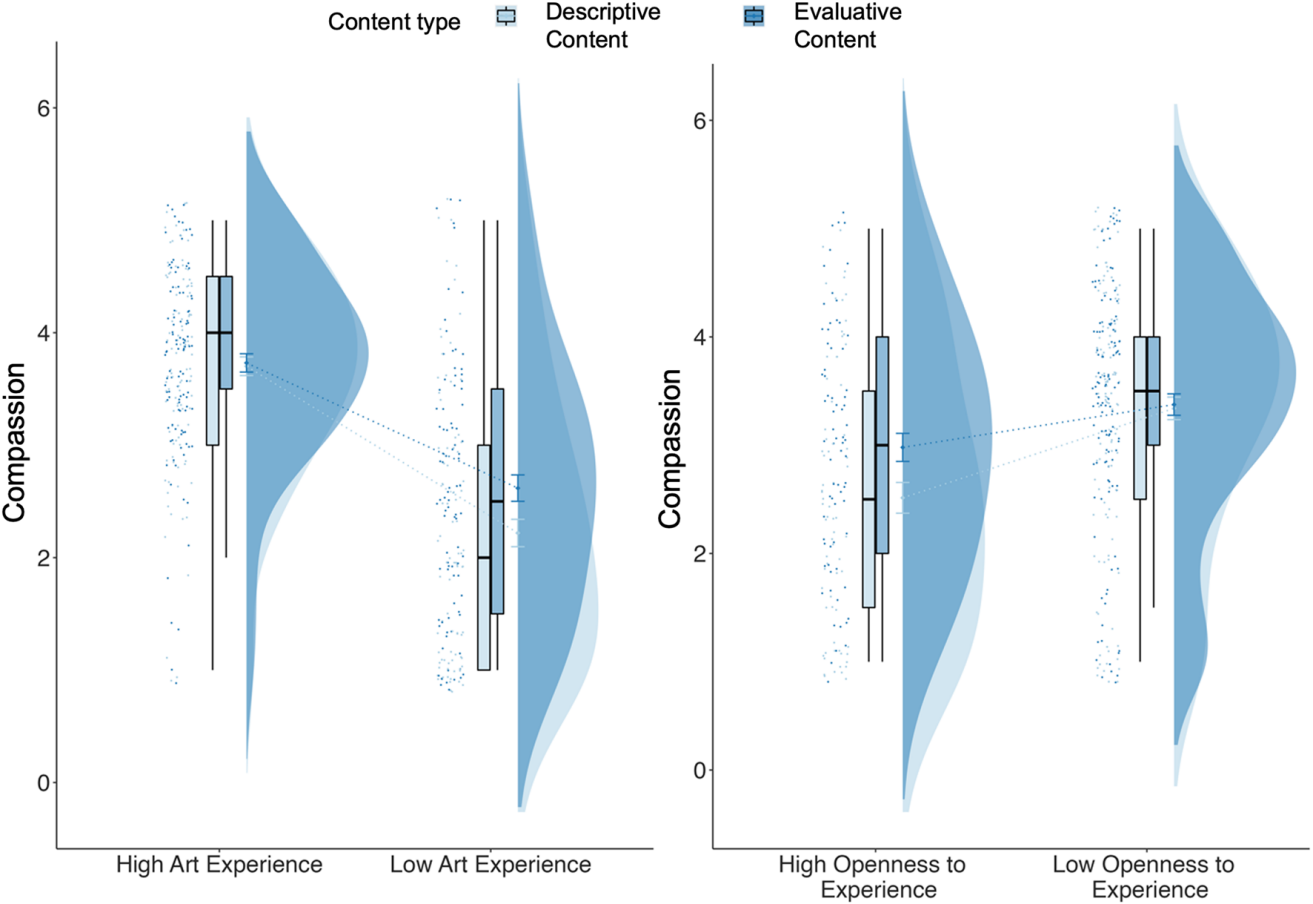
Difference in ratings of how much compassion participants felt when provided with descriptive and elaborative content information, split by participants with low and high art experience, and low and high openness to experience.

For the *openness_experience_content* model, the interaction between content type and openness to experience predicted how compassionate participants felt (p < 0.001). Main effects of age, education, art experience, openness to experience, and content type were similar to the *art_experience_content* model. Post hoc tests revealed that participants with higher openness to experience showed higher ratings of compassion when artworks were presented with elaborative information compared to descriptive information (p < 0.001) but those with lower openness to experience did not show this difference (p = 0.593; Fig. 9).

**Figure 10:**
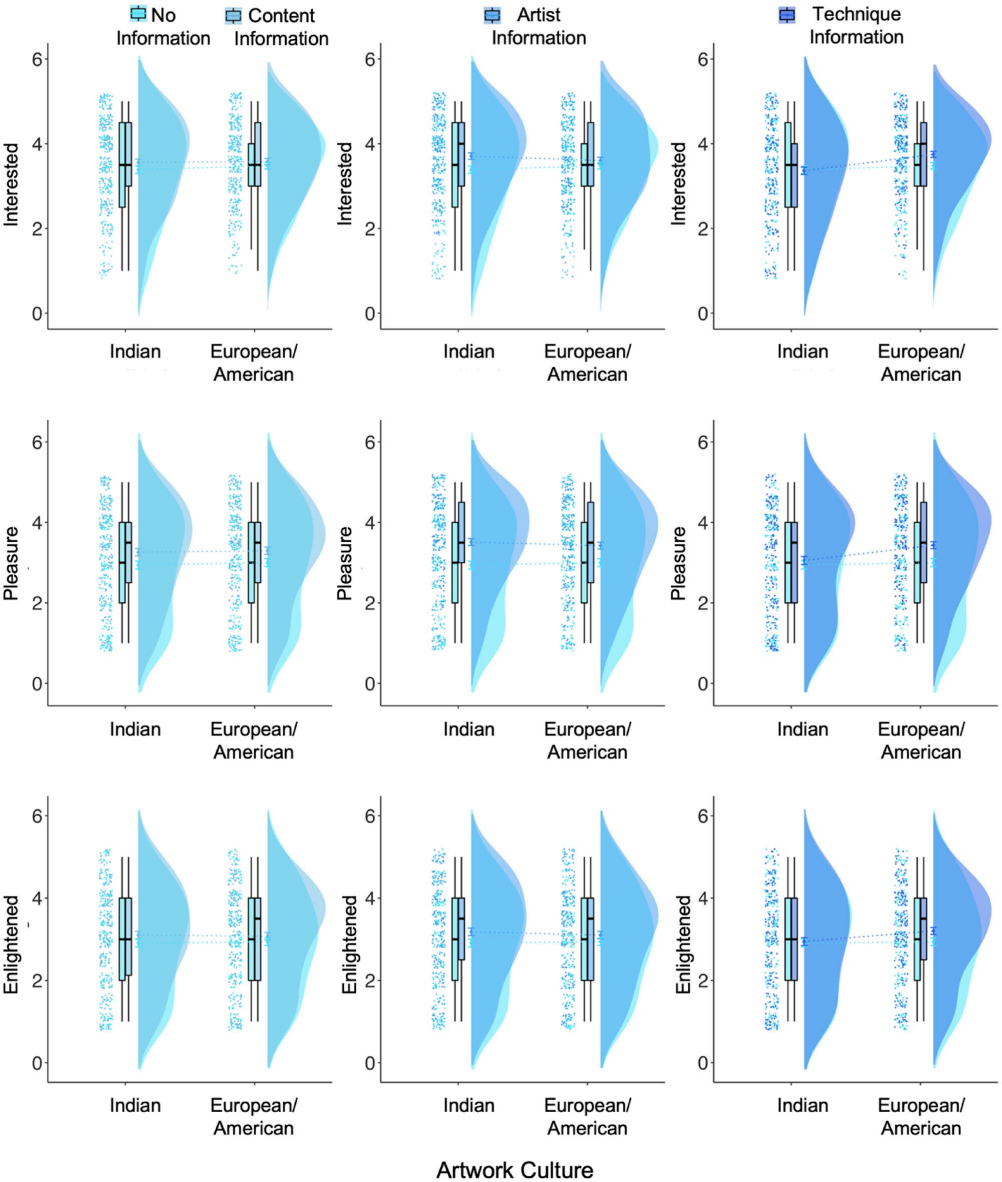
Ratings of how much pleasure, and how interested and enlightened participants felt by Indian and European/American artworks when content information (on the left), artist information (in the centre), and technique information (on the right) are provided to participants compared to no information.

For the *culture* model, the interaction between technique information and artwork culture predicted ratings of how interested (p = 0.023), enlightened (p = 0.029), and how much pleasure (p = 0.028) participants felt when viewing the artwork (Fig. 10). Post hoc tests revealed that artist information and content information compared to no information showed higher ratings of how enlightened and interested participants felt when viewing Indian artworks (enlightened: p = 0.003 for artist, p = 0.085 for content, interest: p = 0.003 for artist) but not European/American artworks (ps > 0.15), whereas technique information compared to no information showed higher ratings of how enlightened participants felt when viewing European/American artworks (enlightened: p = 0.003, interest: p = 0.018) but not Indian artworks (ps > 0.9). Content and artist information showed higher ratings of pleasure for both Indian and European/American artworks (ps < 0.03) whereas technique information only increased pleasure ratings for European/American artworks (p < 0.001) and not Indian artworks (p = 0.738). The model explained 21% to 41% variance for impact ratings.

### Discussion

Experiment 2 investigated whether contextual information about the artist, technique, or content of representational artworks by Indian and European/American artists influenced aesthetic responses of American participants compared to when no information about the artwork was presented. We found that artist, content, and technique information (compared to no information) influenced the aesthetic experience of Indian and European/American representational artworks, albeit in different ways, and was modulated by the art experience and openness to experience of participants.

Specifically, contextual information (whether it was information about the artist, technique, or content) had a higher influence on aesthetic ratings of participants with higher openness to experience, and lower art experience by increasing ratings of liking and beauty and decreasing ratings of complexity. Content information, specifically elaborative content more than descriptive content, reduced ratings of perceived complexity of the artwork. This effect of contextual information was reduced or absent in participants with higher art experience and lower openness to experience.

For aesthetic impacts, our results suggested that overall participants with higher art experience had stronger ratings for aesthetic impacts (whether “positive” or “negative” impact terms) compared to participants with lower art experience. Like liking, beauty, and complexity ratings, aesthetic impact ratings were also influenced by contextual information more for participants with lower art experience and higher openness to experience. Overall, “positive” impact ratings were higher and “negative” impact ratings were lower when contextual information was presented compared to when no information was presented.

Although effects were quite small, contextual information about the artist and the content (but not technique) decreased the ingroup bias (the tendency of American participants to prefer European/American artworks more than Indian artworks), suggesting that contextual information may influence aesthetic ratings of artworks belonging to another culture more than artworks belonging to one’s own culture. Perceived complexity of the artworks was reduced more so for Indian artworks than European/American artworks when technique and content information was provided to the participants compared to no information.

## General discussion

In the current study, we investigated how contextual information influences the aesthetic appreciation of artworks. In Experiment 1, we explored whether contextual information such as artist or technique information influenced aesthetic judgments of abstract artworks by Jackson Pollock. We found a small effect of contextual information on aesthetic ratings which was modulated by art experience and openness to experience. Specifically, the combination of artist and technique information (but not artist or technique information in isolation) increased liking and interest, and mostly for participants with little art experience, and those who were more open to experience. Experiment 2 investigated whether contextual information about the artist, technique, or content of representational artworks by Indian and European/American artists influenced aesthetic responses of Northern American participants compared to when no information about the artwork was presented. We found that artist, content, and technique information (compared to no information) influenced the aesthetic experience of Indian and European/American representational artworks, and was modulated by artwork culture, and by participants’ art experience and openness to experience. Below we evaluate each of our research questions from Experiments 1 and 2 in more detail.

### Does contextual information influence aesthetic ratings?

Our findings from Experiments 1 and 2 are in line with previous research that suggests that contextual information influences aesthetic ratings (e.g., 7, 21), but is modulated by art experience and openness to experience of participants. The aesthetic appreciation of both abstract and representational artworks in our study was impacted by contextualizing information about the artwork, although the effects were smaller for abstract artworks by Jackson Pollock. One explanation for the findings from Experiment 1 could be the effect of mere exposure on participants. That is, participants first viewed paintings with no information (block 1) followed by one type of information (block 2), and then both types of information (block 3). Thus, it is possible that participants’ ratings in block 3 were impacted by exposure to the stimuli in blocks 1 and 2 as opposed to the contextual information presented in block 2 and 3. However, this explanation seems unlikely because in Experiment 2, the order in which different types of information were presented were randomized across participants. We still found an effect of contextual information compared to no information. Therefore, we are confident that findings from both Experiments 1 and 2 reflect an effect of contextual information, and cannot be easily explained by a mere exposure effect.

An effect of contextualizing information is consistent with fluency theory^23^ or the ‘effort after meaning’ theory^41^. The fluency theory suggests that ease of processing of artworks positively impacts its aesthetic appreciation. Thus, contextualizing information that increases processing fluency of artworks may increase its aesthetic appreciation. Similarly, the ‘effort after meaning’ theory suggests that understanding the meaning behind artworks by the viewers (that might be aided by contextualizing information), leads to higher aesthetic evaluation of the artwork. Thus, contextual information in both Experiments 1 and 2 might have increased processing fluency and understanding of the artworks, enhancing their aesthetic appreciation as suggested by higher ratings of liking, beauty, and interest.

However, participants’ ratings of complexity were not reduced when given artist or technique-related information for either abstract or representational artworks. Only content-related information reduced complexity of representational artworks compared to when no information was provided. This finding suggests that processing images more easily (as measured by decreased ratings of complexity) might only be facilitated by content-related information but not artist or technique related information. No content-related information was provided for abstract artworks, and artist and technique related information was provided before a subset of ten or eleven artworks as opposed to each individual artwork. The smaller effects of artist and technique information (and no effect of artist or technique information in isolation) might be accounted for by an increased understanding about the style and intent of the artist, and not an increased processing fluency or understanding of each individual artwork.

Overall, our findings suggest that contextual information impacts aesthetic evaluations of abstract and representational artworks. Future work can further explore how different types of information i.e., information about the artwork’s content, its artist, or the artist’s technique influence aesthetic evaluations through different routes – that is, it is possible that content information might increase processing fluency more that artist/technique information, whereas artist and technique information may enhance the understanding and/or meaningfulness of the artwork more than content information.

### Does the type of content information (descriptive or elaborative) influence aesthetic ratings?

In Experiment 2, participants were provided with two kinds of content-related information: descriptive content information was redundant with what the viewer could observe in the artwork and did not reveal any deeper meaning about the artwork, whereas elaborative information provided information that gave the viewer a richer interpretation of the artwork as informed by the literature about the artwork, and the artist’s intent (see Box 2 for examples). In contrast to previous studies that found an effect of elaborative content information compared to descriptive content information on aesthetic ratings^7,17,21^, we did not see any differences on aesthetic ratings and impacts (except ratings of compassion). One explanation for this discrepancy is that previous studies^7,17^ that found a difference between descriptive and elaborative information used titles instead of detailed information. Thus, detailed descriptive content information about what can be directly perceived in the artwork could lead to easier processing of the artwork than elaborative information that provides a deeper understanding of the artwork, thus increasing aesthetic ratings for both types of content albeit in different ways. Our results follow those of Krauss and colleagues^22^ who also did not find differences in ratings of liking between detailed elaborative and descriptive information. However, we also found lower complexity ratings and higher ratings of how much compassion participants felt after participants were provided with elaborative information compared to descriptive information. Thus, complexity of the artwork may have been reduced by elaborative information more than descriptive information, allowing participants to further deconstruct the artwork, and make a richer interpretation^40^. Thus, the effort of making sense of the artwork or understanding the artist’s message behind the artwork might increase how much compassion participants felt for the artworks (but not how much they liked it or were otherwise impacted by it).

### Does art experience and openness to experience modulate the effect of contextual information on aesthetic ratings?

A long-standing tradition of studies in empirical aesthetics has shown that art experience modulates aesthetic appreciation^42^. We found increased effects of contextual information for individuals with lower art experience and higher openness to experience. Leder et al.^43^ suggest that people with more art experience and knowledge might perceive artworks differently that art-naïve individuals. As expertise in art increases, individuals engage with the style of artworks, rather than other information associated with it. Thus, in individuals with more art experience, processing fluency might already be higher, and a deeper understanding of the artwork may already be attained by its stylistic information and by existing knowledge, thus making these people less susceptible to contextual information. Similarly, as those higher on openness to experience seek novelty in artworks, novel information about the artwork might have a bigger impact on their aesthetic evaluations^29^.

An alternative but not necessarily contradictory theory – the uncertainty identity theory—can also explain the modulatory effect of art experience. The uncertainty-identity hypothesis in the context of art appreciation suggests that people who feel more uncertain about judging art may resort to using a social identity heuristic to judge art^10,44^. Thus, people with less art experience may feel unsure about their judgements and resort to using contextual information as a heuristic to evaluate art more than those who feel more certain about their judgments i.e., participants higher in art experience. This possibility is further supported by previous research by Cleeremans and colleagues^24^ who found that art-naïve participants but not art experts used the name of the artist as a heuristic to evaluate a work of art.

In line with previous work, aesthetic ratings of liking and beauty as well as aesthetic impacts were higher overall for participants with more art experience compared to less art experience. Responses for how an artwork made participants think or feel (whether positive or negative) were higher overall for those with more art experience. Therefore, ceiling effects in participants with higher art experience may explain why they did not show an effect of contextual information. For negative impact ratings of how angry or upset participants felt, more variability in responses were found for individuals with more art experience. Ratings of anger and how upset participants felt were lower when artist and content-related information was presented compared to when no information was presented not just in participants with low experience but also in those with higher experience. Thus, future work could begin to investigate cognitive and neural mechanisms of how different types of contextual information might impact aesthetic evaluations by including a more diverse set of artworks that might elicit a more variable response across participants to avoid ceiling effects.

### Does the cultural origin of the artwork modulate the effect of contextual information on aesthetic ratings?

Our findings on the effect of artwork culture might also be explained by the uncertainty-identity hypothesis. Northern American participants may be more unsure about their aesthetic evaluations of unfamiliar artworks (here Indian artworks compared to European/American artworks). Thus, they may rely on contextual information more for Indian than European/American artworks. In support of this possibility, in the current study, we found that artist and content information increased aesthetic ratings of Indian artworks more than European/American artworks, but technique information increased aesthetic ratings for European/American artworks. Content and artist information may serve as heuristics to guide aesthetic evaluations of unfamiliar artworks as they may be directly relevant to the uncertainty that viewers face –whereas techniques or styles may not differ as much across cultures. Thus, additional information about the ambiguous characteristics of an artwork increases aesthetic ratings for unfamiliar artworks. Technique and content information also decreased perceived complexity of Indian artworks. It is possible that Northern American participants may find the content of unfamiliar (Indian) artworks more complex and ambiguous and both information about the content and technique reduces perceived complexity. Future investigations could test this proposal directly by manipulating both the cultural origin of the artwork as well as the participants and use distinct types of contextual information. Indeed, the nature and depth of information provided about different kinds of artworks to viewers and the impact of such information on their attention and engagement with the artworks warrants further investigation.

### Do people simulate the artist’s actions when viewing abstract paintings if contextual information about the artist’s painting technique is provided?

In the current work, we also tested whether providing technique-related information about Jackson Pollock’s action painting style modulated aesthetic ratings of artworks higher and lower in implied motion. The embodied simulation account suggests that observers simulate the movements and experiences of the artwork’s subject or the artists’ actions; ^25^). In Pollock’s abstract artworks, information about the artist’s action painting style would allow viewers to better simulate the artist’s actions, thereby modulating aesthetic ratings, especially in artworks with higher implied motion. Indeed, Ticini et al.^45^ found that when participants were trained to make pointillist movements with a paintbrush, liking of pointillist paintings was enhanced when the paintings were primed with congruent images of hand grips for making pointillist movements. However, we did not find an effect of technique-information alone on aesthetic ratings. Instead, information about both the artist and the technique increased liking for artworks lower in motion. Our findings therefore do not support a straightforward embodied simulation account of aesthetics^30^.

### Implications for the neurocognitive mechanisms underlying aesthetic appreciation

According to the aesthetic triad model, sensory-motor, emotion-valuation, and knowledge-meaning systems interact to bring about aesthetic experiences^6^. Taken together, our findings provide further evidence that knowledge systems modulate the perception and appreciation of visual art, and this modulation plays out in different ways depending on the type of contextual information. It is plausible that artist-related contextual information modulates knowledge-meaning systems but has little impact on visual processing directly. Prior neuroimaging studies providing a semantic context before an aesthetic judgement did not use content-related information but manipulated the source of the artwork (whether it was from an art gallery or computer-generated, or from a museum or adult education center) and found activation in the emotion valuation and knowledge systems, but not in the sensory-motor systems^46^. However, content-related information may change and reduce perceived complexity of images, along with a modulation in semantic processing. Future research using neuroimaging techniques can start to explore how knowledge systems in the brain modulate the perception and appreciation of artworks, and how different types of context might influence the interplay between sensory-motor, emotion-valuation, and knowledge-meaning systems.

### Implications for aesthetics education and museum curation

Along with theories of empirical aesthetics, and the neurocognitive underpinnings of art perception and appreciation, and its modulation by contextual information, the current findings also have important implications for aesthetics education and museum curation. Two streams of thought are evident in art education – a focus on art historical context and background, or a personal connection between the artwork and one’s own life experiences^47^. In the Barnes Foundation in Philadelphia, for instance, thousands of different works are arranged without labels or categories and the intent is for viewers to think for themselves and make connections without any contextual information about the artwork (barnesfoundation.org/seeingthebarnes). Thus, our findings are relevant to art education by identifying contextual factors that influence aesthetic experiences. Given our findings, it seems crucial to consider the type of artwork, the type of contextual information provided, its potential to enhance aesthetic experience, and the curatorial background of the museum or exhibition^48^, as well as individual differences of viewers. Artworks that are unfamiliar to its viewers might require more contextual information to have an impact on the viewer, and in some contexts, also lower viewers’ biases or prejudices against artworks or artists originating from an out-group^9,10^.

## Supplementary Information

Supplementary Information.

## Data Availability

Following open science initiatives^49^, all raw data and code are available online for other researchers to pursue alternative questions of interest (https://osf.io/kus4c/).
