# Supplementary material for: The impact of contextual information on aesthetic engagement of artworks
Source: Sci Rep. 2023 Mar 15;13:4273. doi: 10.1038/s41598-023-30768-9 (PMC10017684; doi:10.1038/s41598-023-30768-9)
Supplement: Supplementary file 1 — Supplementary Information. [file 41598_2023_30768_MOESM1_ESM.docx]

**Supplementary Materials**

**Put it into context:**

**The impact of contextual information on aesthetic engagement**

Kohinoor M. Darda^1^, Anjan Chatterjee^1^

^1^Penn Center for Neuroaesthetics, University of Pennsylvania, Philadelphia, PA, USA

Table S1. Participant demographics for Experiment 1 for participants who did the artist information block first, and the participants who did the technique information block first.

|  | **art_info_first** | | | **tech_info_first** | | |
| --- | --- | --- | --- | --- | --- | --- |
| **Variable** | **N** | **Mean** | **SD** | **N** | **Mean** | **SD** |
| Age | 110 | 36.65 | 10.09 | 104 | 37.26 | 10.41 |
| Gender | 110 |  |  | 104 |  |  |
| ... 1.Men | 65 | 59% |  | 46 | 44% |  |
| ... 2.Women | 45 | 41% |  | 57 | 55% |  |
| ... Other/Did not specify | 0 | 0% |  | 1 | 1% |  |
| Time taken to complete  The survey (in mins) | 110 | 19.60 | 11.65 | 104 | 22.68 | 14.42 |
| Education (no. of years) | 110 | 15.33 | 2.00 | 104 | 15.21 | 2.48 |
| Race | 110 |  |  | 104 |  |  |
| ... American Indian/  Alaska Native | 1 | 0.9% |  | 0 | 0% |  |
| ... Asian | 1 | 0.9% |  | 1 | 1% |  |
| ... Black/  African American | 8 | 7% |  | 6 | 6% |  |
| ... Unknown/  Did not specify/ Other | 0 | 0% |  | 1 | 1% |  |
| ... White | 100 | 91% |  | 96 | 92% |  |
| Art Experience | 108 | 24.46 | 13.26 | 104 | 28.63 | 12.74 |
| Openness to Experience | 110 | 128.41 | 15.84 | 104 | 126.28 | 13.20 |

Table S2. Beta estimates, confidence intervals, p-values for *info_type* model.

|  | **liking** | | | | **interest** | | | | **complexity** | | | |
| --- | --- | --- | --- | --- | --- | --- | --- | --- | --- | --- | --- | --- |
| *Predictors* | *Estimates* | *CI* | *Statistic* | *p* | *Estimates* | *CI* | *Statistic* | *p* | *Estimates* | *CI* | *Statistic* | *p* |
| Intercept | 3.59 | 3.49 – 3.69 | 70.87 | **<0.001** | 3.64 | 3.54 – 3.74 | 73.24 | **<0.001** | 3.67 | 3.56 – 3.77 | 69.17 | **<0.001** |
| Artist Information Only | 0.01 | -0.09 – 0.11 | 0.13 | 0.894 | 0.03 | -0.07 – 0.13 | 0.62 | 0.532 | 0.04 | -0.06 – 0.14 | 0.78 | 0.437 |
| Technique Information Only | 0.04 | -0.06 – 0.14 | 0.80 | 0.426 | 0.02 | -0.08 – 0.13 | 0.46 | 0.646 | -0.00 | -0.10 – 0.10 | -0.06 | 0.953 |
| Both Types of /n Information | 0.08 | -0.00 – 0.16 | 1.86 | 0.062 | 0.06 | -0.02 – 0.15 | 1.43 | 0.153 | 0.05 | -0.03 – 0.13 | 1.23 | 0.220 |
| Art Experience | 0.58 | 0.49 – 0.67 | 12.86 | **<0.001** | 0.57 | 0.49 – 0.66 | 13.36 | **<0.001** | 0.39 | 0.29 – 0.48 | 7.94 | **<0.001** |
| Age | -0.00 | -0.08 – 0.08 | -0.04 | 0.972 | -0.00 | -0.08 – 0.07 | -0.01 | 0.992 | -0.04 | -0.13 – 0.05 | -0.92 | 0.357 |
| Education | -0.00 | -0.08 – 0.08 | -0.01 | 0.992 | -0.01 | -0.08 – 0.06 | -0.29 | 0.775 | 0.05 | -0.03 – 0.14 | 1.28 | 0.200 |
| Openness to Experience | 0.05 | -0.04 – 0.13 | 1.06 | 0.287 | 0.08 | -0.00 – 0.16 | 1.87 | 0.062 | 0.12 | 0.03 – 0.21 | 2.56 | **0.011** |
| **Random Effects** | | | | | | | | | | | | |
| σ^2^ | 0.60 | | | | 0.59 | | | | 0.57 | | | |
| τ_00_ | 0.31 _sid_ | | | | 0.28 _sid_ | | | | 0.36 _sid_ | | | |
|  | 0.01 _itemno_ | | | | 0.01 _itemno_ | | | | 0.01 _itemno_ | | | |
| ICC | 0.34 | | | | 0.33 | | | | 0.39 | | | |
| N | 212 _sid_ | | | | 212 _sid_ | | | | 212 _sid_ | | | |
|  | 31 _itemno_ | | | | 31 _itemno_ | | | | 31 _itemno_ | | | |
| Observations | 6569 | | | | 6565 | | | | 6568 | | | |
| Marginal R^2^ / Conditional R^2^ | 0.256 / 0.512 | | | | 0.250 / 0.494 | | | | 0.124 / 0.469 | | | |

Table S3. Beta values, confidence intervals, p-values for *art_experience* model.

|  | **liking** | | | | **interest** | | | | **complexity** | | | |
| --- | --- | --- | --- | --- | --- | --- | --- | --- | --- | --- | --- | --- |
| *Predictors* | *Estimates* | *CI* | *Statistic* | *p* | *Estimates* | *CI* | *Statistic* | *p* | *Estimates* | *CI* | *Statistic* | *p* |
| Intercept | 3.53 | 3.42 – 3.64 | 61.90 | **<0.001** | 3.58 | 3.47 – 3.69 | 64.29 | **<0.001** | 3.63 | 3.52 – 3.74 | 64.71 | **<0.001** |
| Artist Information Only | 0.00 | -0.10 – 0.10 | 0.03 | 0.974 | 0.03 | -0.08 – 0.13 | 0.49 | 0.622 | 0.03 | -0.06 – 0.13 | 0.68 | 0.498 |
| Technique Information Only | 0.05 | -0.05 – 0.16 | 0.95 | 0.342 | 0.04 | -0.07 – 0.14 | 0.67 | 0.503 | 0.01 | -0.10 – 0.11 | 0.11 | 0.910 |
| Both Types of Information | 0.09 | 0.01 – 0.17 | 2.15 | **0.032** | 0.08 | -0.01 – 0.16 | 1.75 | 0.080 | 0.06 | -0.02 – 0.14 | 1.45 | 0.148 |
| Art Experience | 0.83 | 0.62 – 1.03 | 7.91 | **<0.001** | 0.90 | 0.71 – 1.10 | 9.11 | **<0.001** | 0.55 | 0.35 – 0.75 | 5.36 | **<0.001** |
| Age | -0.04 | -0.13 – 0.05 | -0.89 | 0.371 | -0.04 | -0.13 – 0.05 | -0.85 | 0.393 | -0.07 | -0.16 – 0.02 | -1.43 | 0.153 |
| Education | 0.04 | -0.05 – 0.13 | 0.85 | 0.394 | 0.03 | -0.06 – 0.11 | 0.63 | 0.530 | 0.08 | -0.01 – 0.17 | 1.80 | 0.072 |
| Openness to Experience | -0.09 | -0.19 – 0.01 | -1.84 | 0.066 | -0.05 | -0.14 – 0.04 | -1.05 | 0.294 | 0.03 | -0.07 – 0.12 | 0.58 | 0.565 |
| Artist Information x Art Experience | -0.10 | -0.22 – 0.02 | -1.65 | 0.100 | -0.17 | -0.28 – -0.05 | -2.78 | **0.005** | -0.08 | -0.19 – 0.03 | -1.37 | 0.170 |
| Technique Information x Art Experience | -0.04 | -0.16 – 0.09 | -0.53 | 0.595 | -0.05 | -0.18 – 0.08 | -0.75 | 0.456 | -0.03 | -0.16 – 0.09 | -0.54 | 0.588 |
| Both Types of Information x Artist Experience | -0.16 | -0.25 – -0.07 | -3.38 | **0.001** | -0.20 | -0.30 – -0.11 | -4.33 | **<0.001** | -0.11 | -0.20 – -0.02 | -2.44 | **0.015** |
| **Random Effects** | | | | | | | | | | | | |
| σ^2^ | 0.60 | | | | 0.59 | | | | 0.57 | | | |
| τ_00_ | 0.44 _sid_ | | | | 0.39 _sid_ | | | | 0.42 _sid_ | | | |
|  | 0.01 _itemno_ | | | | 0.01 _itemno_ | | | | 0.01 _itemno_ | | | |
| ICC | 0.43 | | | | 0.41 | | | | 0.43 | | | |
| N | 214 _sid_ | | | | 214 _sid_ | | | | 214 _sid_ | | | |
|  | 31 _itemno_ | | | | 31 _itemno_ | | | | 31 _itemno_ | | | |
| Observations | 6631 | | | | 6627 | | | | 6630 | | | |
| Marginal R^2^ / Conditional R^2^ | 0.147 / 0.514 | | | | 0.154 / 0.497 | | | | 0.066 / 0.469 | | | |

Table S4. Beta values, confidence intervals, p-values for the *openness_experience* model.

|  | **liking** | | | | **interest** | | | | **complexity** | | | |
| --- | --- | --- | --- | --- | --- | --- | --- | --- | --- | --- | --- | --- |
| *Predictors* | *Estimates* | *CI* | *Statistic* | *p* | *Estimates* | *CI* | *Statistic* | *p* | *Estimates* | *CI* | *Statistic* | *p* |
| Intercept | 3.59 | 3.49 – 3.70 | 67.23 | **<0.001** | 3.65 | 3.55 – 3.76 | 69.80 | **<0.001** | 3.68 | 3.57 – 3.79 | 65.14 | **<0.001** |
| Artist Information Only | 0.01 | -0.09 – 0.11 | 0.24 | 0.807 | 0.04 | -0.06 – 0.15 | 0.82 | 0.414 | 0.04 | -0.06 – 0.14 | 0.78 | 0.434 |
| Technique Information Only | 0.05 | -0.06 – 0.15 | 0.89 | 0.372 | 0.04 | -0.06 – 0.15 | 0.81 | 0.420 | -0.03 | -0.13 – 0.08 | -0.52 | 0.602 |
| Both Types of Information | 0.11 | 0.03 – 0.19 | 2.54 | **0.011** | 0.08 | -0.00 – 0.17 | 1.92 | 0.055 | 0.06 | -0.02 – 0.15 | 1.48 | 0.140 |
| Openness to Experience | 0.02 | -0.17 – 0.22 | 0.23 | 0.815 | 0.07 | -0.11 – 0.26 | 0.79 | 0.432 | 0.08 | -0.13 – 0.29 | 0.75 | 0.453 |
| Age | -0.00 | -0.08 – 0.08 | -0.07 | 0.944 | -0.00 | -0.08 – 0.07 | -0.07 | 0.943 | -0.04 | -0.13 – 0.05 | -0.93 | 0.353 |
| Education | -0.00 | -0.08 – 0.08 | -0.04 | 0.968 | -0.01 | -0.09 – 0.06 | -0.33 | 0.738 | 0.05 | -0.03 – 0.14 | 1.26 | 0.206 |
| Art Experience | 0.58 | 0.49 – 0.67 | 12.79 | **<0.001** | 0.57 | 0.48 – 0.65 | 13.20 | **<0.001** | 0.35 | 0.25 – 0.44 | 7.03 | **<0.001** |
| Artist Information x Openness to Experience | 0.04 | -0.08 – 0.17 | 0.66 | 0.508 | 0.07 | -0.05 – 0.19 | 1.14 | 0.254 | 0.01 | -0.11 – 0.13 | 0.12 | 0.905 |
| Technique Information x Openness to Experience | 0.03 | -0.10 – 0.17 | 0.47 | 0.636 | 0.10 | -0.03 – 0.23 | 1.52 | 0.128 | -0.13 | -0.26 – -0.00 | -1.96 | **0.050** |
| Both Types of Information x Openness to Experience | 0.18 | 0.08 – 0.28 | 3.64 | **<0.001** | 0.13 | 0.04 – 0.23 | 2.71 | **0.007** | 0.07 | -0.03 – 0.16 | 1.41 | 0.157 |
| **Random Effects** | | | | | | | | | | | | |
| σ^2^ | 0.60 | | | | 0.59 | | | | 0.57 | | | |
| τ_00_ | 0.31 _sid_ | | | | 0.28 _sid_ | | | | 0.37 _sid_ | | | |
|  | 0.01 _itemno_ | | | | 0.01 _itemno_ | | | | 0.01 _itemno_ | | | |
| ICC | 0.34 | | | | 0.33 | | | | 0.40 | | | |
| N | 212 _sid_ | | | | 212 _sid_ | | | | 212 _sid_ | | | |
|  | 31 _itemno_ | | | | 31 _itemno_ | | | | 31 _itemno_ | | | |
| Observations | 6569 | | | | 6565 | | | | 6568 | | | |
| Marginal R^2^ / Conditional R^2^ | 0.257 / 0.514 | | | | 0.250 / 0.495 | | | | 0.115 / 0.470 | | | |

Table S5. Beta values, confidence intervals, p-values for the *motion* model.

|  | **liking** | | | | **interest** | | | | **complexity** | | | |
| --- | --- | --- | --- | --- | --- | --- | --- | --- | --- | --- | --- | --- |
| *Predictors* | *Estimates* | *CI* | *Statistic* | *p* | *Estimates* | *CI* | *Statistic* | *p* | *Estimates* | *CI* | *Statistic* | *p* |
| Intercept | 3.59 | 3.49 – 3.69 | 73.87 | **<0.001** | 3.64 | 3.54 – 3.74 | 73.04 | **<0.001** | 3.67 | 3.56 – 3.77 | 70.19 | **<0.001** |
| Artist Information Only | 0.02 | -0.07 – 0.11 | 0.40 | 0.690 | 0.03 | -0.07 – 0.14 | 0.64 | 0.522 | 0.04 | -0.06 – 0.13 | 0.75 | 0.456 |
| Technique Information Only | 0.03 | -0.07 – 0.12 | 0.50 | 0.617 | 0.04 | -0.07 – 0.15 | 0.70 | 0.483 | -0.01 | -0.11 – 0.09 | -0.22 | 0.829 |
| Both Types of Information | 0.09 | 0.02 – 0.17 | 2.37 | **0.018** | 0.06 | -0.03 – 0.15 | 1.39 | 0.165 | 0.05 | -0.03 – 0.13 | 1.15 | 0.249 |
| Motion | -0.02 | -0.14 – 0.10 | -0.28 | 0.783 | -0.01 | -0.15 – 0.12 | -0.16 | 0.872 | 0.08 | -0.05 – 0.20 | 1.24 | 0.216 |
| Age | -0.00 | -0.08 – 0.08 | -0.04 | 0.970 | -0.00 | -0.08 – 0.07 | -0.01 | 0.992 | -0.04 | -0.12 – 0.05 | -0.92 | 0.357 |
| Education | -0.00 | -0.08 – 0.08 | -0.01 | 0.994 | -0.01 | -0.08 – 0.06 | -0.29 | 0.775 | 0.05 | -0.03 – 0.14 | 1.28 | 0.200 |
| Art Experience | 0.58 | 0.49 – 0.67 | 12.86 | **<0.001** | 0.57 | 0.49 – 0.66 | 13.36 | **<0.001** | 0.39 | 0.29 – 0.48 | 7.94 | **<0.001** |
| Openness to Experience | 0.05 | -0.04 – 0.13 | 1.07 | 0.287 | 0.08 | -0.00 – 0.16 | 1.87 | 0.062 | 0.12 | 0.03 – 0.21 | 2.56 | **0.011** |
| Artist Information x Motion | -0.15 | -0.32 – 0.02 | -1.73 | 0.085 | -0.02 | -0.21 – 0.17 | -0.19 | 0.848 | -0.02 | -0.20 – 0.15 | -0.28 | 0.782 |
| Technique Information x Motion | 0.07 | -0.11 – 0.25 | 0.79 | 0.427 | -0.06 | -0.26 – 0.14 | -0.58 | 0.561 | -0.04 | -0.22 – 0.15 | -0.39 | 0.699 |
| Both Types of Information x Motion | -0.10 | -0.25 – 0.06 | -1.23 | 0.218 | 0.02 | -0.15 – 0.19 | 0.20 | 0.843 | -0.04 | -0.21 – 0.12 | -0.55 | 0.585 |
| **Random Effects** | | | | | | | | | | | | |
| σ^2^ | 0.60 | | | | 0.59 | | | | 0.57 | | | |
| τ_00_ | 0.31 _sid_ | | | | 0.28 _sid_ | | | | 0.36 _sid_ | | | |
|  | 0.01 _itemno_ | | | | 0.01 _itemno_ | | | | 0.01 _itemno_ | | | |
| ICC | 0.34 | | | | 0.33 | | | | 0.39 | | | |
| N | 212 _sid_ | | | | 212 _sid_ | | | | 212 _sid_ | | | |
|  | 31 _itemno_ | | | | 31 _itemno_ | | | | 31 _itemno_ | | | |
| Observations | 6569 | | | | 6565 | | | | 6568 | | | |
| Marginal R^2^ / Conditional R^2^ | 0.258 / 0.513 | | | | 0.250 / 0.494 | | | | 0.125 / 0.469 | | | |

Table S6. Participant demographics for Experiment 2.

| **Variable** | **N** | **Mean** | **SD** |
| --- | --- | --- | --- |
| Age | 198 | 39.41 | 11.28 |
| Gender | 198 |  |  |
| ... 1.Men | 96 | 48% |  |
| ... 2.Women | 98 | 49% |  |
| ... 3.Non-Binary | 1 | 0.5% |  |
| ... Other/Did not specify | 3 | 2% |  |
| Time taken to complete the survey (in mins) | 198 | 31.50 | 18.56 |
| Education (no. of years) | 198 | 15.23 | 2.34 |
| Race | 198 |  |  |
| ... American Indian/Alaska Native | 3 | 2% |  |
| ... Asian | 10 | 5% |  |
| ... Black/African American | 16 | 8% |  |
| ... Unknown/Did not specify/other | 5 | 3% |  |
| ... White | 164 | 83% |  |
| Art Experience | 198 | 19.92 | 14.23 |
| Openness to Experience | 196 | 132.32 | 18.71 |

Table S7. Beta values, confidence intervals, p-values for the *info_type* model.

|  | **liking** | | | | **beauty** | | | | **complexity** | | | |
| --- | --- | --- | --- | --- | --- | --- | --- | --- | --- | --- | --- | --- |
| *Predictors* | *Estimates* | *CI* | *Statistic* | *p* | *Estimates* | *CI* | *Statistic* | *p* | *Estimates* | *CI* | *Statistic* | *p* |
| Intercept | 3.44 | 3.26 – 3.61 | 38.32 | **<0.001** | 3.56 | 3.39 – 3.73 | 40.70 | **<0.001** | 3.69 | 3.53 – 3.86 | 44.48 | **<0.001** |
| Content Information | 0.18 | -0.03 – 0.40 | 1.67 | 0.096 | 0.07 | -0.14 – 0.28 | 0.64 | 0.525 | -0.18 | -0.36 – -0.01 | -2.09 | **0.037** |
| Artist Information | 0.35 | 0.13 – 0.56 | 3.13 | **0.002** | 0.32 | 0.11 – 0.53 | 2.95 | **0.003** | -0.00 | -0.17 – 0.17 | -0.00 | 1.000 |
| Technique Information | 0.17 | -0.05 – 0.39 | 1.54 | 0.124 | 0.14 | -0.08 – 0.35 | 1.25 | 0.212 | -0.11 | -0.28 – 0.07 | -1.19 | 0.232 |
| Art Experience | 0.69 | 0.59 – 0.79 | 13.15 | **<0.001** | 0.61 | 0.51 – 0.71 | 12.15 | **<0.001** | 0.38 | 0.26 – 0.51 | 6.07 | **<0.001** |
| Age | 0.03 | -0.06 – 0.12 | 0.64 | 0.525 | 0.01 | -0.07 – 0.10 | 0.31 | 0.757 | 0.00 | -0.11 – 0.11 | 0.05 | 0.957 |
| Education | -0.03 | -0.12 – 0.06 | -0.60 | 0.550 | 0.02 | -0.07 – 0.11 | 0.40 | 0.691 | -0.05 | -0.16 – 0.06 | -0.85 | 0.393 |
| Openness to Experience | 0.13 | 0.02 – 0.23 | 2.42 | **0.016** | 0.13 | 0.03 – 0.23 | 2.61 | **0.009** | 0.08 | -0.04 – 0.21 | 1.34 | 0.182 |
| **Random Effects** | | | | | | | | | | | | |
| σ^2^ | 0.66 | | | | 0.63 | | | | 0.57 | | | |
| τ_00_ | 0.38 _sid_ | | | | 0.35 _sid_ | | | | 0.58 _sid_ | | | |
|  | 0.02 _itemno_ | | | | 0.02 _itemno_ | | | | 0.01 _itemno_ | | | |
| ICC | 0.38 | | | | 0.37 | | | | 0.51 | | | |
| N | 196 _sid_ | | | | 196 _sid_ | | | | 196 _sid_ | | | |
|  | 16 _itemno_ | | | | 16 _itemno_ | | | | 16 _itemno_ | | | |
| Observations | 3136 | | | | 3136 | | | | 3136 | | | |
| Marginal R^2^ / Conditional R^2^ | 0.289 / 0.559 | | | | 0.255 / 0.532 | | | | 0.101 / 0.561 | | | |

Table S8. Beta values, confidence intervals, p-values for the *art_experience* model.

|  | **liking** | | | | **beauty** | | | | **complexity** | | | |
| --- | --- | --- | --- | --- | --- | --- | --- | --- | --- | --- | --- | --- |
| *Predictors* | *Estimates* | *CI* | *Statistic* | *p* | *Estimates* | *CI* | *Statistic* | *p* | *Estimates* | *CI* | *Statistic* | *p* |
| Intercept | 3.36 | 3.18 – 3.53 | 37.11 | **<0.001** | 3.49 | 3.31 – 3.66 | 39.48 | **<0.001** | 3.66 | 3.50 – 3.83 | 43.55 | **<0.001** |
| Content Information | 0.20 | -0.02 – 0.41 | 1.78 | 0.075 | 0.08 | -0.14 – 0.29 | 0.70 | 0.485 | -0.19 | -0.37 – -0.02 | -2.17 | **0.030** |
| Artist Information | 0.37 | 0.15 – 0.59 | 3.36 | **0.001** | 0.35 | 0.13 – 0.56 | 3.19 | **0.001** | 0.00 | -0.17 – 0.18 | 0.05 | 0.958 |
| Technique Information | 0.18 | -0.03 – 0.40 | 1.66 | 0.098 | 0.15 | -0.06 – 0.37 | 1.43 | 0.153 | -0.11 | -0.28 – 0.06 | -1.25 | 0.211 |
| Art Experience | 1.65 | 1.42 – 1.89 | 13.67 | **<0.001** | 1.49 | 1.26 – 1.72 | 12.80 | **<0.001** | 0.65 | 0.38 – 0.93 | 4.66 | **<0.001** |
| Age | 0.03 | -0.06 – 0.13 | 0.70 | 0.486 | 0.02 | -0.07 – 0.11 | 0.38 | 0.704 | 0.01 | -0.11 – 0.12 | 0.13 | 0.900 |
| Education | -0.01 | -0.10 – 0.09 | -0.12 | 0.906 | 0.04 | -0.05 – 0.13 | 0.84 | 0.403 | -0.03 | -0.15 – 0.08 | -0.56 | 0.578 |
| Openness to Experience | 0.15 | 0.04 – 0.25 | 2.68 | **0.007** | 0.15 | 0.04 – 0.25 | 2.76 | **0.006** | 0.08 | -0.05 – 0.21 | 1.21 | 0.227 |
| Content Information x Art Experience | -0.28 | -0.44 – -0.12 | -3.43 | **0.001** | -0.15 | -0.30 – 0.01 | -1.84 | 0.065 | 0.17 | 0.02 – 0.32 | 2.24 | **0.025** |
| Artist Information x Art Experience | -0.54 | -0.70 – -0.38 | -6.58 | **<0.001** | -0.58 | -0.74 – -0.43 | -7.35 | **<0.001** | -0.10 | -0.25 – 0.05 | -1.35 | 0.177 |
| Technique Information x Art Experience | -0.29 | -0.45 – -0.13 | -3.49 | **<0.001** | -0.43 | -0.58 – -0.27 | -5.37 | **<0.001** | 0.11 | -0.04 – 0.26 | 1.44 | 0.151 |
| **Random Effects** | | | | | | | | | | | | |
| σ^2^ | 0.65 | | | | 0.61 | | | | 0.57 | | | |
| τ_00_ | 0.41 _sid_ | | | | 0.37 _sid_ | | | | 0.61 _sid_ | | | |
|  | 0.02 _itemno_ | | | | 0.02 _itemno_ | | | | 0.01 _itemno_ | | | |
| ICC | 0.39 | | | | 0.39 | | | | 0.52 | | | |
| N | 196 _sid_ | | | | 196 _sid_ | | | | 196 _sid_ | | | |
|  | 16 _itemno_ | | | | 16 _itemno_ | | | | 16 _itemno_ | | | |
| Observations | 3136 | | | | 3136 | | | | 3136 | | | |
| Marginal R^2^ / Conditional R^2^ | 0.282 / 0.565 | | | | 0.248 / 0.543 | | | | 0.084 / 0.563 | | | |

Table S9. Beta values, confidence intervals, p-values for the *content* model.

|  | **liking** | | | | **beauty** | | | | **complexity** | | | |
| --- | --- | --- | --- | --- | --- | --- | --- | --- | --- | --- | --- | --- |
| *Predictors* | *Estimates* | *CI* | *Statistic* | *p* | *Estimates* | *CI* | *Statistic* | *p* | *Estimates* | *CI* | *Statistic* | *p* |
| Intercept | 3.62 | 3.52 – 3.72 | 71.22 | **<0.001** | 3.63 | 3.53 – 3.73 | 70.19 | **<0.001** | 3.51 | 3.39 – 3.63 | 57.41 | **<0.001** |
| Content Type | -0.06 | -0.17 – 0.05 | -1.02 | 0.306 | 0.07 | -0.04 – 0.18 | 1.30 | 0.193 | -0.12 | -0.23 – -0.01 | -2.13 | **0.034** |
| Art Experience | 0.64 | 0.53 – 0.75 | 11.19 | **<0.001** | 0.62 | 0.51 – 0.74 | 10.71 | **<0.001** | 0.44 | 0.30 – 0.57 | 6.35 | **<0.001** |
| Age | 0.04 | -0.06 – 0.15 | 0.87 | 0.383 | -0.01 | -0.11 – 0.10 | -0.10 | 0.919 | -0.01 | -0.13 – 0.11 | -0.17 | 0.862 |
| Education | -0.03 | -0.14 – 0.07 | -0.66 | 0.510 | 0.00 | -0.10 – 0.10 | 0.04 | 0.969 | -0.07 | -0.19 – 0.06 | -1.06 | 0.290 |
| Openness to Experience | 0.02 | -0.09 – 0.14 | 0.43 | 0.668 | 0.02 | -0.09 – 0.14 | 0.40 | 0.692 | 0.04 | -0.09 – 0.18 | 0.60 | 0.547 |
| **Random Effects** | | | | | | | | | | | | |
| σ^2^ | 0.64 | | | | 0.59 | | | | 0.60 | | | |
| τ_00_ | 0.35 _sid_ | | | | 0.38 _sid_ | | | | 0.58 _sid_ | | | |
| ICC | 0.35 | | | | 0.39 | | | | 0.49 | | | |
| N | 196 _sid_ | | | | 196 _sid_ | | | | 196 _sid_ | | | |
| Observations | 784 | | | | 784 | | | | 784 | | | |
| Marginal R^2^ / Conditional R^2^ | 0.290 / 0.538 | | | | 0.283 / 0.563 | | | | 0.132 / 0.561 | | | |

Table S10. Beta values, confidence intervals, p-values for the *art_experience_content* model.

|  | **liking** | | | | **beauty** | | | | **complexity** | | | |
| --- | --- | --- | --- | --- | --- | --- | --- | --- | --- | --- | --- | --- |
| *Predictors* | *Estimates* | *CI* | *Statistic* | *p* | *Estimates* | *CI* | *Statistic* | *p* | *Estimates* | *CI* | *Statistic* | *p* |
| (Intercept) | 3.56 | 3.46 – 3.66 | 68.33 | **<0.001** | 3.57 | 3.46 – 3.67 | 67.95 | **<0.001** | 3.47 | 3.35 – 3.60 | 55.14 | **<0.001** |
| Age | 0.05 | -0.05 – 0.15 | 0.92 | 0.356 | -0.00 | -0.11 – 0.10 | -0.05 | 0.963 | -0.01 | -0.13 – 0.12 | -0.09 | 0.929 |
| Art Experience | 1.27 | 1.04 – 1.51 | 10.65 | **<0.001** | 1.26 | 1.02 – 1.49 | 10.42 | **<0.001** | 0.78 | 0.50 – 1.07 | 5.43 | **<0.001** |
| Type of Content | -0.06 | -0.17 – 0.05 | -1.02 | 0.306 | 0.07 | -0.04 – 0.17 | 1.21 | 0.228 | -0.13 | -0.24 – -0.02 | -2.36 | **0.019** |
| Type of Content x Art Experience | 0.01 | -0.22 – 0.23 | 0.05 | 0.960 | 0.11 | -0.11 – 0.33 | 1.00 | 0.317 | 0.28 | 0.06 – 0.50 | 2.53 | **0.012** |
| Education | -0.01 | -0.12 – 0.09 | -0.25 | 0.805 | 0.02 | -0.08 – 0.12 | 0.41 | 0.682 | -0.05 | -0.17 – 0.08 | -0.73 | 0.465 |
| Openness to Experience | 0.04 | -0.07 – 0.16 | 0.71 | 0.477 | 0.04 | -0.07 – 0.16 | 0.74 | 0.460 | 0.03 | -0.11 – 0.17 | 0.46 | 0.645 |
| **Random Effects** | | | | | | | | | | | | |
| σ^2^ | 0.64 | | | | 0.59 | | | | 0.59 | | | |
| τ_00_ | 0.36 _sid_ | | | | 0.39 _sid_ | | | | 0.62 _sid_ | | | |
| ICC | 0.36 | | | | 0.40 | | | | 0.51 | | | |
| N | 196 _sid_ | | | | 196 _sid_ | | | | 196 _sid_ | | | |
| Observations | 784 | | | | 784 | | | | 784 | | | |
| Marginal R^2^ / Conditional R^2^ | 0.276 / 0.538 | | | | 0.276 / 0.563 | | | | 0.109 / 0.565 | | | |

Table S11. Beta values, confidence intervals, p-values for the *openness_experience* model.

|  | **liking** | | | | **beauty** | | | | **complexity** | | | |
| --- | --- | --- | --- | --- | --- | --- | --- | --- | --- | --- | --- | --- |
| *Predictors* | *Estimates* | *CI* | *Statistic* | *p* | *Estimates* | *CI* | *Statistic* | *p* | *Estimates* | *CI* | *Statistic* | *p* |
| Intercept | 3.46 | 3.28 – 3.63 | 38.08 | **<0.001** | 3.59 | 3.41 – 3.76 | 40.90 | **<0.001** | 3.74 | 3.57 – 3.90 | 44.05 | **<0.001** |
| Content Information | 0.19 | -0.03 – 0.40 | 1.68 | 0.093 | 0.06 | -0.15 – 0.27 | 0.59 | 0.558 | -0.21 | -0.39 – -0.04 | -2.37 | **0.018** |
| Artist Information | 0.39 | 0.17 – 0.60 | 3.51 | **<0.001** | 0.37 | 0.16 – 0.58 | 3.41 | **0.001** | 0.00 | -0.17 – 0.18 | 0.00 | 0.997 |
| Technique Information | 0.18 | -0.04 – 0.40 | 1.63 | 0.104 | 0.16 | -0.05 – 0.37 | 1.45 | 0.147 | -0.12 | -0.30 – 0.05 | -1.40 | 0.162 |
| Openness to Experience | 0.21 | -0.04 – 0.45 | 1.65 | 0.098 | 0.24 | 0.01 – 0.47 | 2.01 | **0.045** | 0.32 | 0.04 – 0.60 | 2.24 | **0.025** |
| Age | 0.03 | -0.06 – 0.12 | 0.59 | 0.554 | 0.02 | -0.07 – 0.10 | 0.36 | 0.716 | 0.01 | -0.10 – 0.12 | 0.16 | 0.874 |
| Education | -0.03 | -0.12 – 0.07 | -0.56 | 0.577 | 0.02 | -0.07 – 0.11 | 0.41 | 0.684 | -0.05 | -0.16 – 0.06 | -0.84 | 0.399 |
| Art Experience | 0.71 | 0.60 – 0.81 | 12.88 | **<0.001** | 0.64 | 0.54 – 0.74 | 12.26 | **<0.001** | 0.40 | 0.28 – 0.53 | 6.14 | **<0.001** |
| Content Information x Openness to Experience | -0.00 | -0.17 – 0.16 | -0.01 | 0.990 | -0.08 | -0.24 – 0.08 | -0.95 | 0.342 | -0.20 | -0.35 – -0.05 | -2.55 | **0.011** |
| Artist Information x Openness to Experience | 0.28 | 0.11 – 0.45 | 3.30 | **0.001** | 0.37 | 0.21 – 0.53 | 4.50 | **<0.001** | -0.01 | -0.16 – 0.15 | -0.09 | 0.925 |
| Technique Information x Openness to Experience | 0.07 | -0.09 – 0.24 | 0.85 | 0.397 | 0.12 | -0.04 – 0.28 | 1.46 | 0.145 | -0.18 | -0.34 – -0.03 | -2.32 | **0.021** |
| **Random Effects** | | | | | | | | | | | | |
| σ^2^ | 0.66 | | | | 0.62 | | | | 0.57 | | | |
| τ_00_ | 0.38 _sid_ | | | | 0.34 _sid_ | | | | 0.57 _sid_ | | | |
|  | 0.02 _itemno_ | | | | 0.02 _itemno_ | | | | 0.01 _itemno_ | | | |
| ICC | 0.38 | | | | 0.37 | | | | 0.51 | | | |
| N | 198 _sid_ | | | | 198 _sid_ | | | | 198 _sid_ | | | |
|  | 16 _itemno_ | | | | 16 _itemno_ | | | | 16 _itemno_ | | | |
| Observations | 3168 | | | | 3168 | | | | 3168 | | | |
| Marginal R^2^ / Conditional R^2^ | 0.290 / 0.559 | | | | 0.263 / 0.534 | | | | 0.105 / 0.561 | | | |

Table S12. Beta values, confidence intervals, p-values for the *openness_experience_content* model.

|  | **liking** | | | | **beauty** | | | | **complexity** | | | |
| --- | --- | --- | --- | --- | --- | --- | --- | --- | --- | --- | --- | --- |
| *Predictors* | *Estimates* | *CI* | *Statistic* | *p* | *Estimates* | *CI* | *Statistic* | *p* | *Estimates* | *CI* | *Statistic* | *p* |
| Intercept | 3.64 | 3.53 – 3.74 | 68.90 | **<0.001** | 3.65 | 3.55 – 3.76 | 68.29 | **<0.001** | 3.53 | 3.41 – 3.66 | 55.60 | **<0.001** |
| Content Type | -0.04 | -0.16 – 0.08 | -0.69 | 0.492 | 0.06 | -0.05 – 0.18 | 1.15 | 0.251 | -0.14 | -0.26 – -0.03 | -2.52 | **0.012** |
| Openness to Experience | 0.17 | -0.07 – 0.41 | 1.38 | 0.169 | 0.19 | -0.06 – 0.43 | 1.49 | 0.136 | 0.19 | -0.10 – 0.48 | 1.30 | 0.195 |
| Age | 0.04 | -0.06 – 0.14 | 0.83 | 0.406 | -0.00 | -0.10 – 0.10 | -0.04 | 0.969 | -0.01 | -0.13 – 0.11 | -0.10 | 0.918 |
| Education | -0.04 | -0.14 – 0.06 | -0.73 | 0.465 | -0.00 | -0.10 – 0.10 | -0.06 | 0.952 | -0.07 | -0.19 – 0.05 | -1.09 | 0.277 |
| Art Experience | 0.67 | 0.55 – 0.79 | 11.25 | **<0.001** | 0.66 | 0.54 – 0.78 | 10.95 | **<0.001** | 0.47 | 0.33 – 0.61 | 6.53 | **<0.001** |
| Content Type x Openness to Experience | 0.09 | -0.14 – 0.33 | 0.80 | 0.422 | -0.01 | -0.23 – 0.22 | -0.06 | 0.955 | -0.10 | -0.32 – 0.13 | -0.84 | 0.402 |
| **Random Effects** | | | | | | | | | | | | |
| σ^2^ | 0.64 | | | | 0.59 | | | | 0.60 | | | |
| τ_00_ | 0.34 _sid_ | | | | 0.37 _sid_ | | | | 0.57 _sid_ | | | |
| ICC | 0.35 | | | | 0.38 | | | | 0.49 | | | |
| N | 198 _sid_ | | | | 198 _sid_ | | | | 198 _sid_ | | | |
| Observations | 792 | | | | 792 | | | | 792 | | | |
| Marginal R^2^ / Conditional R^2^ | 0.291 / 0.536 | | | | 0.288 / 0.562 | | | | 0.136 / 0.558 | | | |

Table S13. Beta values, confidence intervals, p-values for the *artwork_culture* model.

|  | **liking** | | | | **beauty** | | | | **complexity** | | | |
| --- | --- | --- | --- | --- | --- | --- | --- | --- | --- | --- | --- | --- |
| *Predictors* | *Estimates* | *CI* | *Statistic* | *p* | *Estimates* | *CI* | *Statistic* | *p* | *Estimates* | *CI* | *Statistic* | *p* |
| Intercept | 3.44 | 3.31 – 3.56 | 53.25 | **<0.001** | 3.56 | 3.44 – 3.68 | 59.71 | **<0.001** | 3.69 | 3.55 – 3.84 | 51.37 | **<0.001** |
| Content Information | 0.18 | 0.05 – 0.31 | 2.77 | **0.006** | 0.07 | -0.05 – 0.19 | 1.16 | 0.246 | -0.18 | -0.32 – -0.05 | -2.79 | **0.005** |
| Artist Information | 0.35 | 0.22 – 0.48 | 5.21 | **<0.001** | 0.32 | 0.20 – 0.44 | 5.37 | **<0.001** | -0.00 | -0.13 – 0.13 | -0.00 | 1.000 |
| Technique Information | 0.17 | 0.04 – 0.30 | 2.55 | **0.011** | 0.14 | 0.02 – 0.25 | 2.28 | **0.023** | -0.11 | -0.24 – 0.02 | -1.60 | 0.111 |
| Artwork Culture | 0.09 | -0.09 – 0.28 | 1.01 | 0.315 | -0.02 | -0.19 – 0.14 | -0.24 | 0.808 | -0.05 | -0.23 – 0.14 | -0.52 | 0.606 |
| Age | 0.03 | -0.06 – 0.12 | 0.64 | 0.524 | 0.01 | -0.07 – 0.10 | 0.31 | 0.757 | 0.00 | -0.11 – 0.11 | 0.05 | 0.957 |
| Education | -0.03 | -0.12 – 0.06 | -0.60 | 0.550 | 0.02 | -0.07 – 0.11 | 0.40 | 0.691 | -0.05 | -0.16 – 0.06 | -0.85 | 0.393 |
| Art Experience | 0.69 | 0.59 – 0.79 | 13.16 | **<0.001** | 0.61 | 0.51 – 0.71 | 12.16 | **<0.001** | 0.38 | 0.26 – 0.51 | 6.07 | **<0.001** |
| Openness to Experience | 0.13 | 0.02 – 0.23 | 2.42 | **0.016** | 0.13 | 0.03 – 0.23 | 2.61 | **0.009** | 0.08 | -0.04 – 0.21 | 1.34 | 0.182 |
| Content Information x Artwork Culture | -0.02 | -0.28 – 0.25 | -0.12 | 0.908 | -0.01 | -0.24 – 0.23 | -0.04 | 0.966 | 0.14 | -0.13 – 0.40 | 1.02 | 0.308 |
| Artist Information x Artwork Culture | -0.20 | -0.46 – 0.06 | -1.48 | 0.139 | -0.10 | -0.33 – 0.14 | -0.82 | 0.414 | 0.21 | -0.05 – 0.47 | 1.58 | 0.115 |
| Technique Information x Artwork Culture | 0.37 | 0.11 – 0.63 | 2.79 | **0.005** | 0.51 | 0.28 – 0.74 | 4.30 | **<0.001** | 0.31 | 0.05 – 0.57 | 2.36 | **0.018** |
| **Random Effects** | | | | | | | | | | | | |
| σ^2^ | 0.66 | | | | 0.63 | | | | 0.57 | | | |
| τ_00_ | 0.38 _sid_ | | | | 0.35 _sid_ | | | | 0.58 _sid_ | | | |
|  | 0.01 _itemno_ | | | | 0.00 _itemno_ | | | | 0.01 _itemno_ | | | |
| ICC | 0.37 | | | | 0.36 | | | | 0.51 | | | |
| N | 196 _sid_ | | | | 196 _sid_ | | | | 196 _sid_ | | | |
|  | 16 _itemno_ | | | | 16 _itemno_ | | | | 16 _itemno_ | | | |
| Observations | 3136 | | | | 3136 | | | | 3136 | | | |
| Marginal R^2^ / Conditional R^2^ | 0.299 / 0.559 | | | | 0.267 / 0.532 | | | | 0.106 / 0.561 | | | |

Table S14. Beta values, confidence intervals, p-values for the *motion* model.

|  | **liking** | | | | **beauty** | | | | **complexity** | | | |
| --- | --- | --- | --- | --- | --- | --- | --- | --- | --- | --- | --- | --- |
| *Predictors* | *Estimates* | *CI* | *Statistic* | *p* | *Estimates* | *CI* | *Statistic* | *p* | *Estimates* | *CI* | *Statistic* | *p* |
| Intercept | 3.44 | 3.28 – 3.59 | 43.98 | **<0.001** | 3.56 | 3.40 – 3.72 | 42.39 | **<0.001** | 3.69 | 3.55 – 3.84 | 51.51 | **<0.001** |
| Content Information | 0.17 | -0.03 – 0.36 | 1.70 | 0.089 | 0.08 | -0.14 – 0.29 | 0.69 | 0.490 | -0.24 | -0.38 – -0.10 | -3.42 | **0.001** |
| Artist Information | 0.35 | 0.17 – 0.52 | 3.80 | **<0.001** | 0.32 | 0.12 – 0.52 | 3.11 | **0.002** | -0.00 | -0.13 – 0.13 | -0.00 | 1.000 |
| Technique Information | 0.22 | 0.03 – 0.42 | 2.26 | **0.024** | 0.18 | -0.04 – 0.40 | 1.64 | 0.100 | -0.04 | -0.18 – 0.10 | -0.58 | 0.563 |
| Motion | -0.07 | -0.33 – 0.18 | -0.57 | 0.566 | -0.02 | -0.30 – 0.27 | -0.11 | 0.916 | 0.12 | -0.06 – 0.31 | 1.34 | 0.180 |
| Age | 0.03 | -0.06 – 0.12 | 0.64 | 0.525 | 0.01 | -0.07 – 0.10 | 0.31 | 0.757 | 0.00 | -0.11 – 0.11 | 0.05 | 0.957 |
| Education | -0.03 | -0.12 – 0.06 | -0.60 | 0.550 | 0.02 | -0.07 – 0.11 | 0.40 | 0.691 | -0.05 | -0.16 – 0.06 | -0.85 | 0.393 |
| Art Experience | 0.69 | 0.59 – 0.79 | 13.16 | **<0.001** | 0.61 | 0.51 – 0.71 | 12.15 | **<0.001** | 0.38 | 0.26 – 0.51 | 6.07 | **<0.001** |
| Openness to Experience | 0.13 | 0.02 – 0.23 | 2.42 | **0.016** | 0.13 | 0.03 – 0.23 | 2.61 | **0.009** | 0.08 | -0.04 – 0.21 | 1.34 | 0.182 |
| Content Information x Motion | 0.14 | -0.25 – 0.53 | 0.71 | 0.476 | -0.02 | -0.45 – 0.42 | -0.07 | 0.945 | 0.11 | -0.17 – 0.39 | 0.77 | 0.441 |
| Artist Information x Motion | 0.35 | -0.00 – 0.71 | 1.95 | 0.052 | 0.12 | -0.28 – 0.52 | 0.57 | 0.567 | -0.15 | -0.41 – 0.11 | -1.16 | 0.246 |
| Technique Information x Motion | 0.28 | -0.10 – 0.67 | 1.45 | 0.148 | 0.20 | -0.23 – 0.64 | 0.91 | 0.361 | 0.13 | -0.15 – 0.41 | 0.94 | 0.349 |
| **Random Effects** | | | | | | | | | | | | |
| σ^2^ | 0.66 | | | | 0.63 | | | | 0.57 | | | |
| τ_00_ | 0.38 _sid_ | | | | 0.35 _sid_ | | | | 0.58 _sid_ | | | |
|  | 0.01 _itemno_ | | | | 0.02 _itemno_ | | | | 0.01 _itemno_ | | | |
| ICC | 0.37 | | | | 0.37 | | | | 0.51 | | | |
| N | 196 _sid_ | | | | 196 _sid_ | | | | 196 _sid_ | | | |
|  | 16 _itemno_ | | | | 16 _itemno_ | | | | 16 _itemno_ | | | |
| Observations | 3136 | | | | 3136 | | | | 3136 | | | |
| Marginal R^2^ / Conditional R^2^ | 0.294 / 0.559 | | | | 0.257 / 0.532 | | | | 0.106 / 0.561 | | | |

Table S15. Beta values, confidence intervals, p-values for the *info_type* model for aesthetic impacts.

|  | **angry** | | | | **calm** | | | | **compassionate** | | | | **challenging** | | | | **edified** | | | |
| --- | --- | --- | --- | --- | --- | --- | --- | --- | --- | --- | --- | --- | --- | --- | --- | --- | --- | --- | --- | --- |
| *Predictors* | *Estimates* | *CI* | *Statistic* | *p* | *Estimates* | *CI* | *Statistic* | *p* | *Estimates* | *CI* | *Statistic* | *p* | *Estimates* | *CI* | *Statistic* | *p* | *Estimates* | *CI* | *Statistic* | *p* |
| Intercept | 2.02 | 1.88 – 2.16 | 27.37 | **<0.001** | 3.06 | 2.88 – 3.24 | 33.78 | **<0.001** | 3.16 | 3.00 – 3.33 | 36.95 | **<0.001** | 3.04 | 2.90 – 3.18 | 42.13 | **<0.001** | 2.74 | 2.59 – 2.89 | 36.71 | **<0.001** |
| Content Information | -0.18 | -0.30 – -0.07 | -3.17 | **0.002** | 0.33 | 0.12 – 0.54 | 3.03 | **0.002** | -0.04 | -0.22 – 0.14 | -0.40 | 0.690 | -0.13 | -0.26 – -0.01 | -2.08 | **0.038** | -0.00 | -0.11 – 0.11 | -0.08 | 0.938 |
| Artist Information | -0.25 | -0.36 – -0.13 | -4.24 | **<0.001** | 0.53 | 0.32 – 0.75 | 4.90 | **<0.001** | 0.10 | -0.08 – 0.28 | 1.07 | 0.285 | -0.16 | -0.29 – -0.04 | -2.58 | **0.010** | 0.07 | -0.04 – 0.18 | 1.21 | 0.228 |
| Technique Information | -0.12 | -0.24 – -0.01 | -2.10 | **0.036** | 0.28 | 0.07 – 0.50 | 2.61 | **0.009** | 0.05 | -0.13 – 0.23 | 0.56 | 0.579 | -0.12 | -0.25 – 0.00 | -1.96 | 0.050 | 0.05 | -0.06 – 0.16 | 0.87 | 0.387 |
| Art Experience | 0.50 | 0.37 – 0.64 | 7.17 | **<0.001** | 0.61 | 0.50 – 0.72 | 10.89 | **<0.001** | 0.64 | 0.51 – 0.76 | 9.77 | **<0.001** | 0.84 | 0.71 – 0.96 | 12.79 | **<0.001** | 0.65 | 0.51 – 0.80 | 8.95 | **<0.001** |
| Age | 0.04 | -0.09 – 0.16 | 0.56 | 0.576 | -0.02 | -0.12 – 0.08 | -0.43 | 0.666 | 0.10 | -0.02 – 0.21 | 1.65 | 0.099 | 0.09 | -0.03 – 0.20 | 1.46 | 0.146 | 0.15 | 0.02 – 0.27 | 2.23 | **0.026** |
| Education | -0.13 | -0.25 – -0.01 | -2.06 | **0.039** | -0.03 | -0.13 – 0.07 | -0.53 | 0.596 | -0.04 | -0.16 – 0.07 | -0.73 | 0.465 | -0.05 | -0.16 – 0.07 | -0.78 | 0.438 | -0.05 | -0.18 – 0.08 | -0.77 | 0.439 |
| Openness to Experience | -0.28 | -0.42 – -0.14 | -3.99 | **<0.001** | 0.06 | -0.05 – 0.17 | 1.12 | 0.262 | 0.08 | -0.05 – 0.21 | 1.20 | 0.229 | -0.11 | -0.23 – 0.02 | -1.62 | 0.105 | -0.07 | -0.21 – 0.07 | -0.99 | 0.323 |
| **Random Effects** | | | | | | | | | | | | | | | | | | | | |
| σ^2^ | 0.44 | | | | 0.72 | | | | 0.70 | | | | 0.58 | | | | 0.57 | | | |
| τ_00_ | 0.73 _sid_ | | | | 0.44 _sid_ | | | | 0.61 _sid_ | | | | 0.62 _sid_ | | | | 0.79 _sid_ | | | |
|  | 0.00 _itemno_ | | | | 0.02 _itemno_ | | | | 0.01 _itemno_ | | | | 0.01 _itemno_ | | | | 0.00 _itemno_ | | | |
| ICC | 0.62 | | | | 0.39 | | | | 0.47 | | | | 0.52 | | | | 0.58 | | | |
| N | 196 _sid_ | | | | 196 _sid_ | | | | 196 _sid_ | | | | 196 _sid_ | | | | 196 _sid_ | | | |
|  | 16 _itemno_ | | | | 16 _itemno_ | | | | 16 _itemno_ | | | | 16 _itemno_ | | | | 16 _itemno_ | | | |
| Observations | 3136 | | | | 3136 | | | | 3135 | | | | 3136 | | | | 3135 | | | |
| Marginal R^2^ / Conditional R^2^ | 0.287 / 0.732 | | | | 0.243 / 0.541 | | | | 0.225 / 0.590 | | | | 0.401 / 0.713 | | | | 0.271 / 0.695 | | | |

|  | **enraptured** | | | | **enlightened** | | | | **interested** | | | | **inspired** | | | | **pleasure** | | | | **upset** | | | |
| --- | --- | --- | --- | --- | --- | --- | --- | --- | --- | --- | --- | --- | --- | --- | --- | --- | --- | --- | --- | --- | --- | --- | --- | --- |
| *Predictors* | *Estimates* | *CI* | *Statistic* | *p* | *Estimates* | *CI* | *Statistic* | *p* | *Estimates* | *CI* | *Statistic* | *p* | *Estimates* | *CI* | *Statistic* | *p* | *Estimates* | *CI* | *Statistic* | *p* | *Estimates* | *CI* | *Statistic* | *p* |
| Intercept | 2.87 | 2.70 – 3.04 | 33.95 | **<0.001** | 2.93 | 2.78 – 3.08 | 37.68 | **<0.001** | 3.44 | 3.27 – 3.60 | 40.56 | **<0.001** | 3.09 | 2.93 – 3.25 | 37.84 | **<0.001** | 2.97 | 2.80 – 3.15 | 33.42 | **<0.001** | 2.20 | 2.03 – 2.38 | 24.48 | **<0.001** |
| Content Information | -0.05 | -0.22 – 0.12 | -0.57 | 0.566 | 0.16 | 0.01 – 0.31 | 2.11 | **0.035** | 0.14 | -0.06 – 0.34 | 1.38 | 0.167 | 0.17 | -0.01 – 0.35 | 1.85 | 0.064 | 0.31 | 0.11 – 0.52 | 2.97 | **0.003** | -0.28 | -0.47 – -0.10 | -3.04 | **0.002** |
| Artist Information | 0.13 | -0.04 – 0.30 | 1.53 | 0.126 | 0.22 | 0.07 – 0.36 | 2.88 | **0.004** | 0.22 | 0.03 – 0.42 | 2.22 | **0.026** | 0.32 | 0.14 – 0.50 | 3.55 | **<0.001** | 0.50 | 0.30 – 0.71 | 4.80 | **<0.001** | -0.37 | -0.55 – -0.19 | -3.94 | **<0.001** |
| Technique Information | 0.06 | -0.11 – 0.23 | 0.66 | 0.508 | 0.15 | 0.00 – 0.30 | 2.01 | **0.045** | 0.12 | -0.07 – 0.32 | 1.23 | 0.218 | 0.19 | 0.02 – 0.37 | 2.13 | **0.033** | 0.28 | 0.07 – 0.48 | 2.63 | **0.008** | -0.26 | -0.44 – -0.07 | -2.73 | **0.006** |
| Art Experience | 0.64 | 0.51 – 0.77 | 9.46 | **<0.001** | 0.70 | 0.58 – 0.83 | 10.67 | **<0.001** | 0.56 | 0.46 – 0.67 | 10.32 | **<0.001** | 0.84 | 0.72 – 0.95 | 14.18 | **<0.001** | 0.83 | 0.72 – 0.94 | 14.46 | **<0.001** | 0.51 | 0.37 – 0.65 | 7.19 | **<0.001** |
| Age | 0.11 | -0.01 – 0.22 | 1.74 | 0.082 | 0.09 | -0.02 – 0.21 | 1.56 | 0.118 | 0.08 | -0.02 – 0.18 | 1.63 | 0.103 | -0.02 | -0.13 – 0.08 | -0.45 | 0.651 | 0.03 | -0.07 – 0.13 | 0.54 | 0.589 | 0.04 | -0.09 – 0.16 | 0.58 | 0.565 |
| Education | -0.06 | -0.18 – 0.06 | -0.92 | 0.356 | -0.05 | -0.16 – 0.07 | -0.81 | 0.416 | 0.01 | -0.09 – 0.10 | 0.15 | 0.880 | -0.02 | -0.13 – 0.08 | -0.44 | 0.662 | -0.04 | -0.14 – 0.06 | -0.78 | 0.434 | -0.12 | -0.25 – 0.00 | -1.94 | 0.052 |
| Openness to Experience | -0.08 | -0.21 – 0.06 | -1.14 | 0.256 | 0.09 | -0.04 – 0.22 | 1.37 | 0.172 | 0.14 | 0.03 – 0.24 | 2.52 | **0.012** | 0.09 | -0.03 – 0.20 | 1.48 | 0.138 | 0.08 | -0.03 – 0.20 | 1.44 | 0.149 | -0.28 | -0.42 – -0.14 | -3.97 | **<0.001** |
| **Random Effects** | | | | | | | | | | | | | | | | | | | | | | | | |
| σ^2^ | 0.68 | | | | 0.63 | | | | 0.69 | | | | 0.67 | | | | 0.68 | | | | 0.54 | | | |
| τ_00_ | 0.66 _sid_ | | | | 0.63 _sid_ | | | | 0.42 _sid_ | | | | 0.49 _sid_ | | | | 0.47 _sid_ | | | | 0.73 _sid_ | | | |
|  | 0.01 _itemno_ | | | | 0.01 _itemno_ | | | | 0.02 _itemno_ | | | | 0.01 _itemno_ | | | | 0.02 _itemno_ | | | | 0.01 _itemno_ | | | |
| ICC | 0.50 | | | | 0.50 | | | | 0.39 | | | | 0.43 | | | | 0.42 | | | | 0.58 | | | |
| N | 196 _sid_ | | | | 196 _sid_ | | | | 196 _sid_ | | | | 196 _sid_ | | | | 196 _sid_ | | | | 196 _sid_ | | | |
|  | 16 _itemno_ | | | | 16 _itemno_ | | | | 16 _itemno_ | | | | 16 _itemno_ | | | | 16 _itemno_ | | | | 16 _itemno_ | | | |
| Observations | 3136 | | | | 3136 | | | | 3136 | | | | 3136 | | | | 3136 | | | | 3136 | | | |
| Marginal R^2^ / Conditional R^2^ | 0.262 / 0.629 | | | | 0.269 / 0.636 | | | | 0.206 / 0.515 | | | | 0.357 / 0.634 | | | | 0.365 / 0.630 | | | | 0.275 / 0.694 | | | |

Table S16. Beta values, confidence intervals, p-values for the *art_experience* model for aesthetic impacts.

|  | **angry** | | | | **calm** | | | | **compassionate** | | | | **challenging** | | | | **edified** | | | |
| --- | --- | --- | --- | --- | --- | --- | --- | --- | --- | --- | --- | --- | --- | --- | --- | --- | --- | --- | --- | --- |
| *Predictors* | *Estimates* | *CI* | *Statistic* | *p* | *Estimates* | *CI* | *Statistic* | *p* | *Estimates* | *CI* | *Statistic* | *p* | *Estimates* | *CI* | *Statistic* | *p* | *Estimates* | *CI* | *Statistic* | *p* |
| Intercept | 1.98 | 1.83 – 2.12 | 26.55 | **<0.001** | 2.98 | 2.80 – 3.16 | 32.33 | **<0.001** | 3.10 | 2.93 – 3.27 | 36.06 | **<0.001** | 2.97 | 2.83 – 3.11 | 41.51 | **<0.001** | 2.67 | 2.53 – 2.82 | 35.49 | **<0.001** |
| Content Information | -0.19 | -0.30 – -0.07 | -3.24 | **0.001** | 0.35 | 0.14 – 0.57 | 3.23 | **0.001** | -0.04 | -0.22 – 0.14 | -0.44 | 0.659 | -0.15 | -0.27 – -0.02 | -2.34 | **0.019** | -0.00 | -0.11 – 0.11 | -0.07 | 0.940 |
| Artist Information | -0.25 | -0.37 – -0.14 | -4.34 | **<0.001** | 0.57 | 0.35 – 0.78 | 5.21 | **<0.001** | 0.10 | -0.08 – 0.28 | 1.08 | 0.281 | -0.18 | -0.31 – -0.06 | -2.87 | **0.004** | 0.07 | -0.04 – 0.18 | 1.32 | 0.188 |
| Technique Information | -0.13 | -0.25 – -0.02 | -2.27 | **0.023** | 0.31 | 0.09 – 0.52 | 2.82 | **0.005** | 0.05 | -0.13 – 0.23 | 0.59 | 0.557 | -0.14 | -0.26 – -0.01 | -2.14 | **0.032** | 0.06 | -0.05 – 0.17 | 1.03 | 0.301 |
| Art Experience | 0.90 | 0.60 – 1.19 | 5.95 | **<0.001** | 1.57 | 1.32 – 1.83 | 11.96 | **<0.001** | 1.29 | 1.01 – 1.57 | 9.02 | **<0.001** | 1.49 | 1.22 – 1.77 | 10.74 | **<0.001** | 1.41 | 1.10 – 1.72 | 8.96 | **<0.001** |
| Age | 0.04 | -0.09 – 0.16 | 0.60 | 0.552 | -0.02 | -0.12 – 0.09 | -0.30 | 0.763 | 0.10 | -0.02 – 0.21 | 1.69 | 0.092 | 0.09 | -0.03 – 0.20 | 1.51 | 0.130 | 0.15 | 0.02 – 0.28 | 2.26 | **0.024** |
| Education | -0.11 | -0.24 – 0.01 | -1.81 | 0.071 | -0.00 | -0.11 – 0.10 | -0.04 | 0.968 | -0.02 | -0.14 – 0.09 | -0.40 | 0.686 | -0.02 | -0.14 – 0.09 | -0.41 | 0.680 | -0.03 | -0.16 – 0.10 | -0.47 | 0.640 |
| Openness to Experience | -0.26 | -0.40 – -0.12 | -3.67 | **<0.001** | 0.06 | -0.06 – 0.18 | 1.03 | 0.305 | 0.10 | -0.03 – 0.23 | 1.55 | 0.121 | -0.06 | -0.19 – 0.07 | -0.92 | 0.357 | -0.05 | -0.19 – 0.10 | -0.64 | 0.520 |
| Content Information x Art Experience | 0.10 | -0.03 – 0.23 | 1.46 | 0.144 | -0.48 | -0.65 – -0.31 | -5.68 | **<0.001** | 0.09 | -0.08 – 0.25 | 1.03 | 0.304 | 0.37 | 0.22 – 0.52 | 4.82 | **<0.001** | -0.00 | -0.15 – 0.15 | -0.04 | 0.971 |
| Artist Information x Art Experience | 0.13 | 0.00 – 0.27 | 1.98 | **0.048** | -0.74 | -0.91 – -0.57 | -8.74 | **<0.001** | -0.02 | -0.19 – 0.15 | -0.24 | 0.807 | 0.41 | 0.26 – 0.56 | 5.33 | **<0.001** | -0.14 | -0.29 – 0.01 | -1.82 | 0.070 |
| Technique Information x Art Experience | 0.22 | 0.09 – 0.36 | 3.31 | **0.001** | -0.51 | -0.68 – -0.35 | -6.06 | **<0.001** | -0.07 | -0.23 – 0.10 | -0.78 | 0.437 | 0.26 | 0.11 – 0.41 | 3.39 | **0.001** | -0.21 | -0.36 – -0.06 | -2.75 | **0.006** |
| **Random Effects** | | | | | | | | | | | | | | | | | | | | |
| σ^2^ | 0.44 | | | | 0.70 | | | | 0.70 | | | | 0.57 | | | | 0.57 | | | |
| τ_00_ | 0.74 _sid_ | | | | 0.50 _sid_ | | | | 0.61 _sid_ | | | | 0.60 _sid_ | | | | 0.79 _sid_ | | | |
|  | 0.00 _itemno_ | | | | 0.02 _itemno_ | | | | 0.01 _itemno_ | | | | 0.01 _itemno_ | | | | 0.00 _itemno_ | | | |
| ICC | 0.63 | | | | 0.43 | | | | 0.47 | | | | 0.51 | | | | 0.58 | | | |
| N | 196 _sid_ | | | | 196 _sid_ | | | | 196 _sid_ | | | | 196 _sid_ | | | | 196 _sid_ | | | |
|  | 16 _itemno_ | | | | 16 _itemno_ | | | | 16 _itemno_ | | | | 16 _itemno_ | | | | 16 _itemno_ | | | |
| Observations | 3136 | | | | 3136 | | | | 3135 | | | | 3136 | | | | 3135 | | | |
| Marginal R^2^ / Conditional R^2^ | 0.283 / 0.733 | | | | 0.222 / 0.553 | | | | 0.223 / 0.590 | | | | 0.417 / 0.716 | | | | 0.268 / 0.696 | | | |

|  | **enraptured** | | | | **enlightened** | | | | **interested** | | | | **inspired** | | | | **pleasure** | | | | **upset** | | | |
| --- | --- | --- | --- | --- | --- | --- | --- | --- | --- | --- | --- | --- | --- | --- | --- | --- | --- | --- | --- | --- | --- | --- | --- | --- |
| *Predictors* | *Estimates* | *CI* | *Statistic* | *p* | *Estimates* | *CI* | *Statistic* | *p* | *Estimates* | *CI* | *Statistic* | *p* | *Estimates* | *CI* | *Statistic* | *p* | *Estimates* | *CI* | *Statistic* | *p* | *Estimates* | *CI* | *Statistic* | *p* |
| Intercept | 2.80 | 2.64 – 2.97 | 32.56 | **<0.001** | 2.85 | 2.70 – 3.01 | 35.91 | **<0.001** | 3.38 | 3.21 – 3.54 | 39.49 | **<0.001** | 2.99 | 2.83 – 3.16 | 35.67 | **<0.001** | 2.88 | 2.70 – 3.06 | 31.47 | **<0.001** | 2.16 | 1.98 – 2.34 | 23.91 | **<0.001** |
| Content Information | -0.05 | -0.22 – 0.12 | -0.55 | 0.585 | 0.16 | 0.02 – 0.31 | 2.19 | **0.029** | 0.14 | -0.05 – 0.34 | 1.44 | 0.151 | 0.18 | 0.00 – 0.36 | 2.01 | **0.044** | 0.33 | 0.12 – 0.54 | 3.13 | **0.002** | -0.29 | -0.48 – -0.11 | -3.13 | **0.002** |
| Artist Information | 0.14 | -0.03 – 0.31 | 1.64 | 0.101 | 0.22 | 0.08 – 0.37 | 2.99 | **0.003** | 0.23 | 0.04 – 0.43 | 2.31 | **0.021** | 0.34 | 0.17 – 0.52 | 3.78 | **<0.001** | 0.54 | 0.33 – 0.74 | 5.10 | **<0.001** | -0.38 | -0.56 – -0.19 | -4.04 | **<0.001** |
| Technique Information | 0.07 | -0.10 – 0.24 | 0.80 | 0.426 | 0.17 | 0.02 – 0.31 | 2.21 | **0.027** | 0.13 | -0.07 – 0.33 | 1.28 | 0.200 | 0.22 | 0.04 – 0.40 | 2.38 | **0.017** | 0.30 | 0.09 – 0.50 | 2.81 | **0.005** | -0.27 | -0.45 – -0.08 | -2.83 | **0.005** |
| Art Experience | 1.35 | 1.05 – 1.65 | 8.90 | **<0.001** | 1.52 | 1.23 – 1.81 | 10.25 | **<0.001** | 1.22 | 0.97 – 1.47 | 9.74 | **<0.001** | 1.92 | 1.66 – 2.19 | 14.01 | **<0.001** | 1.95 | 1.69 – 2.22 | 14.35 | **<0.001** | 0.88 | 0.58 – 1.17 | 5.79 | **<0.001** |
| Age | 0.11 | -0.01 – 0.23 | 1.77 | 0.076 | 0.10 | -0.02 – 0.22 | 1.60 | 0.110 | 0.08 | -0.01 – 0.18 | 1.67 | 0.095 | -0.02 | -0.13 – 0.09 | -0.31 | 0.754 | 0.03 | -0.07 – 0.14 | 0.63 | 0.529 | 0.04 | -0.09 – 0.16 | 0.60 | 0.546 |
| Education | -0.03 | -0.15 – 0.09 | -0.51 | 0.607 | -0.02 | -0.14 – 0.10 | -0.37 | 0.712 | 0.03 | -0.07 – 0.12 | 0.53 | 0.594 | 0.01 | -0.10 – 0.12 | 0.14 | 0.891 | -0.01 | -0.12 – 0.10 | -0.16 | 0.876 | -0.11 | -0.23 – 0.02 | -1.71 | 0.087 |
| Openness to Experience | -0.07 | -0.21 – 0.07 | -1.00 | 0.315 | 0.10 | -0.04 – 0.24 | 1.43 | 0.153 | 0.15 | 0.04 – 0.26 | 2.66 | **0.008** | 0.10 | -0.03 – 0.22 | 1.52 | 0.128 | 0.09 | -0.04 – 0.21 | 1.40 | 0.163 | -0.26 | -0.40 – -0.12 | -3.60 | **<0.001** |
| Content Information x Art Experience | -0.05 | -0.21 – 0.11 | -0.60 | 0.550 | -0.13 | -0.28 – 0.03 | -1.58 | 0.115 | -0.12 | -0.29 – 0.04 | -1.44 | 0.149 | -0.33 | -0.49 – -0.17 | -4.01 | **<0.001** | -0.37 | -0.54 – -0.21 | -4.54 | **<0.001** | 0.19 | 0.05 – 0.34 | 2.60 | **0.009** |
| Artist Information x Art Experience | -0.21 | -0.37 – -0.05 | -2.51 | **0.012** | -0.19 | -0.34 – -0.03 | -2.32 | **0.021** | -0.20 | -0.37 – -0.04 | -2.43 | **0.015** | -0.46 | -0.63 – -0.30 | -5.64 | **<0.001** | -0.73 | -0.89 – -0.57 | -8.83 | **<0.001** | 0.21 | 0.07 – 0.36 | 2.88 | **0.004** |
| Technique Information x Art Experience | -0.26 | -0.42 – -0.09 | -3.05 | **0.002** | -0.34 | -0.49 – -0.18 | -4.18 | **<0.001** | -0.11 | -0.28 – 0.05 | -1.35 | 0.178 | -0.50 | -0.66 – -0.34 | -6.06 | **<0.001** | -0.41 | -0.57 – -0.25 | -4.95 | **<0.001** | 0.22 | 0.07 – 0.36 | 2.91 | **0.004** |
| **Random Effects** | | | | | | | | | | | | | | | | | | | | | | | | |
| σ^2^ | 0.68 | | | | 0.63 | | | | 0.68 | | | | 0.66 | | | | 0.66 | | | | 0.54 | | | |
| τ_00_ | 0.71 _sid_ | | | | 0.68 _sid_ | | | | 0.44 _sid_ | | | | 0.56 _sid_ | | | | 0.55 _sid_ | | | | 0.73 _sid_ | | | |
|  | 0.01 _itemno_ | | | | 0.01 _itemno_ | | | | 0.02 _itemno_ | | | | 0.01 _itemno_ | | | | 0.02 _itemno_ | | | | 0.01 _itemno_ | | | |
| ICC | 0.51 | | | | 0.52 | | | | 0.40 | | | | 0.47 | | | | 0.46 | | | | 0.58 | | | |
| N | 196 _sid_ | | | | 196 _sid_ | | | | 196 _sid_ | | | | 196 _sid_ | | | | 196 _sid_ | | | | 196 _sid_ | | | |
|  | 16 _itemno_ | | | | 16 _itemno_ | | | | 16 _itemno_ | | | | 16 _itemno_ | | | | 16 _itemno_ | | | | 16 _itemno_ | | | |
| Observations | 3136 | | | | 3136 | | | | 3136 | | | | 3136 | | | | 3136 | | | | 3136 | | | |
| Marginal R^2^ / Conditional R^2^ | 0.240 / 0.631 | | | | 0.245 / 0.639 | | | | 0.194 / 0.516 | | | | 0.326 / 0.640 | | | | 0.332 / 0.639 | | | | 0.275 / 0.695 | | | |

Table S17. Beta values, confidence intervals, p-values for the *art_experience_content* model for aesthetic impacts.

|  | **angry** | | | | **calm** | | | | **compassionate** | | | | **challenging** | | | | **edified** | | | |
| --- | --- | --- | --- | --- | --- | --- | --- | --- | --- | --- | --- | --- | --- | --- | --- | --- | --- | --- | --- | --- |
| *Predictors* | *Estimates* | *CI* | *Statistic* | *p* | *Estimates* | *CI* | *Statistic* | *p* | *Estimates* | *CI* | *Statistic* | *p* | *Estimates* | *CI* | *Statistic* | *p* | *Estimates* | *CI* | *Statistic* | *p* |
| Intercept | 1.79 | 1.66 – 1.92 | 26.64 | **<0.001** | 3.34 | 3.22 – 3.46 | 54.93 | **<0.001** | 3.06 | 2.94 – 3.19 | 47.77 | **<0.001** | 2.82 | 2.70 – 2.94 | 45.32 | **<0.001** | 2.67 | 2.54 – 2.81 | 38.80 | **<0.001** |
| Content Type | 0.07 | -0.01 – 0.15 | 1.66 | 0.098 | 0.03 | -0.08 – 0.14 | 0.57 | 0.568 | 0.21 | 0.09 – 0.33 | 3.56 | **<0.001** | 0.05 | -0.05 – 0.15 | 1.04 | 0.298 | 0.11 | 0.01 – 0.21 | 2.26 | **0.024** |
| Art Experience | 0.98 | 0.68 – 1.29 | 6.40 | **<0.001** | 1.06 | 0.79 – 1.33 | 7.60 | **<0.001** | 1.31 | 1.02 – 1.60 | 8.93 | **<0.001** | 1.83 | 1.55 – 2.11 | 12.84 | **<0.001** | 1.34 | 1.03 – 1.65 | 8.51 | **<0.001** |
| Age | 0.06 | -0.07 – 0.19 | 0.88 | 0.377 | -0.07 | -0.19 – 0.05 | -1.10 | 0.271 | 0.08 | -0.04 – 0.21 | 1.27 | 0.205 | 0.09 | -0.03 – 0.21 | 1.43 | 0.153 | 0.16 | 0.03 – 0.30 | 2.34 | **0.020** |
| Education | -0.11 | -0.24 – 0.03 | -1.58 | 0.116 | -0.02 | -0.14 – 0.10 | -0.34 | 0.731 | 0.00 | -0.12 – 0.13 | 0.07 | 0.944 | -0.07 | -0.19 – 0.05 | -1.09 | 0.275 | -0.04 | -0.17 – 0.10 | -0.53 | 0.596 |
| Openness to Experience | -0.27 | -0.42 – -0.12 | -3.52 | **<0.001** | 0.02 | -0.12 – 0.15 | 0.23 | 0.815 | 0.04 | -0.11 – 0.18 | 0.48 | 0.628 | -0.10 | -0.24 – 0.04 | -1.40 | 0.162 | -0.11 | -0.27 – 0.04 | -1.43 | 0.153 |
| Content Type x Art Experience | -0.21 | -0.37 – -0.04 | -2.43 | **0.015** | 0.12 | -0.10 – 0.35 | 1.06 | 0.288 | -0.37 | -0.61 – -0.14 | -3.15 | **0.002** | -0.13 | -0.33 – 0.06 | -1.32 | 0.187 | -0.00 | -0.20 – 0.19 | -0.02 | 0.981 |
| **Random Effects** | | | | | | | | | | | | | | | | | | | | |
| σ^2^ | 0.35 | | | | 0.63 | | | | 0.69 | | | | 0.49 | | | | 0.47 | | | |
| τ_00_ | 0.78 _sid_ | | | | 0.56 _sid_ | | | | 0.63 _sid_ | | | | 0.63 _sid_ | | | | 0.80 _sid_ | | | |
| ICC | 0.69 | | | | 0.47 | | | | 0.48 | | | | 0.56 | | | | 0.63 | | | |
| N | 196 _sid_ | | | | 196 _sid_ | | | | 196 _sid_ | | | | 196 _sid_ | | | | 196 _sid_ | | | |
| Observations | 784 | | | | 784 | | | | 783 | | | | 784 | | | | 783 | | | |
| Marginal R^2^ / Conditional R^2^ | 0.286 / 0.778 | | | | 0.185 / 0.566 | | | | 0.251 / 0.608 | | | | 0.459 / 0.764 | | | | 0.310 / 0.744 | | | |

|  | **enraptured** | | | | **enlightened** | | | | **interested** | | | | **inspired** | | | | **pleasure** | | | | **upset** | | | |
| --- | --- | --- | --- | --- | --- | --- | --- | --- | --- | --- | --- | --- | --- | --- | --- | --- | --- | --- | --- | --- | --- | --- | --- | --- |
| *Predictors* | *Estimates* | *CI* | *Statistic* | *p* | *Estimates* | *CI* | *Statistic* | *p* | *Estimates* | *CI* | *Statistic* | *p* | *Estimates* | *CI* | *Statistic* | *p* | *Estimates* | *CI* | *Statistic* | *p* | *Estimates* | *CI* | *Statistic* | *p* |
| Intercept | 2.76 | 2.63 – 2.89 | 42.56 | **<0.001** | 3.02 | 2.89 – 3.15 | 46.30 | **<0.001** | 3.53 | 3.42 – 3.64 | 62.28 | **<0.001** | 3.18 | 3.06 – 3.30 | 51.52 | **<0.001** | 3.21 | 3.09 – 3.33 | 52.70 | **<0.001** | 1.87 | 1.74 – 2.00 | 28.20 | **<0.001** |
| Content Type | -0.04 | -0.15 – 0.07 | -0.74 | 0.461 | -0.00 | -0.11 – 0.11 | -0.03 | 0.974 | 0.02 | -0.09 – 0.13 | 0.34 | 0.734 | 0.02 | -0.09 – 0.14 | 0.42 | 0.673 | -0.10 | -0.21 – 0.01 | -1.75 | 0.080 | 0.12 | 0.02 – 0.22 | 2.46 | **0.014** |
| Art Experience | 1.26 | 0.97 – 1.55 | 8.47 | **<0.001** | 1.33 | 1.03 – 1.62 | 8.86 | **<0.001** | 1.00 | 0.75 – 1.26 | 7.72 | **<0.001** | 1.52 | 1.24 – 1.80 | 10.75 | **<0.001** | 1.53 | 1.26 – 1.80 | 10.96 | **<0.001** | 1.03 | 0.73 – 1.33 | 6.77 | **<0.001** |
| Age | 0.12 | -0.01 – 0.24 | 1.78 | 0.075 | 0.11 | -0.02 – 0.24 | 1.71 | 0.088 | 0.07 | -0.04 – 0.18 | 1.20 | 0.232 | -0.03 | -0.15 – 0.09 | -0.44 | 0.657 | 0.03 | -0.09 – 0.15 | 0.47 | 0.637 | 0.06 | -0.07 – 0.19 | 0.89 | 0.374 |
| Education | -0.04 | -0.17 – 0.09 | -0.65 | 0.517 | -0.02 | -0.15 – 0.11 | -0.28 | 0.776 | -0.02 | -0.13 – 0.09 | -0.33 | 0.744 | 0.01 | -0.11 – 0.14 | 0.23 | 0.821 | -0.02 | -0.14 – 0.10 | -0.39 | 0.700 | -0.09 | -0.22 – 0.04 | -1.36 | 0.175 |
| Openness to Experience | -0.11 | -0.26 – 0.03 | -1.55 | 0.123 | 0.03 | -0.11 – 0.18 | 0.44 | 0.661 | 0.04 | -0.09 – 0.17 | 0.62 | 0.538 | 0.02 | -0.12 – 0.16 | 0.28 | 0.777 | 0.03 | -0.10 – 0.17 | 0.50 | 0.618 | -0.30 | -0.45 – -0.15 | -3.92 | **<0.001** |
| Content Type x Art Experience | 0.08 | -0.14 – 0.31 | 0.74 | 0.461 | -0.13 | -0.35 – 0.09 | -1.14 | 0.253 | 0.03 | -0.19 – 0.25 | 0.24 | 0.810 | -0.10 | -0.33 – 0.13 | -0.82 | 0.413 | 0.09 | -0.14 – 0.32 | 0.78 | 0.436 | -0.24 | -0.43 – -0.05 | -2.46 | **0.014** |
| **Random Effects** | | | | | | | | | | | | | | | | | | | | | | | | |
| σ^2^ | 0.63 | | | | 0.60 | | | | 0.62 | | | | 0.67 | | | | 0.65 | | | | 0.47 | | | |
| τ_00_ | 0.66 _sid_ | | | | 0.67 _sid_ | | | | 0.47 _sid_ | | | | 0.57 _sid_ | | | | 0.56 _sid_ | | | | 0.74 _sid_ | | | |
| ICC | 0.51 | | | | 0.53 | | | | 0.43 | | | | 0.46 | | | | 0.46 | | | | 0.61 | | | |
| N | 196 _sid_ | | | | 196 _sid_ | | | | 196 _sid_ | | | | 196 _sid_ | | | | 196 _sid_ | | | | 196 _sid_ | | | |
| Observations | 784 | | | | 784 | | | | 784 | | | | 784 | | | | 784 | | | | 784 | | | |
| Marginal R^2^ / Conditional R^2^ | 0.277 / 0.645 | | | | 0.255 / 0.649 | | | | 0.181 / 0.533 | | | | 0.311 / 0.627 | | | | 0.318 / 0.633 | | | | 0.299 / 0.728 | | | |

Table S18. Beta values, confidence intervals, p-values for the *openness_experience* model.

|  | **angry** | | | | **calm** | | | | **compassionate** | | | | **challenging** | | | | **edified** | | | |
| --- | --- | --- | --- | --- | --- | --- | --- | --- | --- | --- | --- | --- | --- | --- | --- | --- | --- | --- | --- | --- |
| *Predictors* | *Estimates* | *CI* | *Statistic* | *p* | *Estimates* | *CI* | *Statistic* | *p* | *Estimates* | *CI* | *Statistic* | *p* | *Estimates* | *CI* | *Statistic* | *p* | *Estimates* | *CI* | *Statistic* | *p* |
| Intercept | 1.97 | 1.82 – 2.12 | 25.58 | **<0.001** | 3.04 | 2.86 – 3.22 | 32.96 | **<0.001** | 3.19 | 3.02 – 3.36 | 36.99 | **<0.001** | 3.06 | 2.91 – 3.21 | 40.89 | **<0.001** | 2.73 | 2.58 – 2.88 | 35.34 | **<0.001** |
| Content Information | -0.20 | -0.31 – -0.08 | -3.38 | **0.001** | 0.36 | 0.14 – 0.57 | 3.25 | **0.001** | -0.04 | -0.22 – 0.14 | -0.46 | 0.643 | -0.18 | -0.31 – -0.05 | -2.74 | **0.006** | -0.03 | -0.14 – 0.09 | -0.45 | 0.651 |
| Artist Information | -0.27 | -0.38 – -0.15 | -4.58 | **<0.001** | 0.61 | 0.39 – 0.83 | 5.54 | **<0.001** | 0.12 | -0.06 – 0.30 | 1.34 | 0.181 | -0.19 | -0.32 – -0.07 | -2.98 | **0.003** | 0.07 | -0.04 – 0.18 | 1.20 | 0.229 |
| Technique Information | -0.14 | -0.26 – -0.03 | -2.45 | **0.014** | 0.31 | 0.10 – 0.53 | 2.83 | **0.005** | 0.07 | -0.10 – 0.25 | 0.82 | 0.411 | -0.14 | -0.27 – -0.02 | -2.21 | **0.027** | 0.06 | -0.05 – 0.17 | 1.10 | 0.271 |
| Openness to Experience | -0.46 | -0.77 – -0.14 | -2.87 | **0.004** | -0.16 | -0.42 – 0.10 | -1.23 | 0.219 | 0.21 | -0.08 – 0.50 | 1.43 | 0.152 | 0.19 | -0.10 – 0.48 | 1.26 | 0.209 | -0.07 | -0.39 – 0.26 | -0.40 | 0.686 |
| Age | 0.04 | -0.08 – 0.16 | 0.62 | 0.538 | -0.02 | -0.12 – 0.08 | -0.41 | 0.682 | 0.10 | -0.01 – 0.21 | 1.72 | 0.085 | 0.08 | -0.03 – 0.19 | 1.37 | 0.172 | 0.14 | 0.01 – 0.27 | 2.17 | **0.030** |
| Education | -0.14 | -0.26 – -0.01 | -2.15 | **0.031** | -0.02 | -0.12 – 0.07 | -0.49 | 0.624 | -0.05 | -0.16 – 0.07 | -0.80 | 0.423 | -0.06 | -0.17 – 0.06 | -0.99 | 0.323 | -0.06 | -0.19 – 0.07 | -0.88 | 0.377 |
| Art Experience | 0.50 | 0.36 – 0.65 | 6.73 | **<0.001** | 0.61 | 0.50 – 0.73 | 10.48 | **<0.001** | 0.66 | 0.53 – 0.80 | 9.84 | **<0.001** | 0.88 | 0.74 – 1.01 | 12.80 | **<0.001** | 0.66 | 0.51 – 0.81 | 8.67 | **<0.001** |
| Content Information x Openness to Experience | -0.06 | -0.20 – 0.08 | -0.88 | 0.378 | 0.28 | 0.10 – 0.45 | 3.16 | **0.002** | -0.10 | -0.27 – 0.07 | -1.16 | 0.247 | -0.35 | -0.50 – -0.19 | -4.41 | **<0.001** | -0.15 | -0.31 – 0.00 | -1.96 | 0.051 |
| Artist Information x Openness to Experience | -0.09 | -0.22 – 0.05 | -1.23 | 0.219 | 0.60 | 0.42 – 0.77 | 6.80 | **<0.001** | 0.09 | -0.08 – 0.26 | 0.99 | 0.323 | -0.28 | -0.43 – -0.12 | -3.49 | **<0.001** | -0.03 | -0.18 – 0.13 | -0.35 | 0.730 |
| Technique Information x Openness to Experience | -0.12 | -0.25 – 0.02 | -1.67 | 0.096 | 0.26 | 0.09 – 0.43 | 2.97 | **0.003** | 0.15 | -0.02 – 0.32 | 1.75 | 0.080 | -0.21 | -0.37 – -0.06 | -2.68 | **0.007** | 0.07 | -0.08 – 0.23 | 0.93 | 0.352 |
| **Random Effects** | | | | | | | | | | | | | | | | | | | | |
| σ^2^ | 0.45 | | | | 0.71 | | | | 0.70 | | | | 0.58 | | | | 0.57 | | | |
| τ_00_ | 0.75 _sid_ | | | | 0.44 _sid_ | | | | 0.60 _sid_ | | | | 0.63 _sid_ | | | | 0.78 _sid_ | | | |
|  | 0.00 _itemno_ | | | | 0.02 _itemno_ | | | | 0.01 _itemno_ | | | | 0.01 _itemno_ | | | | 0.00 _itemno_ | | | |
| ICC | 0.63 | | | | 0.39 | | | | 0.46 | | | | 0.52 | | | | 0.58 | | | |
| N | 198 _sid_ | | | | 198 _sid_ | | | | 198 _sid_ | | | | 198 _sid_ | | | | 198 _sid_ | | | |
|  | 16 _itemno_ | | | | 16 _itemno_ | | | | 16 _itemno_ | | | | 16 _itemno_ | | | | 16 _itemno_ | | | |
| Observations | 3168 | | | | 3168 | | | | 3167 | | | | 3168 | | | | 3167 | | | |
| Marginal R^2^ / Conditional R^2^ | 0.279 / 0.732 | | | | 0.249 / 0.545 | | | | 0.230 / 0.588 | | | | 0.397 / 0.712 | | | | 0.269 / 0.692 | | | |

|  | **enraptured** | | | | **enlightened** | | | | **interested** | | | | **inspired** | | | | **pleasure** | | | | **upset** | | | |
| --- | --- | --- | --- | --- | --- | --- | --- | --- | --- | --- | --- | --- | --- | --- | --- | --- | --- | --- | --- | --- | --- | --- | --- | --- |
| *Predictors* | *Estimates* | *CI* | *Statistic* | *p* | *Estimates* | *CI* | *Statistic* | *p* | *Estimates* | *CI* | *Statistic* | *p* | *Estimates* | *CI* | *Statistic* | *p* | *Estimates* | *CI* | *Statistic* | *p* | *Estimates* | *CI* | *Statistic* | *p* |
| Intercept | 2.86 | 2.69 – 3.03 | 33.47 | **<0.001** | 2.94 | 2.79 – 3.10 | 36.99 | **<0.001** | 3.47 | 3.30 – 3.64 | 40.50 | **<0.001** | 3.09 | 2.92 – 3.25 | 37.30 | **<0.001** | 2.97 | 2.79 – 3.14 | 33.30 | **<0.001** | 2.15 | 1.97 – 2.33 | 23.43 | **<0.001** |
| Content Information | -0.06 | -0.22 – 0.11 | -0.69 | 0.493 | 0.16 | 0.01 – 0.31 | 2.10 | **0.036** | 0.12 | -0.08 – 0.32 | 1.19 | 0.233 | 0.18 | 0.00 – 0.36 | 2.02 | **0.044** | 0.33 | 0.13 – 0.54 | 3.23 | **0.001** | -0.31 | -0.50 – -0.13 | -3.39 | **0.001** |
| Artist Information | 0.15 | -0.02 – 0.31 | 1.74 | 0.082 | 0.23 | 0.08 – 0.38 | 3.03 | **0.002** | 0.23 | 0.04 – 0.43 | 2.34 | **0.019** | 0.36 | 0.19 – 0.54 | 4.01 | **<0.001** | 0.58 | 0.38 – 0.78 | 5.61 | **<0.001** | -0.40 | -0.58 – -0.21 | -4.27 | **<0.001** |
| Technique Information | 0.06 | -0.11 – 0.22 | 0.66 | 0.509 | 0.17 | 0.02 – 0.32 | 2.22 | **0.027** | 0.12 | -0.07 – 0.32 | 1.22 | 0.224 | 0.24 | 0.06 – 0.42 | 2.64 | **0.008** | 0.31 | 0.10 – 0.51 | 2.94 | **0.003** | -0.27 | -0.45 – -0.09 | -2.89 | **0.004** |
| Openness to Experience | -0.06 | -0.36 – 0.24 | -0.40 | 0.692 | 0.17 | -0.13 – 0.46 | 1.11 | 0.266 | 0.27 | 0.02 – 0.53 | 2.11 | **0.035** | -0.00 | -0.27 – 0.27 | -0.01 | 0.994 | -0.03 | -0.30 – 0.23 | -0.25 | 0.800 | -0.44 | -0.75 – -0.13 | -2.75 | **0.006** |
| Age | 0.10 | -0.02 – 0.21 | 1.63 | 0.104 | 0.09 | -0.02 – 0.21 | 1.58 | 0.115 | 0.08 | -0.02 – 0.17 | 1.62 | 0.105 | -0.02 | -0.13 – 0.08 | -0.48 | 0.631 | 0.02 | -0.08 – 0.12 | 0.47 | 0.641 | 0.04 | -0.09 – 0.16 | 0.60 | 0.548 |
| Education | -0.06 | -0.18 – 0.05 | -1.06 | 0.291 | -0.05 | -0.16 – 0.07 | -0.81 | 0.420 | 0.01 | -0.08 – 0.11 | 0.25 | 0.799 | -0.02 | -0.13 – 0.08 | -0.43 | 0.664 | -0.04 | -0.14 – 0.06 | -0.83 | 0.406 | -0.13 | -0.25 – -0.00 | -2.03 | **0.043** |
| Art Experience | 0.66 | 0.52 – 0.79 | 9.26 | **<0.001** | 0.72 | 0.58 – 0.85 | 10.42 | **<0.001** | 0.57 | 0.45 – 0.68 | 9.89 | **<0.001** | 0.84 | 0.72 – 0.96 | 13.76 | **<0.001** | 0.85 | 0.74 – 0.97 | 14.27 | **<0.001** | 0.50 | 0.35 – 0.64 | 6.69 | **<0.001** |
| Content Information x Openness to Experience | -0.12 | -0.28 – 0.05 | -1.36 | 0.175 | -0.04 | -0.21 – 0.12 | -0.52 | 0.605 | -0.12 | -0.29 – 0.05 | -1.41 | 0.157 | 0.11 | -0.05 – 0.28 | 1.34 | 0.180 | 0.21 | 0.05 – 0.38 | 2.51 | **0.012** | -0.21 | -0.36 – -0.06 | -2.71 | **0.007** |
| Artist Information x Openness to Experience | 0.07 | -0.09 – 0.24 | 0.87 | 0.383 | 0.09 | -0.07 – 0.25 | 1.08 | 0.279 | 0.09 | -0.08 – 0.26 | 1.01 | 0.310 | 0.33 | 0.16 – 0.49 | 3.86 | **<0.001** | 0.64 | 0.47 – 0.81 | 7.51 | **<0.001** | -0.17 | -0.32 – -0.02 | -2.18 | **0.029** |
| Technique Information x Openness to Experience | 0.00 | -0.16 – 0.17 | 0.05 | 0.961 | 0.13 | -0.03 – 0.29 | 1.58 | 0.115 | 0.00 | -0.17 – 0.17 | 0.04 | 0.970 | 0.35 | 0.18 – 0.52 | 4.13 | **<0.001** | 0.25 | 0.08 – 0.41 | 2.89 | **0.004** | -0.05 | -0.20 – 0.10 | -0.68 | 0.494 |
| **Random Effects** | | | | | | | | | | | | | | | | | | | | | | | | |
| σ^2^ | 0.68 | | | | 0.64 | | | | 0.68 | | | | 0.66 | | | | 0.67 | | | | 0.54 | | | |
| τ_00_ | 0.66 _sid_ | | | | 0.63 _sid_ | | | | 0.42 _sid_ | | | | 0.49 _sid_ | | | | 0.46 _sid_ | | | | 0.75 _sid_ | | | |
|  | 0.01 _itemno_ | | | | 0.01 _itemno_ | | | | 0.02 _itemno_ | | | | 0.01 _itemno_ | | | | 0.02 _itemno_ | | | | 0.01 _itemno_ | | | |
| ICC | 0.50 | | | | 0.50 | | | | 0.39 | | | | 0.43 | | | | 0.42 | | | | 0.58 | | | |
| N | 198 _sid_ | | | | 198 _sid_ | | | | 198 _sid_ | | | | 198 _sid_ | | | | 198 _sid_ | | | | 198 _sid_ | | | |
|  | 16 _itemno_ | | | | 16 _itemno_ | | | | 16 _itemno_ | | | | 16 _itemno_ | | | | 16 _itemno_ | | | | 16 _itemno_ | | | |
| Observations | 3168 | | | | 3168 | | | | 3168 | | | | 3168 | | | | 3168 | | | | 3168 | | | |
| Marginal R^2^ / Conditional R^2^ | 0.259 / 0.627 | | | | 0.269 / 0.634 | | | | 0.203 / 0.513 | | | | 0.358 / 0.634 | | | | 0.372 / 0.634 | | | | 0.271 / 0.696 | | | |

Table S19. Beta values, confidence intervals, p-values for the *openness_experience_content* model.

|  | **angry** | | | | **calm** | | | | **compassionate** | | | | **challenging** | | | | **edified** | | | |
| --- | --- | --- | --- | --- | --- | --- | --- | --- | --- | --- | --- | --- | --- | --- | --- | --- | --- | --- | --- | --- |
| *Predictors* | *Estimates* | *CI* | *Statistic* | *p* | *Estimates* | *CI* | *Statistic* | *p* | *Estimates* | *CI* | *Statistic* | *p* | *Estimates* | *CI* | *Statistic* | *p* | *Estimates* | *CI* | *Statistic* | *p* |
| Intercept | 1.77 | 1.63 – 1.91 | 25.25 | **<0.001** | 3.40 | 3.28 – 3.52 | 56.06 | **<0.001** | 3.15 | 3.02 – 3.28 | 48.37 | **<0.001** | 2.89 | 2.76 – 3.02 | 43.87 | **<0.001** | 2.71 | 2.56 – 2.85 | 37.53 | **<0.001** |
| Content Type | 0.08 | -0.01 – 0.17 | 1.73 | 0.084 | 0.04 | -0.08 – 0.15 | 0.60 | 0.549 | 0.25 | 0.13 – 0.37 | 4.10 | **<0.001** | 0.06 | -0.04 – 0.16 | 1.23 | 0.220 | 0.11 | 0.01 – 0.21 | 2.20 | **0.028** |
| Openness to Experience | -0.53 | -0.85 – -0.21 | -3.27 | **0.001** | 0.08 | -0.19 – 0.36 | 0.59 | 0.552 | 0.14 | -0.15 – 0.44 | 0.95 | 0.342 | -0.11 | -0.41 – 0.19 | -0.74 | 0.459 | -0.23 | -0.56 – 0.10 | -1.38 | 0.167 |
| Age | 0.06 | -0.07 – 0.19 | 0.88 | 0.379 | -0.08 | -0.20 – 0.03 | -1.42 | 0.156 | 0.08 | -0.04 – 0.20 | 1.32 | 0.188 | 0.08 | -0.04 – 0.20 | 1.29 | 0.197 | 0.15 | 0.01 – 0.28 | 2.12 | **0.035** |
| Education | -0.13 | -0.26 – 0.00 | -1.91 | 0.057 | -0.05 | -0.16 – 0.07 | -0.83 | 0.408 | -0.02 | -0.15 – 0.10 | -0.37 | 0.708 | -0.10 | -0.23 – 0.02 | -1.60 | 0.109 | -0.06 | -0.20 – 0.07 | -0.92 | 0.357 |
| Art Experience | 0.49 | 0.33 – 0.64 | 6.17 | **<0.001** | 0.59 | 0.45 – 0.72 | 8.58 | **<0.001** | 0.70 | 0.55 – 0.84 | 9.47 | **<0.001** | 0.92 | 0.77 – 1.07 | 12.35 | **<0.001** | 0.65 | 0.49 – 0.81 | 7.98 | **<0.001** |
| Content Type x Openness to Experience | 0.15 | -0.03 – 0.32 | 1.64 | 0.101 | -0.10 | -0.33 – 0.13 | -0.83 | 0.405 | 0.43 | 0.19 – 0.67 | 3.56 | **<0.001** | 0.13 | -0.07 – 0.34 | 1.30 | 0.192 | 0.12 | -0.08 – 0.32 | 1.14 | 0.254 |
| **Random Effects** | | | | | | | | | | | | | | | | | | | | |
| σ^2^ | 0.37 | | | | 0.63 | | | | 0.68 | | | | 0.49 | | | | 0.48 | | | |
| τ_00_ | 0.79 _sid_ | | | | 0.50 _sid_ | | | | 0.59 _sid_ | | | | 0.66 _sid_ | | | | 0.81 _sid_ | | | |
| ICC | 0.68 | | | | 0.44 | | | | 0.46 | | | | 0.57 | | | | 0.63 | | | |
| N | 198 _sid_ | | | | 198 _sid_ | | | | 198 _sid_ | | | | 198 _sid_ | | | | 198 _sid_ | | | |
| Observations | 792 | | | | 792 | | | | 791 | | | | 792 | | | | 791 | | | |
| Marginal R^2^ / Conditional R^2^ | 0.280 / 0.771 | | | | 0.220 / 0.564 | | | | 0.271 / 0.610 | | | | 0.443 / 0.763 | | | | 0.295 / 0.737 | | | |

|  | **enraptured** | | | | **enlightened** | | | | **interested** | | | | **inspired** | | | | **pleasure** | | | | **upset** | | | |
| --- | --- | --- | --- | --- | --- | --- | --- | --- | --- | --- | --- | --- | --- | --- | --- | --- | --- | --- | --- | --- | --- | --- | --- | --- |
| *Predictors* | *Estimates* | *CI* | *Statistic* | *p* | *Estimates* | *CI* | *Statistic* | *p* | *Estimates* | *CI* | *Statistic* | *p* | *Estimates* | *CI* | *Statistic* | *p* | *Estimates* | *CI* | *Statistic* | *p* | *Estimates* | *CI* | *Statistic* | *p* |
| Intercept | 2.80 | 2.67 – 2.93 | 42.72 | **<0.001** | 3.10 | 2.98 – 3.23 | 47.74 | **<0.001** | 3.58 | 3.47 – 3.70 | 62.19 | **<0.001** | 3.27 | 3.15 – 3.38 | 54.21 | **<0.001** | 3.30 | 3.19 – 3.42 | 56.46 | **<0.001** | 1.84 | 1.71 – 1.98 | 26.60 | **<0.001** |
| Content Type | -0.05 | -0.16 – 0.07 | -0.77 | 0.441 | 0.02 | -0.09 – 0.13 | 0.36 | 0.721 | 0.03 | -0.09 – 0.14 | 0.48 | 0.634 | 0.03 | -0.09 – 0.15 | 0.50 | 0.620 | -0.11 | -0.23 – 0.00 | -1.93 | 0.053 | 0.12 | 0.02 – 0.22 | 2.44 | **0.015** |
| Openness to Experience | -0.16 | -0.46 – 0.14 | -1.03 | 0.304 | 0.13 | -0.17 – 0.43 | 0.86 | 0.387 | 0.11 | -0.15 – 0.38 | 0.84 | 0.399 | 0.09 | -0.19 – 0.36 | 0.63 | 0.526 | 0.18 | -0.09 – 0.45 | 1.33 | 0.183 | -0.64 | -0.96 – -0.33 | -3.99 | **<0.001** |
| Age | 0.10 | -0.02 – 0.23 | 1.67 | 0.095 | 0.11 | -0.02 – 0.23 | 1.69 | 0.091 | 0.06 | -0.05 – 0.16 | 1.02 | 0.310 | -0.04 | -0.15 – 0.07 | -0.66 | 0.509 | 0.01 | -0.10 – 0.12 | 0.20 | 0.839 | 0.06 | -0.07 – 0.19 | 0.95 | 0.341 |
| Education | -0.08 | -0.20 – 0.05 | -1.21 | 0.226 | -0.05 | -0.17 – 0.07 | -0.79 | 0.428 | -0.04 | -0.15 – 0.07 | -0.69 | 0.491 | -0.02 | -0.14 – 0.09 | -0.36 | 0.717 | -0.06 | -0.17 – 0.05 | -1.11 | 0.269 | -0.11 | -0.24 – 0.02 | -1.66 | 0.098 |
| Art Experience | 0.67 | 0.53 – 0.82 | 9.09 | **<0.001** | 0.72 | 0.58 – 0.87 | 9.82 | **<0.001** | 0.53 | 0.40 – 0.66 | 8.13 | **<0.001** | 0.82 | 0.69 – 0.96 | 12.09 | **<0.001** | 0.85 | 0.72 – 0.98 | 12.90 | **<0.001** | 0.50 | 0.35 – 0.66 | 6.44 | **<0.001** |
| Content Type x Openness to Experience | -0.07 | -0.30 – 0.16 | -0.63 | 0.526 | 0.14 | -0.09 – 0.36 | 1.19 | 0.233 | 0.10 | -0.13 – 0.32 | 0.82 | 0.411 | 0.13 | -0.11 – 0.37 | 1.09 | 0.275 | -0.14 | -0.37 – 0.09 | -1.19 | 0.234 | 0.11 | -0.09 – 0.31 | 1.09 | 0.275 |
| **Random Effects** | | | | | | | | | | | | | | | | | | | | | | | | |
| σ^2^ | 0.63 | | | | 0.61 | | | | 0.62 | | | | 0.67 | | | | 0.65 | | | | 0.47 | | | |
| τ_00_ | 0.62 _sid_ | | | | 0.61 _sid_ | | | | 0.44 _sid_ | | | | 0.48 _sid_ | | | | 0.45 _sid_ | | | | 0.74 _sid_ | | | |
| ICC | 0.49 | | | | 0.50 | | | | 0.42 | | | | 0.42 | | | | 0.41 | | | | 0.61 | | | |
| N | 198 _sid_ | | | | 198 _sid_ | | | | 198 _sid_ | | | | 198 _sid_ | | | | 198 _sid_ | | | | 198 _sid_ | | | |
| Observations | 792 | | | | 792 | | | | 792 | | | | 792 | | | | 792 | | | | 792 | | | |
| Marginal R^2^ / Conditional R^2^ | 0.298 / 0.645 | | | | 0.289 / 0.645 | | | | 0.195 / 0.530 | | | | 0.356 / 0.625 | | | | 0.373 / 0.632 | | | | 0.303 / 0.730 | | | |

Table S20. Beta values, confidence intervals, p-values for the *artwork_culture* model for aesthetic impacts.

|  | **angry** | | | | **calm** | | | | **compassionate** | | | | **challenging** | | | | **edified** | | | |
| --- | --- | --- | --- | --- | --- | --- | --- | --- | --- | --- | --- | --- | --- | --- | --- | --- | --- | --- | --- | --- |
| *Predictors* | *Estimates* | *CI* | *Statistic* | *p* | *Estimates* | *CI* | *Statistic* | *p* | *Estimates* | *CI* | *Statistic* | *p* | *Estimates* | *CI* | *Statistic* | *p* | *Estimates* | *CI* | *Statistic* | *p* |
| Intercept | 2.02 | 1.88 – 2.16 | 28.24 | **<0.001** | 3.06 | 2.89 – 3.23 | 35.62 | **<0.001** | 3.16 | 3.02 – 3.31 | 43.21 | **<0.001** | 3.04 | 2.91 – 3.17 | 44.76 | **<0.001** | 2.74 | 2.60 – 2.88 | 38.55 | **<0.001** |
| Content Information | -0.18 | -0.29 – -0.08 | -3.53 | **<0.001** | 0.33 | 0.13 – 0.53 | 3.27 | **0.001** | -0.04 | -0.17 – 0.09 | -0.55 | 0.584 | -0.13 | -0.24 – -0.03 | -2.47 | **0.013** | -0.00 | -0.09 – 0.09 | -0.10 | 0.924 |
| Artist Information | -0.25 | -0.35 – -0.14 | -4.72 | **<0.001** | 0.53 | 0.34 – 0.73 | 5.28 | **<0.001** | 0.10 | -0.03 – 0.23 | 1.46 | 0.144 | -0.16 | -0.27 – -0.06 | -3.07 | **0.002** | 0.07 | -0.02 – 0.16 | 1.48 | 0.140 |
| Technique Information | -0.12 | -0.23 – -0.02 | -2.34 | **0.019** | 0.28 | 0.09 – 0.48 | 2.81 | **0.005** | 0.05 | -0.08 – 0.18 | 0.76 | 0.448 | -0.12 | -0.23 – -0.02 | -2.33 | **0.020** | 0.05 | -0.04 – 0.14 | 1.06 | 0.290 |
| Artwork Culture | -0.12 | -0.27 – 0.02 | -1.69 | 0.092 | 0.05 | -0.23 – 0.33 | 0.32 | 0.748 | -0.14 | -0.33 – 0.05 | -1.48 | 0.140 | 0.08 | -0.07 – 0.23 | 1.08 | 0.282 | 0.02 | -0.10 – 0.15 | 0.35 | 0.723 |
| Age | 0.04 | -0.09 – 0.16 | 0.56 | 0.576 | -0.02 | -0.12 – 0.08 | -0.43 | 0.666 | 0.10 | -0.02 – 0.21 | 1.65 | 0.099 | 0.09 | -0.03 – 0.20 | 1.46 | 0.146 | 0.15 | 0.02 – 0.27 | 2.23 | **0.026** |
| Education | -0.13 | -0.25 – -0.01 | -2.06 | **0.039** | -0.03 | -0.13 – 0.07 | -0.53 | 0.596 | -0.04 | -0.16 – 0.07 | -0.73 | 0.465 | -0.05 | -0.16 – 0.07 | -0.78 | 0.438 | -0.05 | -0.18 – 0.08 | -0.77 | 0.439 |
| Art Experience | 0.50 | 0.37 – 0.64 | 7.17 | **<0.001** | 0.61 | 0.50 – 0.72 | 10.89 | **<0.001** | 0.64 | 0.51 – 0.76 | 9.78 | **<0.001** | 0.84 | 0.71 – 0.96 | 12.79 | **<0.001** | 0.65 | 0.51 – 0.80 | 8.95 | **<0.001** |
| Openness to Experience | -0.28 | -0.42 – -0.14 | -3.99 | **<0.001** | 0.06 | -0.05 – 0.17 | 1.12 | 0.261 | 0.08 | -0.05 – 0.21 | 1.20 | 0.229 | -0.11 | -0.23 – 0.02 | -1.62 | 0.105 | -0.07 | -0.21 – 0.07 | -0.99 | 0.323 |
| Content Information x Artwork Culture | 0.08 | -0.13 – 0.28 | 0.75 | 0.450 | 0.03 | -0.36 – 0.43 | 0.16 | 0.870 | -0.13 | -0.39 – 0.13 | -0.96 | 0.335 | -0.20 | -0.41 – 0.01 | -1.86 | 0.064 | 0.03 | -0.15 – 0.21 | 0.29 | 0.768 |
| Artist Information x Artwork Culture | 0.15 | -0.05 – 0.36 | 1.46 | 0.144 | -0.02 | -0.42 – 0.37 | -0.11 | 0.910 | -0.03 | -0.29 – 0.24 | -0.21 | 0.835 | 0.02 | -0.19 – 0.23 | 0.17 | 0.868 | -0.10 | -0.28 – 0.08 | -1.09 | 0.278 |
| Technique Information x Artwork Culture | 0.09 | -0.12 – 0.29 | 0.83 | 0.408 | 0.16 | -0.24 – 0.56 | 0.79 | 0.427 | 0.15 | -0.11 – 0.42 | 1.14 | 0.255 | -0.01 | -0.22 – 0.21 | -0.05 | 0.962 | 0.13 | -0.05 – 0.31 | 1.39 | 0.164 |
| **Random Effects** | | | | | | | | | | | | | | | | | | | | |
| σ^2^ | 0.44 | | | | 0.72 | | | | 0.70 | | | | 0.58 | | | | 0.57 | | | |
| τ_00_ | 0.73 _sid_ | | | | 0.44 _sid_ | | | | 0.61 _sid_ | | | | 0.62 _sid_ | | | | 0.79 _sid_ | | | |
|  | 0.00 _itemno_ | | | | 0.02 _itemno_ | | | | 0.01 _itemno_ | | | | 0.00 _itemno_ | | | | 0.00 _itemno_ | | | |
| ICC | 0.62 | | | | 0.39 | | | | 0.47 | | | | 0.52 | | | | 0.58 | | | |
| N | 196 _sid_ | | | | 196 _sid_ | | | | 196 _sid_ | | | | 196 _sid_ | | | | 196 _sid_ | | | |
|  | 16 _itemno_ | | | | 16 _itemno_ | | | | 16 _itemno_ | | | | 16 _itemno_ | | | | 16 _itemno_ | | | |
| Observations | 3136 | | | | 3136 | | | | 3135 | | | | 3136 | | | | 3135 | | | |
| Marginal R^2^ / Conditional R^2^ | 0.288 / 0.732 | | | | 0.246 / 0.541 | | | | 0.230 / 0.589 | | | | 0.402 / 0.713 | | | | 0.272 / 0.695 | | | |

|  | **enraptured** | | | | **enlightened** | | | | **interested** | | | | **inspired** | | | | **pleasure** | | | | **upset** | | | |
| --- | --- | --- | --- | --- | --- | --- | --- | --- | --- | --- | --- | --- | --- | --- | --- | --- | --- | --- | --- | --- | --- | --- | --- | --- |
| *Predictors* | *Estimates* | *CI* | *Statistic* | *p* | *Estimates* | *CI* | *Statistic* | *p* | *Estimates* | *CI* | *Statistic* | *p* | *Estimates* | *CI* | *Statistic* | *p* | *Estimates* | *CI* | *Statistic* | *p* | *Estimates* | *CI* | *Statistic* | *p* |
| Intercept | 2.87 | 2.73 – 3.01 | 38.90 | **<0.001** | 2.93 | 2.79 – 3.06 | 42.52 | **<0.001** | 3.44 | 3.31 – 3.57 | 52.15 | **<0.001** | 3.09 | 2.95 – 3.22 | 43.80 | **<0.001** | 2.97 | 2.83 – 3.11 | 40.91 | **<0.001** | 2.20 | 2.03 – 2.38 | 25.09 | **<0.001** |
| Content Information | -0.05 | -0.18 – 0.08 | -0.78 | 0.438 | 0.16 | 0.05 – 0.27 | 2.87 | **0.004** | 0.14 | 0.01 – 0.27 | 2.09 | **0.037** | 0.17 | 0.03 – 0.31 | 2.40 | **0.016** | 0.31 | 0.16 – 0.46 | 4.11 | **<0.001** | -0.28 | -0.46 – -0.11 | -3.19 | **0.001** |
| Artist Information | 0.13 | 0.01 – 0.26 | 2.07 | **0.039** | 0.22 | 0.11 – 0.32 | 3.92 | **<0.001** | 0.22 | 0.09 – 0.35 | 3.36 | **0.001** | 0.32 | 0.19 – 0.46 | 4.61 | **<0.001** | 0.50 | 0.35 – 0.65 | 6.62 | **<0.001** | -0.37 | -0.54 – -0.19 | -4.13 | **<0.001** |
| Technique Information | 0.06 | -0.07 – 0.18 | 0.89 | 0.371 | 0.15 | 0.04 – 0.26 | 2.74 | **0.006** | 0.12 | -0.01 – 0.25 | 1.86 | 0.063 | 0.19 | 0.06 – 0.33 | 2.77 | **0.006** | 0.28 | 0.13 – 0.43 | 3.64 | **<0.001** | -0.26 | -0.43 – -0.08 | -2.86 | **0.004** |
| Artwork Culture | 0.05 | -0.13 – 0.23 | 0.56 | 0.574 | 0.03 | -0.12 – 0.18 | 0.36 | 0.718 | 0.09 | -0.10 – 0.27 | 0.95 | 0.342 | 0.05 | -0.15 – 0.24 | 0.46 | 0.643 | 0.05 | -0.16 – 0.26 | 0.50 | 0.619 | -0.02 | -0.26 – 0.23 | -0.12 | 0.904 |
| Age | 0.11 | -0.01 – 0.22 | 1.74 | 0.082 | 0.09 | -0.02 – 0.21 | 1.57 | 0.118 | 0.08 | -0.02 – 0.18 | 1.63 | 0.102 | -0.02 | -0.13 – 0.08 | -0.45 | 0.651 | 0.03 | -0.07 – 0.13 | 0.54 | 0.589 | 0.04 | -0.09 – 0.16 | 0.58 | 0.565 |
| Education | -0.06 | -0.18 – 0.06 | -0.92 | 0.356 | -0.05 | -0.16 – 0.07 | -0.81 | 0.416 | 0.01 | -0.09 – 0.10 | 0.15 | 0.880 | -0.02 | -0.13 – 0.08 | -0.44 | 0.662 | -0.04 | -0.14 – 0.06 | -0.78 | 0.434 | -0.12 | -0.25 – 0.00 | -1.94 | 0.052 |
| Art Experience | 0.64 | 0.51 – 0.77 | 9.46 | **<0.001** | 0.70 | 0.58 – 0.83 | 10.67 | **<0.001** | 0.56 | 0.46 – 0.67 | 10.33 | **<0.001** | 0.84 | 0.72 – 0.95 | 14.18 | **<0.001** | 0.83 | 0.72 – 0.94 | 14.46 | **<0.001** | 0.51 | 0.37 – 0.65 | 7.19 | **<0.001** |
| Openness to Experience | -0.08 | -0.21 – 0.06 | -1.14 | 0.255 | 0.09 | -0.04 – 0.22 | 1.37 | 0.172 | 0.14 | 0.03 – 0.24 | 2.53 | **0.012** | 0.09 | -0.03 – 0.20 | 1.48 | 0.138 | 0.08 | -0.03 – 0.19 | 1.44 | 0.149 | -0.28 | -0.42 – -0.14 | -3.97 | **<0.001** |
| Content Information x Artwork Culture | -0.16 | -0.41 – 0.09 | -1.25 | 0.210 | -0.05 | -0.27 – 0.16 | -0.46 | 0.643 | -0.06 | -0.32 – 0.20 | -0.44 | 0.659 | -0.03 | -0.31 – 0.24 | -0.22 | 0.827 | -0.02 | -0.32 – 0.28 | -0.12 | 0.907 | 0.01 | -0.34 – 0.36 | 0.04 | 0.966 |
| Artist Information x Artwork Culture | -0.13 | -0.38 – 0.12 | -1.03 | 0.301 | -0.10 | -0.32 – 0.11 | -0.95 | 0.342 | -0.20 | -0.46 – 0.06 | -1.52 | 0.130 | -0.14 | -0.41 – 0.14 | -0.97 | 0.334 | -0.15 | -0.44 – 0.15 | -0.96 | 0.339 | 0.09 | -0.26 – 0.44 | 0.53 | 0.597 |
| Technique Information x Artwork Culture | 0.24 | -0.01 – 0.49 | 1.85 | 0.065 | 0.24 | 0.02 – 0.46 | 2.18 | **0.029** | 0.30 | 0.04 – 0.56 | 2.28 | **0.023** | 0.26 | -0.01 – 0.53 | 1.86 | 0.063 | 0.33 | 0.04 – 0.63 | 2.20 | **0.028** | -0.12 | -0.47 – 0.23 | -0.66 | 0.511 |
| **Random Effects** | | | | | | | | | | | | | | | | | | | | | | | | |
| σ^2^ | 0.68 | | | | 0.63 | | | | 0.69 | | | | 0.67 | | | | 0.68 | | | | 0.54 | | | |
| τ_00_ | 0.66 _sid_ | | | | 0.63 _sid_ | | | | 0.42 _sid_ | | | | 0.49 _sid_ | | | | 0.47 _sid_ | | | | 0.73 _sid_ | | | |
|  | 0.00 _itemno_ | | | | 0.00 _itemno_ | | | | 0.01 _itemno_ | | | | 0.01 _itemno_ | | | | 0.01 _itemno_ | | | | 0.01 _itemno_ | | | |
| ICC | 0.50 | | | | 0.50 | | | | 0.38 | | | | 0.43 | | | | 0.41 | | | | 0.58 | | | |
| N | 196 _sid_ | | | | 196 _sid_ | | | | 196 _sid_ | | | | 196 _sid_ | | | | 196 _sid_ | | | | 196 _sid_ | | | |
|  | 16 _itemno_ | | | | 16 _itemno_ | | | | 16 _itemno_ | | | | 16 _itemno_ | | | | 16 _itemno_ | | | | 16 _itemno_ | | | |
| Observations | 3136 | | | | 3136 | | | | 3136 | | | | 3136 | | | | 3136 | | | | 3136 | | | |
| Marginal R^2^ / Conditional R^2^ | 0.265 / 0.629 | | | | 0.272 / 0.636 | | | | 0.214 / 0.514 | | | | 0.361 / 0.634 | | | | 0.371 / 0.629 | | | | 0.275 / 0.694 | | | |

Table S21. Beta values, confidence intervals, p-values for *motion* models for aesthetic impacts.

|  | **angry** | | | | **calm** | | | | **compassionate** | | | | **challenging** | | | | **edified** | | | |
| --- | --- | --- | --- | --- | --- | --- | --- | --- | --- | --- | --- | --- | --- | --- | --- | --- | --- | --- | --- | --- |
| *Predictors* | *Estimates* | *CI* | *Statistic* | *p* | *Estimates* | *CI* | *Statistic* | *p* | *Estimates* | *CI* | *Statistic* | *p* | *Estimates* | *CI* | *Statistic* | *p* | *Estimates* | *CI* | *Statistic* | *p* |
| (Intercept) | 2.03 | 1.87 – 2.19 | 24.98 | **<0.001** | 3.06 | 2.91 – 3.22 | 38.29 | **<0.001** | 3.16 | 3.00 – 3.32 | 39.88 | **<0.001** | 3.04 | 2.86 – 3.21 | 34.48 | **<0.001** | 2.74 | 2.57 – 2.91 | 31.90 | **<0.001** |
| Content Information | -0.17 | -0.24 – -0.10 | -4.61 | **<0.001** | 0.32 | 0.17 – 0.47 | 4.18 | **<0.001** | 0.06 | -0.05 – 0.17 | 1.02 | 0.309 | -0.10 | -0.18 – -0.02 | -2.35 | **0.019** | 0.01 | -0.09 – 0.10 | 0.10 | 0.920 |
| Artist Information | -0.26 | -0.32 – -0.19 | -7.66 | **<0.001** | 0.53 | 0.39 – 0.67 | 7.54 | **<0.001** | 0.11 | 0.01 – 0.21 | 2.06 | **0.039** | -0.16 | -0.23 – -0.08 | -4.13 | **<0.001** | 0.07 | -0.02 – 0.16 | 1.54 | 0.125 |
| Technique Information | -0.13 | -0.20 – -0.06 | -3.66 | **<0.001** | 0.24 | 0.09 – 0.39 | 3.10 | **0.002** | 0.06 | -0.06 – 0.17 | 0.96 | 0.339 | -0.08 | -0.16 – -0.00 | -1.97 | **0.049** | 0.08 | -0.02 – 0.18 | 1.57 | 0.116 |
| Motion | 0.26 | 0.16 – 0.35 | 5.42 | **<0.001** | -0.44 | -0.64 – -0.25 | -4.46 | **<0.001** | 0.13 | -0.02 – 0.28 | 1.71 | 0.088 | 0.22 | 0.12 – 0.33 | 4.15 | **<0.001** | -0.08 | -0.21 – 0.05 | -1.18 | 0.238 |
| Age | 0.08 | -0.07 – 0.24 | 1.05 | 0.294 | 0.02 | -0.10 – 0.15 | 0.34 | 0.737 | 0.14 | 0.00 – 0.28 | 1.99 | **0.046** | 0.14 | -0.02 – 0.31 | 1.67 | 0.095 | 0.19 | 0.03 – 0.35 | 2.34 | **0.019** |
| Education | -0.09 | -0.24 – 0.06 | -1.14 | 0.255 | 0.05 | -0.08 – 0.18 | 0.76 | 0.446 | 0.04 | -0.10 – 0.18 | 0.50 | 0.617 | 0.04 | -0.12 – 0.21 | 0.51 | 0.610 | 0.02 | -0.14 – 0.17 | 0.21 | 0.831 |
| Content Information x Motion | -0.35 | -0.49 – -0.21 | -4.82 | **<0.001** | 0.46 | 0.16 – 0.76 | 3.02 | **0.003** | -0.48 | -0.70 – -0.25 | -4.15 | **<0.001** | -0.37 | -0.53 – -0.20 | -4.43 | **<0.001** | 0.04 | -0.16 – 0.23 | 0.35 | 0.727 |
| Artist Information x Motion | -0.25 | -0.38 – -0.12 | -3.68 | **<0.001** | 0.42 | 0.14 – 0.69 | 2.95 | **0.003** | 0.10 | -0.11 – 0.31 | 0.92 | 0.356 | -0.14 | -0.29 – 0.01 | -1.82 | 0.070 | 0.19 | 0.01 – 0.37 | 2.02 | **0.043** |
| Technique Information x Motion | -0.27 | -0.42 – -0.13 | -3.78 | **<0.001** | 0.28 | -0.02 – 0.58 | 1.83 | 0.067 | -0.13 | -0.35 – 0.10 | -1.09 | 0.276 | -0.09 | -0.25 – 0.08 | -1.03 | 0.304 | 0.18 | -0.01 – 0.38 | 1.82 | 0.069 |
| **Random Effects** | | | | | | | | | | | | | | | | | | | | |
| σ^2^ | 0.45 | | | | 0.72 | | | | 0.71 | | | | 0.58 | | | | 0.57 | | | |
| τ_00_ | 1.20 _sid_ | | | | 0.77 _sid_ | | | | 0.96 _sid_ | | | | 1.39 _sid_ | | | | 1.24 _sid_ | | | |
|  | 0.00 _itemno_ | | | | 0.01 _itemno_ | | | | 0.00 _itemno_ | | | | 0.00 _itemno_ | | | | 0.00 _itemno_ | | | |
| ICC |  | | | | 0.52 | | | | 0.58 | | | |  | | | | 0.68 | | | |
| N | 198 _sid_ | | | | 198 _sid_ | | | | 198 _sid_ | | | | 198 _sid_ | | | | 198 _sid_ | | | |
|  | 16 _itemno_ | | | | 16 _itemno_ | | | | 16 _itemno_ | | | | 16 _itemno_ | | | | 16 _itemno_ | | | |
| Observations | 3168 | | | | 3168 | | | | 3167 | | | | 3168 | | | | 3167 | | | |
| Marginal R^2^ / Conditional R^2^ | 0.059 / NA | | | | 0.034 / 0.537 | | | | 0.020 / 0.586 | | | | 0.051 / NA | | | | 0.021 / 0.692 | | | |

|  | **enraptured** | | | | **enlightened** | | | | **interested** | | | | **inspired** | | | | **pleasure** | | | | **upset** | | | |
| --- | --- | --- | --- | --- | --- | --- | --- | --- | --- | --- | --- | --- | --- | --- | --- | --- | --- | --- | --- | --- | --- | --- | --- | --- |
| *Predictors* | *Estimates* | *CI* | *Statistic* | *p* | *Estimates* | *CI* | *Statistic* | *p* | *Estimates* | *CI* | *Statistic* | *p* | *Estimates* | *CI* | *Statistic* | *p* | *Estimates* | *CI* | *Statistic* | *p* | *Estimates* | *CI* | *Statistic* | *p* |
| (Intercept) | 2.87 | 2.68 – 3.05 | 30.90 | **<0.001** | 2.92 | 2.76 – 3.09 | 34.68 | **<0.001** | 3.43 | 3.28 – 3.59 | 44.06 | **<0.001** | 3.09 | 2.91 – 3.26 | 34.15 | **<0.001** | 2.97 | 2.78 – 3.16 | 30.54 | **<0.001** | 2.21 | 2.04 – 2.38 | 25.57 | **<0.001** |
| Content Information | -0.04 | -0.21 – 0.12 | -0.49 | 0.623 | 0.15 | 0.03 – 0.28 | 2.39 | **0.017** | 0.13 | -0.03 – 0.28 | 1.64 | 0.101 | 0.15 | -0.01 – 0.30 | 1.89 | 0.059 | 0.26 | 0.08 – 0.45 | 2.74 | **0.006** | -0.25 | -0.36 – -0.14 | -4.30 | **<0.001** |
| Artist Information | 0.14 | -0.01 – 0.29 | 1.77 | 0.076 | 0.22 | 0.10 – 0.33 | 3.68 | **<0.001** | 0.22 | 0.08 – 0.37 | 3.04 | **0.002** | 0.32 | 0.18 – 0.46 | 4.50 | **<0.001** | 0.50 | 0.32 – 0.67 | 5.56 | **<0.001** | -0.37 | -0.48 – -0.27 | -6.94 | **<0.001** |
| Technique Information | 0.08 | -0.09 – 0.24 | 0.93 | 0.351 | 0.17 | 0.05 – 0.30 | 2.72 | **0.007** | 0.18 | 0.02 – 0.33 | 2.25 | **0.025** | 0.21 | 0.06 – 0.37 | 2.79 | **0.005** | 0.29 | 0.10 – 0.48 | 2.98 | **0.003** | -0.26 | -0.38 – -0.15 | -4.50 | **<0.001** |
| Motion | 0.04 | -0.17 – 0.26 | 0.37 | 0.713 | -0.10 | -0.27 – 0.06 | -1.24 | 0.214 | 0.15 | -0.05 – 0.35 | 1.44 | 0.149 | -0.06 | -0.25 – 0.14 | -0.55 | 0.582 | -0.19 | -0.44 – 0.05 | -1.54 | 0.124 | 0.39 | 0.24 – 0.54 | 5.10 | **<0.001** |
| Age | 0.15 | -0.00 – 0.29 | 1.90 | 0.057 | 0.14 | -0.01 – 0.29 | 1.87 | 0.062 | 0.11 | -0.00 – 0.23 | 1.91 | 0.057 | 0.03 | -0.12 – 0.18 | 0.42 | 0.673 | 0.08 | -0.07 – 0.23 | 1.07 | 0.285 | 0.08 | -0.07 – 0.24 | 1.03 | 0.301 |
| Education | 0.01 | -0.14 – 0.16 | 0.14 | 0.889 | 0.04 | -0.11 – 0.19 | 0.53 | 0.594 | 0.08 | -0.03 – 0.20 | 1.38 | 0.166 | 0.08 | -0.07 – 0.23 | 1.03 | 0.302 | 0.06 | -0.09 – 0.21 | 0.81 | 0.418 | -0.08 | -0.24 – 0.07 | -1.04 | 0.297 |
| Content Information x Motion | -0.05 | -0.38 – 0.28 | -0.28 | 0.779 | 0.15 | -0.10 – 0.40 | 1.20 | 0.232 | -0.13 | -0.43 – 0.18 | -0.80 | 0.426 | 0.14 | -0.16 – 0.45 | 0.94 | 0.347 | 0.36 | -0.02 – 0.74 | 1.88 | 0.060 | -0.54 | -0.76 – -0.31 | -4.60 | **<0.001** |
| Artist Information x Motion | 0.12 | -0.19 – 0.42 | 0.76 | 0.445 | 0.32 | 0.09 – 0.55 | 2.70 | **0.007** | 0.14 | -0.15 – 0.42 | 0.93 | 0.350 | 0.34 | 0.06 – 0.62 | 2.37 | **0.018** | 0.34 | -0.01 – 0.69 | 1.92 | 0.055 | -0.33 | -0.54 – -0.12 | -3.05 | **0.002** |
| Technique Information x Motion | 0.05 | -0.28 – 0.38 | 0.30 | 0.763 | 0.19 | -0.06 – 0.44 | 1.53 | 0.127 | 0.07 | -0.23 – 0.38 | 0.48 | 0.635 | 0.14 | -0.16 – 0.44 | 0.92 | 0.359 | 0.25 | -0.13 – 0.63 | 1.31 | 0.190 | -0.39 | -0.62 – -0.16 | -3.36 | **0.001** |
| **Random Effects** | | | | | | | | | | | | | | | | | | | | | | | | |
| σ^2^ | 0.68 | | | | 0.64 | | | | 0.68 | | | | 0.67 | | | | 0.68 | | | | 0.55 | | | |
| τ_00_ | 1.11 _sid_ | | | | 1.06 _sid_ | | | | 0.67 _sid_ | | | | 1.11 _sid_ | | | | 1.08 _sid_ | | | | 1.19 _sid_ | | | |
|  | 0.01 _itemno_ | | | | 0.00 _itemno_ | | | | 0.01 _itemno_ | | | | 0.01 _itemno_ | | | | 0.01 _itemno_ | | | | 0.00 _itemno_ | | | |
| ICC | 0.62 | | | | 0.63 | | | | 0.50 | | | | 0.63 | | | | 0.62 | | | | 0.69 | | | |
| N | 198 _sid_ | | | | 198 _sid_ | | | | 198 _sid_ | | | | 198 _sid_ | | | | 198 _sid_ | | | | 198 _sid_ | | | |
|  | 16 _itemno_ | | | | 16 _itemno_ | | | | 16 _itemno_ | | | | 16 _itemno_ | | | | 16 _itemno_ | | | | 16 _itemno_ | | | |
| Observations | 3168 | | | | 3168 | | | | 3168 | | | | 3168 | | | | 3168 | | | | 3168 | | | |
| Marginal R^2^ / Conditional R^2^ | 0.015 / 0.627 | | | | 0.018 / 0.633 | | | | 0.025 / 0.512 | | | | 0.014 / 0.631 | | | | 0.026 / 0.627 | | | | 0.024 / 0.695 | | | |

Table S
